# Disentangling the Switching Behavior in Functional Connectivity Dynamics in Autism Spectrum Disorder: Insights from Developmental Cohort Analysis and Molecular‐Cellular Associations

**DOI:** 10.1002/advs.202403801

**Published:** 2025-05-09

**Authors:** Wei Li, Xia Qiu, Jin Chen, Kexuan Chen, Meiling Chen, Yinyan Wang, Wenjie Sun, Jing Su, Yongchang Chen, Xiaobao Liu, Congying Chu, Jiaojian Wang

**Affiliations:** ^1^ State Key Laboratory of Primate Biomedical Research Institute of Primate Translational Medicine Kunming University of Science and Technology Kunming 650500 China; ^2^ Faculty of Mechanical and Electrical Engineering Kunming University of Science and Technology Kunming 650500 China; ^3^ Medical School Kunming University of Science and Technology Kunming 650500 China; ^4^ Department of Clinical Psychology the First People's Hospital of Yunnan Province The Affiliated Hospital of Kunming University of Science and Technology Kunming 650500 China; ^5^ Department of Neurosurgery Beijing Tiantan Hospital Capital Medical University Beijing 100070 China; ^6^ Brainnetome Center & National Laboratory of Pattern Recognition Institute of Automation Chinese Academy of Sciences Beijing 100190 China; ^7^ Yunnan Key Laboratory of Primate Biomedical Research Kunming 650500 China

**Keywords:** autism spectrum disorder, brain dynamics, development, molecular mechanisms, resting‐state fMRI

## Abstract

Characterizing the transition or switching behavior between multistable brain states in functional connectivity dynamics (FCD) holds promise for uncovering the underlying neuropathology of Autism Spectrum Disorder (ASD). However, whether and how switching behaviors in FCD change in patients with developmental ASD, as well as their cellular and molecular basis, remains unexplored. This study develops a region‐wise FCD switching index (RFSI) to investigate the drivers of FCD. This work finds that brain regions within the salience, default mode, and frontoparietal networks serve as abnormal drivers of FCD in ASD across different developmental stages. Additionally, changes in RFSI at different developmental stages of ASD correlated with transcriptomic profiles and neurotransmitter density maps. Importantly, the abnormal RFSI identifies in humans has also been observed in genetically edited ASD monkeys. Finally, single‐nucleus RNA sequencing data from patients with developmental ASD are analyzed and aberrant switching behaviors in FCD may be mediated by somatostatin‐expressing interneurons and altered differentiation patterns in astrocyte State2. In conclusion, this study provides the first evidence of abnormal drivers of FCD across different stages of ASD and their associated cellular and molecular mechanisms. These findings deepen the understanding of ASD neuropathology and offer valuable insights into treatment strategies.

## Graphical abstract

A region‐wise functional connectivity dynamics (FCD) switching index (RFSI) identifies abnormal drivers in ASD across development, linked to salience/ventral attention, default mode, and frontoparietal networks. RFSI changes correlate with transcriptomic and neurotransmitter profiles, and supported by gene‐edited ASD monkeys. Single‐nucleus RNA sequencing provide novel insights into the cellular and molecular mechanisms underlying ASD.

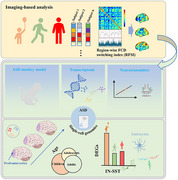

## 1 Introduction

The traditional view assumes that the functional couplings between brain areas are constant;^[^ ^1^ ^]^ however, mounting evidence has demonstrated that the brain is a complex, multiscale dynamic system exhibiting various spatiotemporal patterns.^[^ ^2^, ^3^, ^4^, ^5^ ^]^ Quantifying the instantaneous changes of functional connectivity dynamics could contribute to a deeper understanding the functioning of brain.^[^ ^6^, ^7^, ^8^, ^9^ ^]^ The time series of overlapping networks can induce variations in functional connectivity between regions, influenced by the impact of specific networks. Within a multivariate time series, a certain degree of multi‐stability exists in the patterns. Recent studies suggest that whole‐brain functional connectivity dynamics may be caused by switching between multi‐stable states^[^ ^10^, ^11^ ^]^ and this intriguing property of spontaneous ebb and flow has been shown to be driven by the sensory‐motor cortices.^[^ ^12^ ^]^ This switching behavior plays a crucial role in indicating potential changes in macroscopic neural activity patterns associated with cognitive and behavioral processes.

Autism spectrum disorder (ASD) is a neurodevelopmental disorder characterized by persistent impairment of social and communication functions, restricted interests, and repetitive behaviors.^[^ ^13^, ^14^ ^]^ Numerous research studies have demonstrated that individuals with ASD exhibit abnormal brain dynamics, manifesting increased variability in connectivity,^[^ ^15^, ^16^, ^17^, ^18^ ^]^ and aberrant switching behaviors between connectivity states.^[^ ^19^, ^20^, ^21^, ^22^ ^]^ However, the brain regions with aberrant switching patterns and spatial changes from driving regions to spreading regions remain unknown. Tracking the spatial patterns of aberrant switching behaviors provides insight into the aberrant brain regions of patients with ASD in the integration between different functional systems and cognitive flexibility.

Understanding and exploring the neurobiological mechanisms underlying ASD that lead to changes in switching behaviors and developmental trajectories are overwhelming challenges. Currently, nonhuman primates, which collectively share more genotypic and phenotypic identities with humans than any other model organism, are valuable tools for the study of human genetic diseases.^[^ ^23^ ^]^ For complex behavioral phenotypes, such as anxiety;^[^ ^24^, ^25^, ^26^ ^]^ ASD;^[^ ^27^, ^28^ ^]^ and cardiovascular phenotypes, including heart disease,^[^ ^29^, ^30^ ^]^ obesity and type 2 diabetes,^[^ ^31^ ^]^ and hereditary cancers,^[^ ^32^, ^33^ ^]^ nonhuman primate models have been employed as genetic models for diseases with substantial hereditary components. Furthermore, whole‐brain gene expression maps from the Allen Human Brain Atlas (AHBA) offer the potential opportunity to establish links between neuroimaging phenotypes and gene expression profiles.^[^ ^34^ ^]^ In addition to AHBA transcriptomic data, positron emission tomography imaging technology is used to generate high‐resolution neurotransmitter density maps of the human brain.^[^ ^35^, ^36^, ^37^, ^38^ ^]^ These maps constructed stable and reproducible whole brain distribution maps for neurotransmitter receptors and transporters. The shared transcriptome and neurotransmitter enable the elucidation of the molecular basis and related neurotransmitters underlying structural and functional alterations in brain disorders. As the AHBA transcriptome only measures the average gene expression across cells in a tissue and not directly from the patients, the reliability of the identified genes associated with specific brain disorders remains controversial. Moreover, given the individual complexity of each cell and tissue heterogeneity, single‐cell sequencing technology for brain tissues from enrolled patients and controls may better reveal the cellular and molecular bases of brain disorders.^[^ ^39^ ^]^ Therefore, integrating brain imaging phenotypes, transcriptomes, neurotransmitters, and single‐cell sequencing data could provide comprehensive evidence for the neuropathology of ASD.

**Figure 1:**
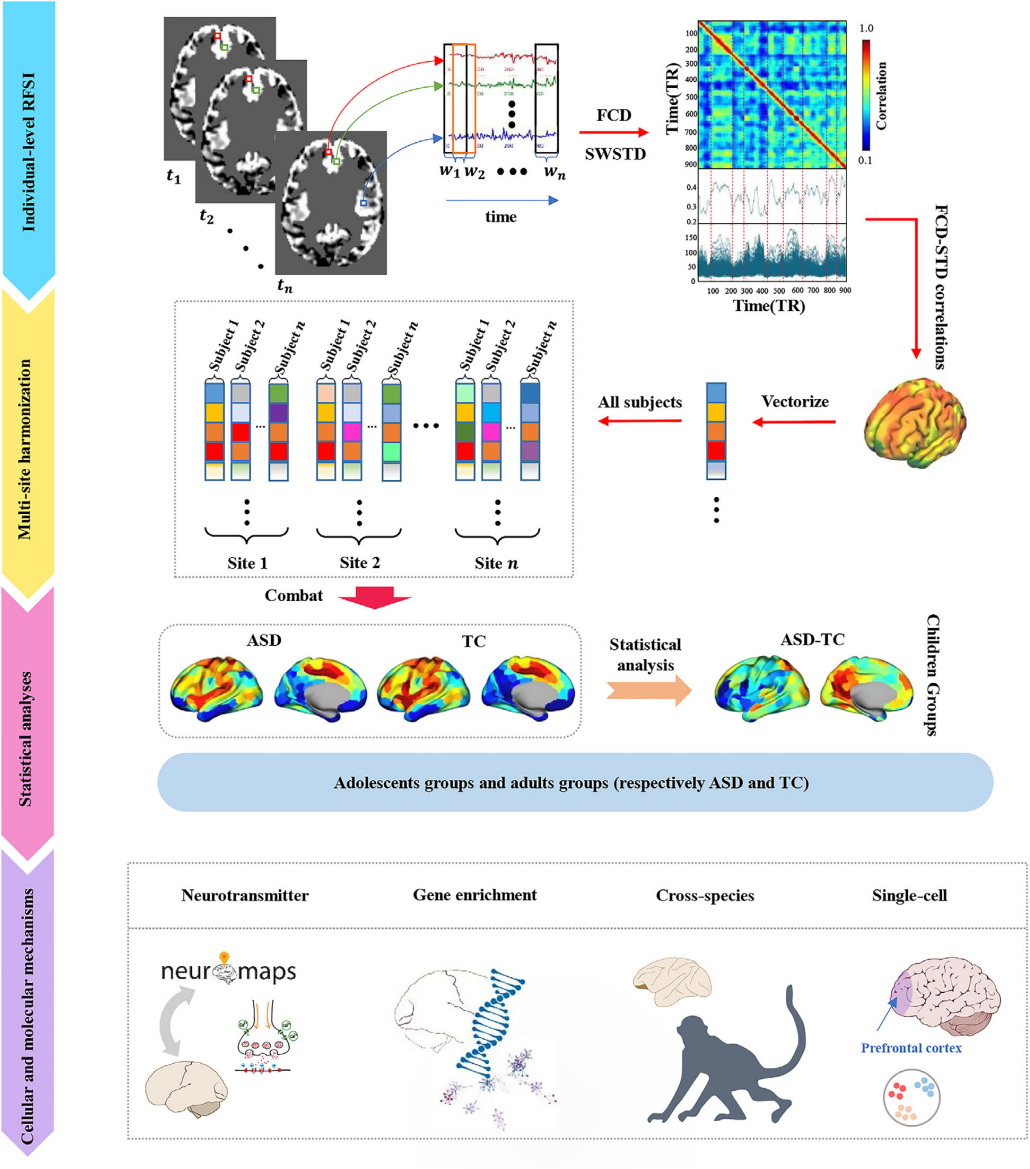
Overview of the method pipeline. First, calculation of RFSI at the individual level. Second, harmonization of multi‐site data. Third, categorization of subjects into children, adolescents, and adults based on age and into ASD and typical control TC groups based on disease status. Finally, exploring the cellular and molecular mechanisms underlying aberrant switching behaviors. Partial brain map from https://scidraw.io/.^[^ ^40^, ^41^, ^42^, ^43^, ^44^ ^]^

In this study, we aimed to investigate aberrant switching behaviors in functional connectivity dynamics across different developmental stages and reveal the cellular and molecular basis using resting‐state fMRI data from a large sample of 1700 participants on the Autism Brain Imaging Data Exchange I and II (ABIDE I and ABIDE II). First, we calculated the region‐wise FCD switching index (RFSI) for each individual to capture the switching behavior of the cerebral cortex. Second, a two‐way multivariate analysis of variance (MANOVA) was used to identify differences in RFSI between patients with ASD and typical controls (TC) and between children, adolescents, and adults. Aberrant switching behaviors observed in humans were further validated using ASD monkey models. Third, the relationships between changes in RFSI and clinical phenotypes, the AHBA transcriptome, and neurotransmitter density maps were explored. Finally, single‐nucleus RNA sequencing (snRNA‐seq) data of the prefrontal cortex (PFC) of ASD and TC from children to adults were employed to reveal the specific cellular and molecular differences between ASD and TC and between different age groups of ASD. A detailed flowchart of the study is shown in **Figure** **1**.

## 2 Results

### 2.1 Abnormal RFSI across ASD

The FCD has been reported to have two states: a high‐coherence state induced by the coherence of the sliding time window over a long period of time in the FC pattern and a low‐coherence state induced by the sliding‐window FC pattern driven by fMRI signals and autocorrelation in the overlapping sliding window.^[^ ^45^ ^]^ We also found two similar states in healthy controls and patients with ASD (Figure S1A, Supporting Information). A significant correlation was observed between the cortical mean FCD and sliding window standard deviation (SWSTD) (Figure S1B, Supporting Information). The mean time course of the FCD reflected cortical‐wide fluctuations in the FC pattern, whereas the time course of the SWSTD was region‐specific. Based on this characteristic, we captured the switching behavior of the whole‐brain dynamics.

**Figure 2:**
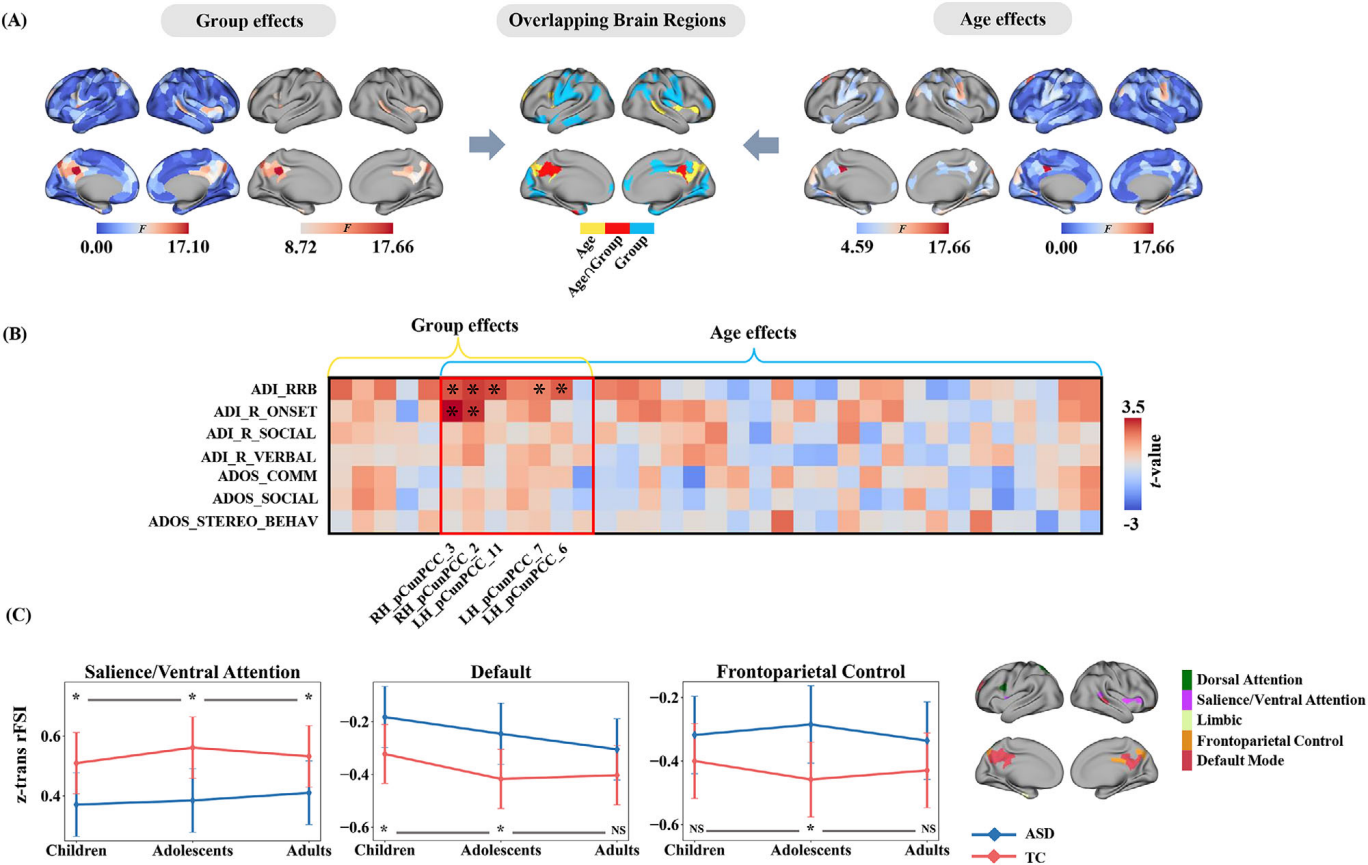
MANOVA revealed the abnormal RFSI. A) The Age and Group effects mapping to the cerebral cortex, where Age and Group effects were FDR‐corrected *p* < 0.05 corrected to obtain mapping maps with thresholds. The red areas of the centermost map indicate brain regions where Age and Group effects overlap after FDR correction (children: 253 ASD and 345 TC; adolescents: 274 ASD and 283 TC; adults: 293 ASD and 252 TC; MANOVA). B) Functional connectivity dynamic deficits associated with clinical symptoms, illustrating differences in RFSI in the brains of ASD with severe (above mean) and mild (below mean) conditions (unpaired two‐sample *t*‐tests). Each row of the matrix heat map represents the ASD behavioral scale, each column represents the age versus group FDR corrected *p* < 0.05 surviving brain regions, and * indicates the significant differences between severely and mildly affected patients in a region (FDR corrected *p* < 0.05). C) Developmental trajectories of brain dynamics. Changes in children, adolescents, and adults were calculated based on the Yeo 7 network partitioned into Group effect threshold map. Line graphs depict the trend of the mean *z*‐values of ASD and TC across the three networks. * Indicates a significant difference in unpaired two‐sample *t*‐tests (Bonferroni corrected *p* < 0.05), while NS indicates no significant difference. Error bar, 95% confidence intervals for mean.

Using MANOVA, we found significant age‐related differences in the switching behavior of the cortex, including the medial prefrontal cortex, posterior cingulate cortex, temporal lobe, superior frontal gyrus, and orbitofrontal cortex (Age effects spatial map, false discovery rate (FDR) corrected *p* < 0.05; **Figure** **2**A). Significant group differences (ASD versus TC) were observed in the bilateral superior frontal gyrus, anterior cingulate cortex, left posterior cingulate cortex, and right insula (Group effects spatial map, FDR‐corrected *p* < 0.05; Figure 2A). Overlapping regions of variability were observed in the Age effect and Group effect *F*‐maps, particularly in the default mode network (DMN), which may be potential factors contributing to the development of this disorder.

### 2.2 Abnormal RFSI in Severe and Mild ASD

Analysis of the significantly altered brain regions using behavioral scales revealed significant differences in RFSI between severe and mild ASD associated with restricted, repetitive, and stereotyped behaviors (RRB) in the DMN (Figure 2B; Figure S2B, Supporting Information). Furthermore, significant differences linked to the ADI_R_ONSET scale were observed in the right precuneus/posterior cingulate cortex (pCun/PCC) region (Figure 2B, Figure S2C, Supporting Information).

### 2.3 Developmental Trajectories of Cortical Dynamics in Brain Areas Associated with ASD

To elucidate age‐dependent alterations in cortical dynamics associated with ASD, we examined the developmental trajectories of RFSI in brain regions exhibiting significant Group effects (ASD versus TC), as determined by FDR‐corrected F‐maps. Brain regions exhibiting significant group differences were mapped onto the Yeo 7 resting‐state subnetwork,^[^ ^46^ ^]^ to investigate the developmental trajectories of ASD at the network level. These aberrant regions were distributed across five functional networks: Dorsal Attention, Salience/Ventral Attention, Limbic, Frontoparietal Control, and Default Mode. Group comparisons using unpaired two‐sample *t*‐tests revealed that the salience/ventral attention network exhibited robust and consistent group differences across all age groups (*t* = −3.19, *p*_adj_ = 0.005, Cohen's *d* = 0.16), adolescents (*t* = −3.87, *p*_adj_ < 0.001, Cohen's d = 0.20), and adults (*t* = −2.66, *p*_adj_ = 0.024, Cohen's *d* = 0.14), suggesting a persistent alteration in attentional salience processing throughout ASD development. For the DMN, significant differences were restricted to childhood and adolescence (children: *t* = 2.81, *p*_adj_ = 0.015, Cohen's *d* = 0.14; adolescents: *t* = 3.33, *p*_adj_ = 0.003, Cohen's *d* = 0.17; adults: *t* = 2.01, *p*_adj_ = 0.136, Cohen's *d* = 0.10), implying potential normalization or compensatory mechanisms in adulthood. Conversely, the frontoparietal control network demonstrated a significant group difference exclusively during adolescence (children: *t* = 1.81, *p*_adj_ = 0.213, Cohen's *d* = 0.08; adolescents: *t* = 3.82, *p*_adj_ < 0.001, Cohen's *d* = 0.17; adults: *t* = 2.00, *p*_adj_ = 0.138; Cohen's *d* = 0.09), indicating a transient, however, developmentally specific disruption in cognitive control processes. Collectively, these findings underscore the spatiotemporal heterogeneity of network‐level functional alterations in ASD and highlight adolescence as a critical window for frontoparietal dysfunction.

In addition, we conducted statistical comparisons of the RFSI scores between the ASD and TC groups across different developmental stages. The results revealed a gradual reduction in RFSI differences from childhood to adulthood in the precuneus, left middle frontal gyrus, and right superior and middle temporal gyri, whereas RFSI differences in the inferior parietal lobule progressively increased (Figure S3B, Supporting Information). To evaluate the similarity of the spatial patterns across age stages, we performed spatial Pearson correlation analyses on statistical *t*‐value maps for each pairwise age comparison (Figure S3C, Supporting Information). The strongest correlation was observed between childhood and adolescence (*r* = 0.53), whereas the weakest correlation was found between childhood and adulthood (*r* = 0.15). These results underscore that adolescence is a critical transitional period for functional reorganization in individuals with ASD.

### 2.4 Abnormal RFSI Associated with Behaviors and Cognitions

**Figure 3:**
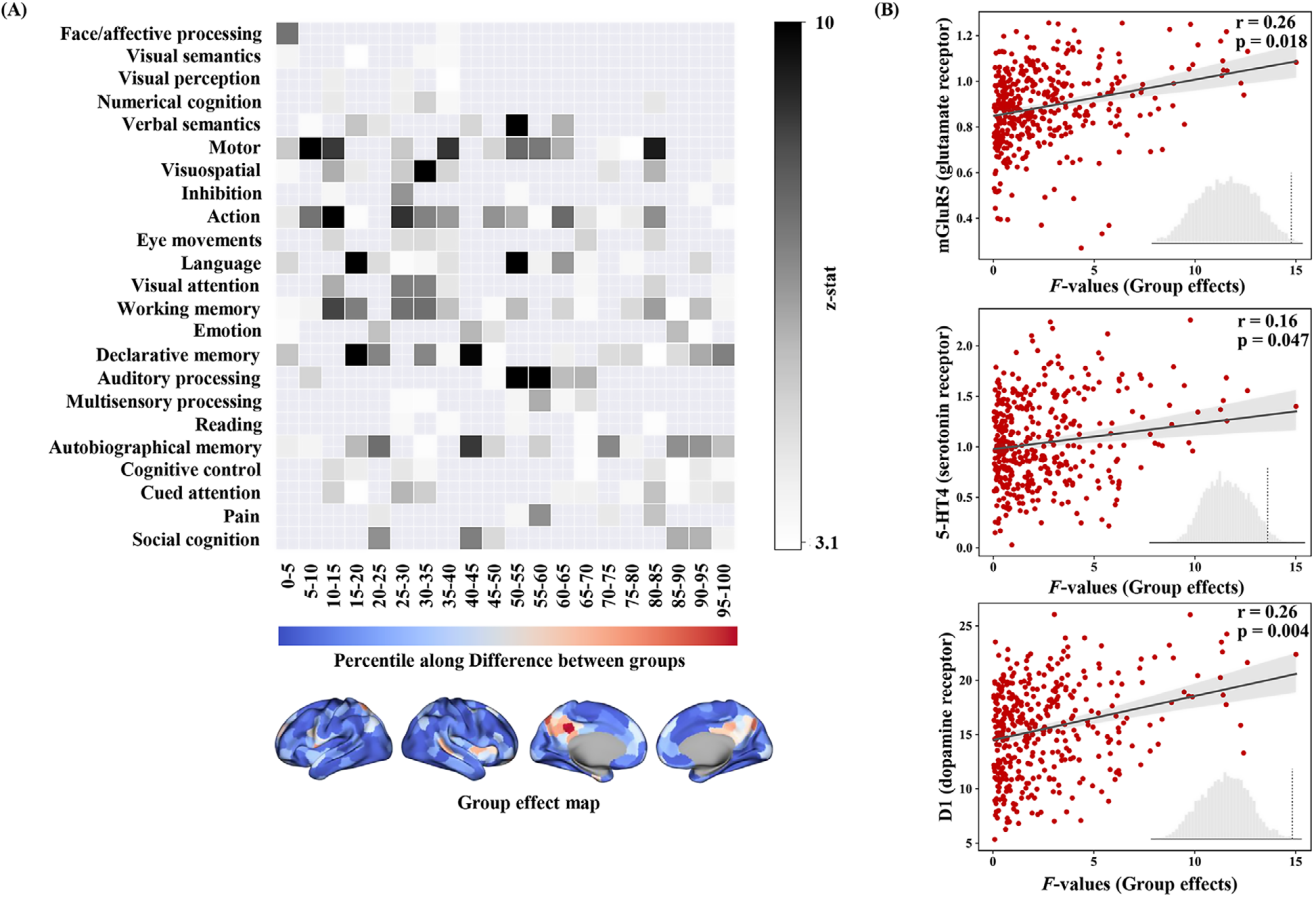
Interpretation of abnormal RFSI A) Decoding of Group effect in Brain Function. Relationships between Group effect *F*‐map and 24 cognitive components based on the NeuroSynth meta‐analysis database. Each row indicates that the components were sorted from left to right by five percent increments of the *F*‐statistic and each column indicates that cognitive components were sorted by the weighted average of the resulting *z*‐statistic values. B) Spatial correlation analysis of ASD brain variability with neurotransmitter receptor density maps. Spin‐test was used to test for significance and the spatial correlation was interpreted by rotating the Group effect *F*‐map 10 000 times.

Our analyses further determined the abnormal Group effect *F*‐map associated with behavioral and cognitive functions. **Figure** **3**A shows a heat map in which the weighted means of the *z*‐statistics associated with the cognitive components are sorted by rows. Regions with lower *F*‐values were associated with vision and motor functions and regions with higher *F*‐values in the DMN were involved in social cognition, declarative memory, and autobiographical memory. In addition, we analyzed the Age effect *F*‐map, which showed a similar gradient change from visual, verbal, and motor to higher cognitive functions (Figure S4, Supporting Information).

### 2.5 Neurotransmitters Associated with Changes in Brain Dynamics in ASD

Neurotransmitter receptor density map‐neuroimaging spatial correlation analysis showed that the abnormal RFSI of the ASD brain region (Group effect *F*‐map) was significantly and positively correlated with the distribution patterns of glutamate (*r* = 0.22, *p*_spin_ = 0.039), dopamine (*r* = 0.26, *p*_spin_ = 0.007), and 5HT4 (*r* = 0.16, *p*_spin_ = 0.048) (Figure 3B).

### 2.6 Transcriptional Correlations with RFSI Changes in ASD

We assessed the spatial association between changes in RFSI (Group effect *F*‐map) and regional gene expression profiles using partial least square (PLS) regression. We focused on the first two components (PLS1 and PLS2), with the first component (PLS1) defined as capturing the spatial variance of the largest portion of total gene expression in the cortical regions (explaining 15% of the variance; Figure S5A,B, Supporting Information). Both the PLS1 and PLS2 scores were positively correlated with the Group effect *F*‐map (*r*_1_ = 0.41, *p*_spin_ < 0.005; *r*_2_ = 0.31, *p*_spin_ < 0.005; Randomly “rotated” the Group effect *F*‐map to account for spatial correlations; Figure S5C, D, Supporting Information).

Normalized weights of PLS1 were ranked using a univariate one‐sample *z*‐test. We found 773 PLS1+ (*z* > 2.8) and 828 PLS1‐ (*z* < −2.8) (FDR‐corrected *p* < 0.05) positively (or negatively) weighed genes associated with the Group effect *F*‐map. Only 773 positively associated genes were included in the Gene Ontology (GO) enrichment analysis to identify important biological processes associated with regional abnormalities of RFSI in ASD. After correcting for enrichment terms (FDR‐corrected *p* < 0.05) and excluding discontinuous enrichment clusters, the top 12 notable biological processes associated with autism were enriched, including synaptic signaling, regulation of neurotransmitter levels, positive regulation of growth, neurological developmental regulation, and regulation of hormone levels (Figure S5E, Supporting Information).

### 2.7 Similar RFSI Differences in ASD Macaques

**Figure 4:**
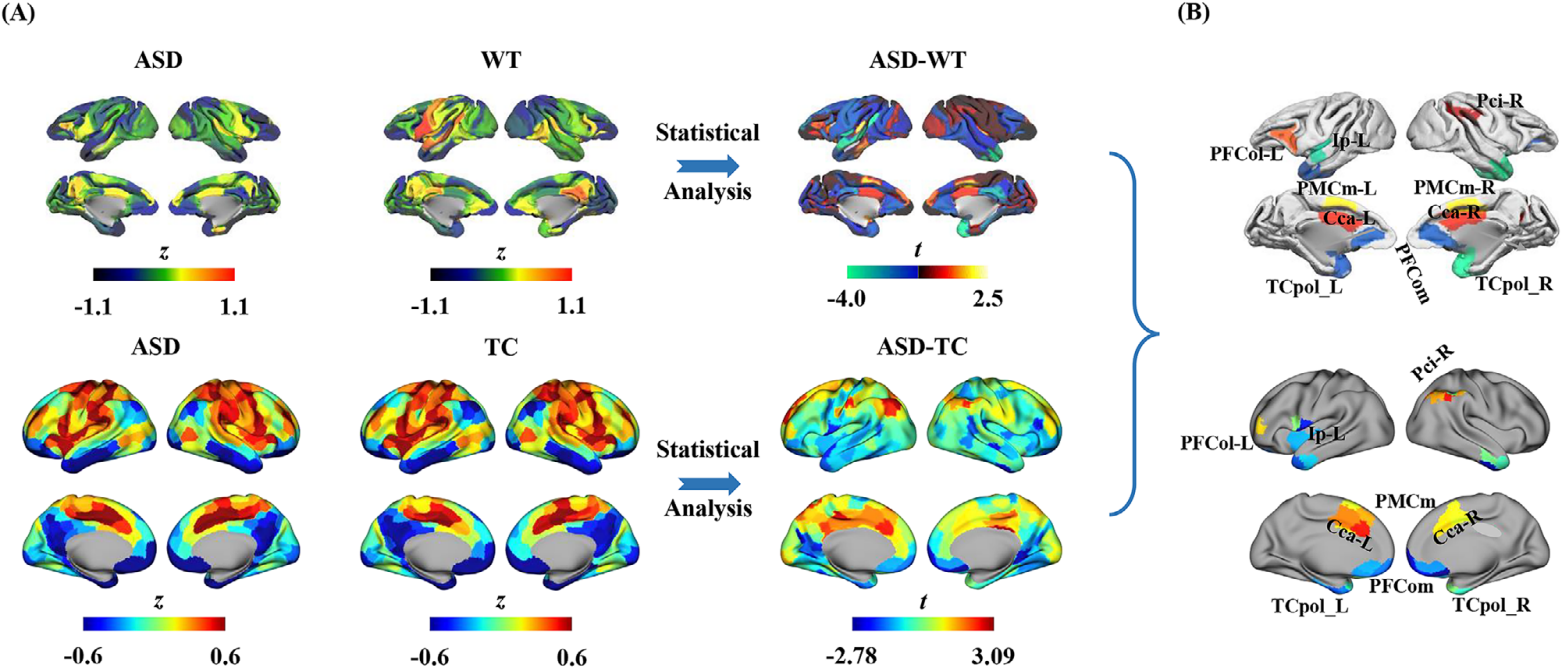
Similarity analysis of brain RFSI differences between ASD macaques and adults with ASD. A) The RFSI for ASD monkey models (ASD) and wild type (WT) monkeys at the group level, as well as spatial *t*‐map of ASD‐WT differences are shown (unpaired two‐sample *t*‐tests). B) Comparison of similar regions in the pattern of differences between ASD‐WT in monkeys and ASD‐TC in adults.

The abnormal RFSI found in humans with ASD has been validated using ASD monkey models. Similarly, we calculated the RFSI in monkeys in the ASD and WT groups and unpaired two‐sample *t*‐tests were performed to identify between‐group differences. We found that significant differences in RFSI in macaques with ASD were located in the cingulate gyrus and the lateral prefrontal and temporal pole regions (**Figure** **4**A). The difference in the pattern in macaques was similar to that in human adults with ASD (Figure 4A; Figure S3B, Supporting Information). The brain areas showing similar RFSI differences in both humans and macaques ASD included the left orbitolateral prefrontal cortex (PFCol), left posterior insula (Ip), right inferior parietal cortex (PCi), bilateral medial premotor cortex (PMCm), anterior cingulate cortex (CCa), orbitomedial prefrontal cortex (PFCom), and temporal polar cortex (TCpol) (Figure 4B).

### 2.8 Linking Altered Macroscopic Brain Dynamics to Cell Type‐Specific Molecular Changes

**Figure 5:**
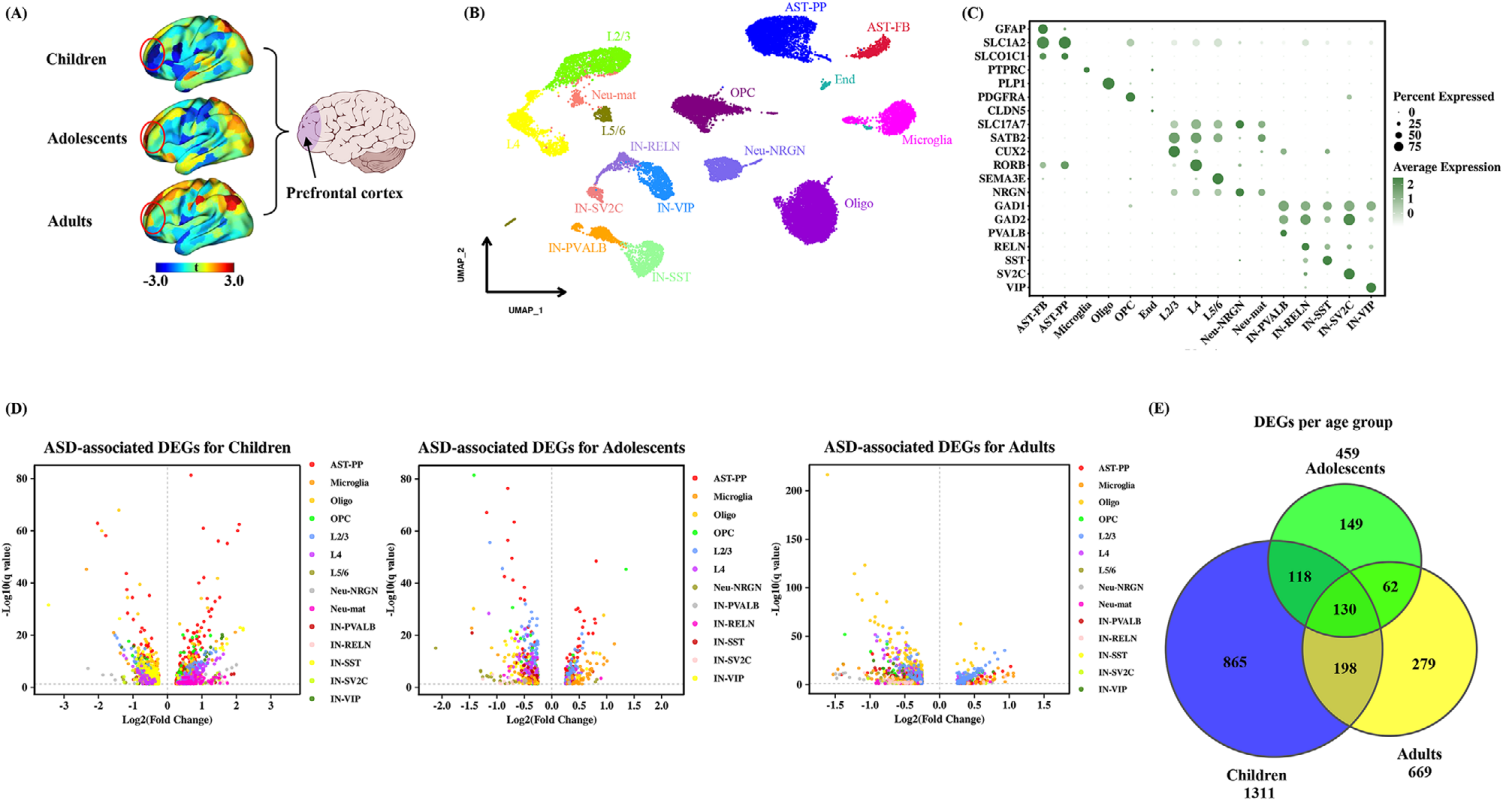
From macro‐cortical abnormal dynamics to cell type‐specific gene expression. A) Differences in the left dorsolateral prefrontal RFSI between the ASD and TC groups in children, adolescents, and adults (unpaired two‐sample *t*‐tests). B) Clustering of snRNA‐seq data using the SSN algorithm and cell types were annotated according to the expression of known marker genes. Key annotations: OPC, Oligodendrocyte precursor cells; Oligo, Oligodendrocytes; AST‐PP, Protoplasmic astrocytes; AST‐FB, Fibrous astrocytes; Microglia, Microglia cell; End, Endothelial; Neu‐NRGN, NRGN‐expressing neurons; Nue‐mat; Maturing neurons; IN‐SST, Somatostatin interneurons; IN‐SV2C, SV2C interneurons; IN‐VIP, VIP interneurons; IN‐PVALB, Parvalbumin interneuron; IN‐RELN, Reelin interneuron; L2/3, Layer 2/3 excitatory neurons; L4, Layer 4 excitatory neurons; and L5/6, Layer 5/6 projection neurons. C) Dot plots showing the expression levels of specific marker genes for each cell type with a color gradient from dark green to light green indicating high to low levels of gene expression. The dot size indicates the percentage of cells in the cluster that express the gene. D) Volcano plots of differentially expressed cell type‐specific genes in the children, adolescents, and adults, with each volcano showing the top five differentially expressed genes. E) Venn diagram indicating the number of differential genes in the child, adolescent, and adult groups (Wilcoxon rank sum test, Bonferroni *p* < 0.05).

Based on the aforementioned findings, we observed that patients with ASD exhibited an abnormal RFSI in the PFC during childhood (**Figure** **5**A; Figure S3A,B, Supporting Information). In addition, we noted that the RFSI difference in the PFC exhibited contrary patterns in children compared with that in adolescents and adults with ASD compared with the TC. Compared with controls, children with ASD showed reduced RFSI in the PFC, whereas adolescents and adults with ASD exhibited increased RFSI.

To delineate the cell type‐specific molecular mechanisms underlying aberrant switching behaviors of the PFC in ASD, shared single‐cell sequencing datasets for the PFC with the 10X Genomics platform were accessed, and these data were classified into children, adolescents, and adults based on the age of the participants. After preprocessing with the Seurat pipeline, removing ambient RNA contamination and excluding doublet cells, we acquired 19 788 high‐quality single‐cell transcriptomes with a median of 4149 unique molecular identifiers (UMIs) and 1918 genes per cell (Figure S6A,B, Supporting Information). Based on predefined cell‐specific markers, we classified all cells into four major categories comprising 16 subtypes: glial cells (Microglia, Oligo, AST‐FB, AST‐PP and OPC), interneurons (IN‐SST, IN‐PVALB, IN‐SV2C, IN‐RELN, and IN‐VIP), excitatory neurons (Neu‐NRGN, Neu‐mat, L2/3, L4, and L5/6), and endothelial cells. (Figure 5B,C; Figure S6C, Supporting Information). All cell types were present across all age groups. In each age group, the number of glial cells was higher than that of neurons (Figure S7, Supporting Information).

Subsequently, a Wilcoxon rank‐sum test was performed between the ASD and TC groups for different cell types in children, adolescents, and adults and genes with *p*‐value < 0.05 were selected as differentially expressed genes (DEGs) for each cell type in each cross‐section (Bonferroni correction; Figure 5D). Based on the number of DEGs at different age stages, the largest number of DEGs was found in childhood, which is consistent with the finding that abnormalities in cortical dynamics are more severe in children with ASD than those in adolescents or adults. The number of DEGs was lowest in adolescents with ASD (Figure 5E).

**Figure 6:**
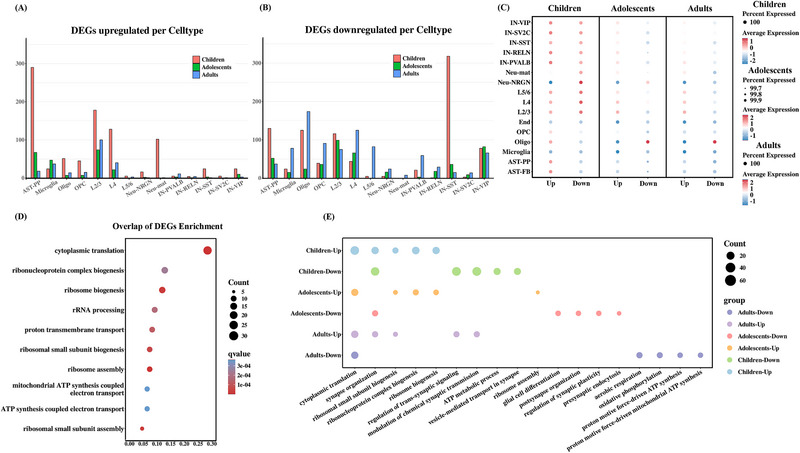
Differential gene enrichment analysis. A) Upregulated DEGs per clusters. B) Downregulated DEGs per cluster. C) Displaying the average expression of specific gene sets within the transects across cell types. D) GO enrichment of overlapping DEGs across age groups. E) GO enrichment of upregulated and downregulated DEGs across age groups. The colors of the circles represent different classifications and the size of the circle represents the number of genes in the corresponding entries.

For the DEGs, the upregulated DEGs were mainly found in AST‐PP, L2/3, and L4 cells, whereas the downregulated DEGs were mainly found in IN‐SST cells in children with ASD. In adolescents and adults with ASD, DEGs exhibited less specificity than in children and these DEGs were found in most cell types (**Figure** **6**A,B). Subsequently, we further identified common DEGs between the ASD and TC groups at each developmental stage, regardless of the cell type. We scored these gene sets across different cell types (Figure 6C). Our analysis revealed that, in adolescents and adults, these common DEG‐associated cell types were almost identical, with upregulated DEGs primarily enriched in neurons, whereas downregulated DEGs were predominantly found in oligodendrocytes. These findings suggest that ASD in adolescence and adulthood is primarily associated with axonal and synaptic pruning and regulation. Conversely, during childhood, upregulated DEGs were enriched across multiple cell types, whereas downregulated DEGs were primarily enriched in neurons, particularly excitatory neurons.

To reveal the common molecular mechanisms underlying ASD at different ages, 130 overlapping DEGs were identified across different age stages. GO enrichment analysis indicated that translational mechanisms (especially ribosome biogenesis) and mitochondrial energy metabolism may play important roles in the molecular mechanisms involved in ASD development. Abnormalities, in ribosome function which may lead to impaired protein synthesis and in the mitochondrial electron transport chain which may affect neuronal energy supply, are important pathological mechanisms involved in ASD (Figure 6D). In addition, we performed functional enrichment for the upregulated and downregulated DEGs specific to each age group (Figure 6E). During childhood and adolescence, we observed that the molecular functions of upregulated genes were mainly involved in ribosome assembly and biosynthetic processes, such as ribonucleoprotein complex biogenesis, ribosome biogenesis, ribosomal small subunit biogenesis, and ribosome biogenesis, whereas the molecular functions of downregulated genes were mainly involved in the regulation of synaptic function and synaptic signaling mechanisms, such as synapse organization, regulation of trans‐synaptic signaling, modulation of chemical synaptic transmission, and vesicle‐mediated transport in the synapse. Conversely, the molecular functions of the upregulated DEGs specifically found in adults were primarily associated with synapses, whereas the downregulated DEGs were mainly associated with intracellular ATP generation. The molecular functions of the upregulated DEGs in adults were found in the enrichment results for the downregulated DEGs in children and adolescents.

### 2.9 Transcription Factor Analysis

**Figure 7:**
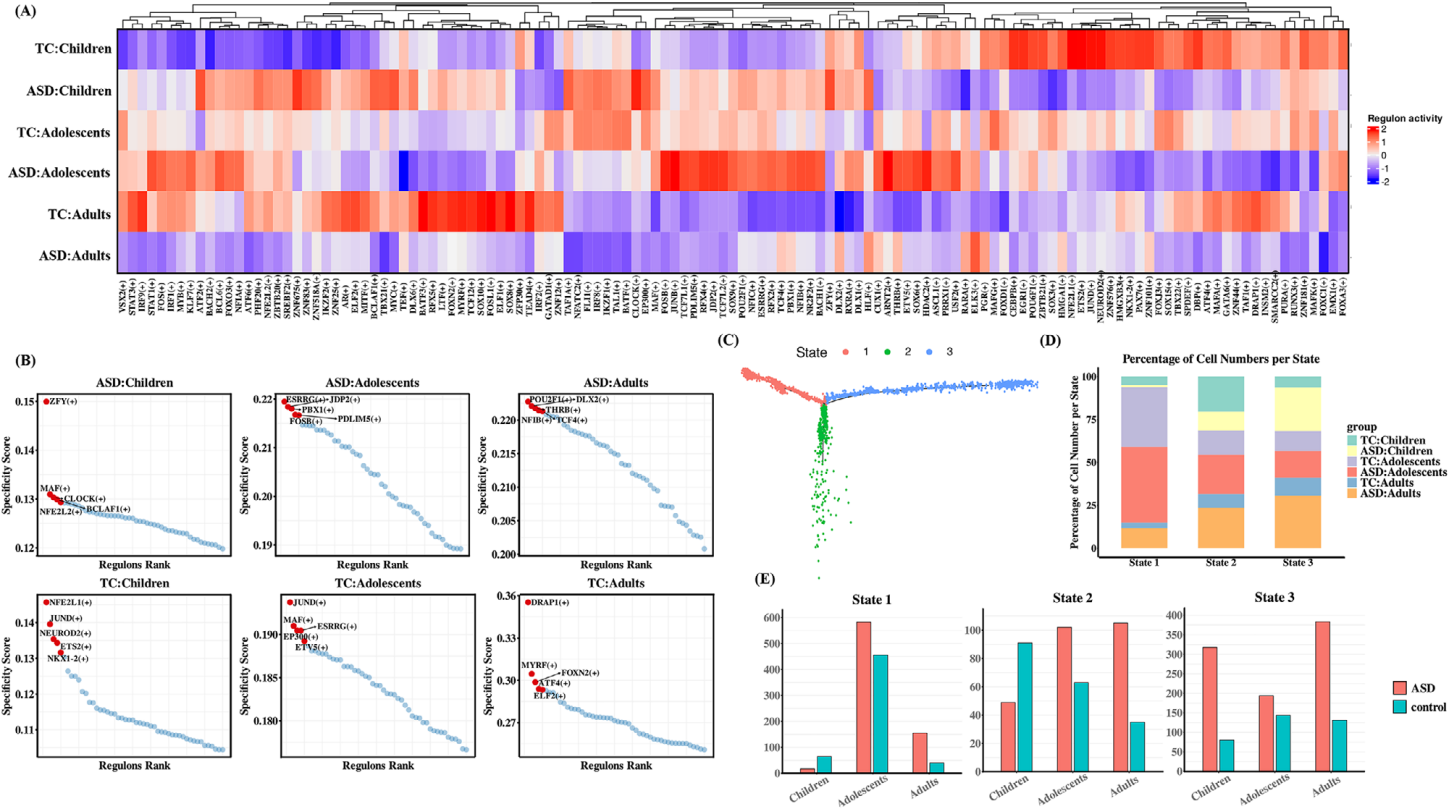
Single‐cell transcription factor analysis and trajectory analysis. A) Heatmap of RAS activity of cell‐level regulons in each group with each column indicating a different regulon and each row indicating a different grouping. The color from blue to red indicates the RAS activity score from low to high. The higher the score of RAS, the stronger the activity of regulon in that group. B) Ranking of RSS scores for each group and labeling of the top five regulon. Regulators with higher RSS may be associated with this age stage specificity. C) Differentiation trajectories of astrocytes. D) Percentage distribution of cell numbers in each age group for the three states. E) Distribution of cell numbers for the three states in each age group in the ASD and TC groups.

ASD is considered to result mainly from an imbalance in the expression of multiple genes, leading to the dysregulation of neural network function. Therefore, investigating the regulatory networks of the upstream transcription factors associated with ASD is crucial. To determine whether ASD at different ages has specific transcription factors (TFs), we analyzed the activity of the Regulon for ASD versus TC in children, adolescents, and adults using the SCENIC method^[^ ^47^ ^]^ and calculated the regulon specificity score (RSS) for each TF in each age group (**Figure** **7**A,B). The results revealed substantial differences in enriched regulons across different age groups. During the childhood stage, the more specific regulons included ZFY, a Y chromosome‐linked factor significantly associated with sex differences in ASD susceptibility; MAF, which is involved throughout the medial ganglionic eminence lineage cycle; and CLOCK, a key regulator of circadian rhythms. In the adolescent stage, distinct regulons include estrogen‐related receptor gamma (ESRRG); JDP2, which primarily participates in RNA polymerase II transcription; and PDLIM5, which plays a role in cardiomyocyte proliferation and inhibition of excitatory synapse postsynaptic growth. In the adult stage, notable regulons include POU2F1, which enables chromatin‐binding activity and sequence‐specific DNA binding and DLX2, which functions as a DNA‐binding transcription factor and is involved in chromatin binding.

### 2.10 Construction of Astrocyte Differentiation Trajectories

Astrocytes have been demonstrated to respond to and promote inflammatory signals, which is a key pathological mechanism in ASD.^[^ ^48^ ^]^ In children with ASD, the upregulated DEGs were mainly expressed in AST‐PP cells. Therefore, we analyzed the developmental trajectories of astrocyte subpopulations by combining ASD and TC to identify cell differentiation states. Cell differentiation trajectory analysis revealed three astrocyte differentiation states: State1, State2, and State3 (Figure 7C; Figure S9A,D, Supporting Information). Because of the inconsistency in sample size across different age groups, we primarily compared the number of cells between ASD and TC within each developmental stage. We observed distinct patterns in the cell number distribution between the ASD and TC across the three states. We found that the distribution patterns of the number of cells between the ASD and TC in State 2 were similar to the neuroimaging findings. In State 2, the number of cells was higher in the TC than that in the ASD group, whereas in adolescents and adults, the number of cells was higher in the ASD group than that in the TC group. Thus, the number of astrocytes in state 2 may serve as a biomarker for distinguishing ASD from TC at different developmental stages.

### 2.11 Validation Analyses

To verify the stability of the RFSI difference patterns across age groups in different window lengths, we chose window lengths of 24 and 36 time points for the validation analyses. The RFSI difference patterns as well as the trends in differences were consistent with the results obtained by window length of 30 time points (children, *r* = 0.79 ± 0.01; adolescents, *r* = 0.83 ± 0.02; adults, *r* = 0.76 ± 0.03; Figure S11A,B, Supporting Information). In addition, we used higher‐resolution Schaefer cortical segmentations of 800 ROIs to identify RFSI differences between the three stages of children, adolescents, and adults and similar results were obtained using Schaefer 400 ROIs (children, *r* = 0.84, *p*_spin_ < 0.001; adolescents, *r* = 0.86, *p*_spin_ < 0.001; adults, *r* = 0.79, *p*_spin_ < 0.001; Figure S11C, Supporting Information). Consistent with our primary results, children with ASD exhibited huge differences in the PFC and the differences diminished in adolescents with ASD, while adults with ASD showed the contrary pattern compared to that in children with ASD.

## 3 Discussion

In this study, we employed RFSI, genes expression profiles from AHBA dataset, neurotransmitters density maps, and single‐cell sequencing data to delineate the abnormal switching behaviors of functional connectivity dynamics and their underlying cellular and molecular basis in ASD. The following three main findings were obtained: First, aberrant switching behaviors in ASD were mainly associated with salience/ventral attention, default mode, and frontoparietal control networks and these aberrant switching behaviors in individuals with ASD exhibits age‐specific changes from childhood to adulthood. Second, the aberrant switching behaviors found in humans with ASD were similar to those found in ASD macaques and were spatially associated with the expression profiles of genes involved in cell morphogenesis; synaptic signaling; nervous system development; and neurotransmitters of mGluR5, 5‐HT4, and D1. Third, single‐cell sequencing of the PFC revealed that specific cell types, DEGs, and transcription factors regulate abnormal region‐wise FCD switching patterns in different age groups with ASD.

ASD is not a localized impairment in specific brain regions or systems, however, is viewed as a state stemming from an overall brain reorganization during early development.^[^ ^49^ ^]^ In comparison to typically developing children, individuals with ASD experience accelerated brain development in early life, leading to changes in connectivity.^[^ ^50^ ^]^ ASD is characterized by abnormal brain dynamics that manifest as increased variability in connectivity^[^ ^15^, ^16^, ^17^, ^18^ ^]^ and anomalous switches between connectivity states.^[^ ^19^, ^20^, ^22^, ^51^ ^]^ The switching across different states reflects effective integration between different functional systems and cognitive flexibility.^[^ ^52^ ^]^ Reports suggest that the abnormal instability of intermediate states in the ASD brain is associated with reduced indirect transitions and decreased flexibility in brain dynamics is related to the severity of ASD symptoms.^[^ ^53^ ^]^ However, region‐wise abnormal switching behaviors in ASD across different developmental stages remain unknown. Here, we employed a RFSI to characterize abnormalities in large‐scale brain dynamics in patients with ASD. Tracking the time‐varying amplitude of regional brain activity revealed the self‐organizing property of the brain during the resting state, demonstrating flexible switches between different functional states.^[^ ^9^, ^54^, ^55^, ^56^, ^57^ ^]^ The large‐scale functional network dynamic fluctuations manifested functionally from lower‐order unimodal to higher‐order transmodal processing systems.^[^ ^58^, ^59^ ^]^ A recent study reported two critical phenomena in human brain activity: the dynamic propagation of neural patterns and a functional hierarchical structure in large‐scale brain networks associated with complexity drops^[^ ^60^ ^]^ which is driven by the sensorimotor brain system^[^ ^12^ ^]^ and propagates to intrinsic higher‐order systems, displaying a predominant hierarchical structure.^[^ ^61^ ^]^ The adaptive functioning of the brain depends on the integration of information across time scales. Therefore, the diffusion of information to higher‐order cognitive systems over time leads to aberrant switching behaviors in individuals with ASD,^[^ ^62^, ^63^ ^]^ whereas the driving brain regions processing requires rapid adaptation to changing external stimuli and exhibits trivial variations.

In this study, individuals with ASD showed aberrant switching behaviors in higher‐order transmodal processing systems, such as the SN, DMN, and FPN, suggesting that these networks may play important roles in the neurophysiology and onset of ASD. Importantly, the SN, DMN, and FPN showed age‐specific changes in patients with ASD. The aberrant switching behaviors of the SN were found in all stages of ASD, from childhood to adolescence to adulthood, whereas the abnormal switching behaviors of the DMN and FPN in ASD were only observed in childhood and adolescence and in adolescence, respectively. The SN, with core areas of the anterior cingulate and ventral anterior insular, is involved in detecting and filtering salient stimuli and integrating sensory, emotional, and cognitive information for social behavior and self‐awareness processing.^[^ ^64^ ^]^ Although structural and functional impairments of the SN have been reported to be related to deficits in social processing in ASD, the developmental changes in the SN in ASD remain controversial.^[^ ^21^, ^65^ ^]^ In our study of the entire age spectrum of ASD, we observed that functional disruptions of the SN persisted from childhood to adulthood, suggesting long‐lasting social functional deficits in ASD. Furthermore, we observed no significant differences in FPN between children and adults, suggesting that early childhood may represent the initial stages of FPN development, with rapid developmental differences emerging during adolescence due to the surge in hormones during puberty, triggering rapid physical growth and significant influences on brain structure and function,^[^ ^66^ ^]^ causing developmental delay. However, this delay may be compensated for later. Recent research has shown a strong connection between loneliness and the DMN in the brain.^[^ ^67^ ^]^ The PCC and precuneus are parts of the DMN that have been implicated in combining bottom‐up attention with information from memory and perception.^[^ ^68^, ^69^ ^]^ We found aberrant switching behavior in the PCC, which is involved in RRB. In addition, abnormally delayed development of ASD in the DMN can be tracked through childhood, continued in adolescence, and compensated for in adulthood. This suggests that individuals with ASD may exhibit a weaker self‐concept or self‐perception from childhood than typically developing individuals do. This difference in self‐perception may have significant implications for understanding the experiences and challenges faced by individuals with ASD, highlighting the importance of tailored support and interventions to promote their well‐being and development.

Compared to human ASD, animal models of ASD display minor individual differences in mimicking clinical phenotypes.^[^ ^70^, ^71^, ^72^ ^]^ Owing to their higher genetic, physiological, and brain structures and functional similarities with humans compared to those of rodents and other animal models, macaques have been widely employed to investigate the neural basis of normal human brain cognitive functions and nervous system diseases.^[^ ^73^, ^74^, ^75^ ^]^ In our study, ASD macaques were used to validate findings observed in human ASD using fMRI. ASD macaques were generated using transcription activator‐like effector nuclease (TALEN)‐edited methyl‐CpG‐binding protein 2 (*MECP2*) mutant monkeys. ASD macaques exhibit clinical phenotypes similar to those found in humans with ASD, such as stereotypical and social behaviors.^[^ ^27^ ^]^ Recently, we revealed that ASD macaques exhibit reduced environmental exploration and conflict encounters while increasing sleep latency, as well as abnormal developmental dynamics of brain white matter microstructures and network topological organizations, which is similar to that found in human ASD.^[^ ^28^ ^]^ In this study, we analyzed RFSI in both humans and macaques with ASD and identified similar aberrant switching behaviors in brain regions, such as the left orbitofrontal PFCol, left Ip, right PCi, bilateral medial PMCm, anterior CCa, orbitomedial PFCom, and TCpol. These brain areas play crucial roles in social cognition, emotion regulation, silence, and sensory information processing, which are core functional deficits in patients with ASD.^[^ ^76^, ^77^, ^78^, ^79^, ^80^, ^81^ ^]^ Findings from both human and macaque ASD consistently revealed aberrant switching behaviors, which could help to better understand neuropathology and provide insights into precision therapy for ASD.

Findings at the cellular and molecular levels revealed strikingly similar differences to those observed in macroscopic imaging, with children exhibiting a greater number of DEGs in the PFC. In particular, we noted that downregulated DEGs were significantly more abundant in SST interneurons in children with ASD than those in adolescents and adults. SST and PVALB interneurons, which selectively interact with the dendrites of cortical projection neurons to modulate their inputs and outputs, respectively,^[^ ^82^ ^]^ have been shown to underlie the regional signaling differences across cortical layers.^[^ ^83^ ^]^ An increase in the relative density of SST interneurons may help filter out noisy or task‐irrelevant cortical signals and facilitate the recurrent excitation necessary for higher‐order cognition.^[^ ^84^, ^85^ ^]^ Although no significant changes in the relative density of SST interneurons were observed in our data, possibly due to sample size limitations, the enrichment of downregulated genes in the IN‐SST may indirectly support this notion. Additionally, we specifically identified the transcription factor MAF during childhood, which is enriched during the development of the medial ganglionic eminence, where it represses the generation of SST+ cortical and hippocampal interneurons.^[^ ^86^ ^]^ This suggests that in patients with childhood ASD, the regulatory role of MAF in SST interneuron development may be crucial for disease progression. This mechanism could also be a key reason why the severity of ASD is more pronounced in children than that in adolescents or adults. However, the disruption of interneuronal gene networks is not limited to the childhood stage. In the adult stage, we identified the transcription factor DLX2, which plays a crucial role in regulating the generation of GABAergic cortical interneurons. Notably, DLX2 is implicated in the susceptibility to ASD.^[^ ^87^ ^]^

Furthermore, in children with ASD, upregulated DEGs were significantly more abundant in the AST‐PP than that in other age groups. ASD profoundly affects cell differentiation across different developmental stages, particularly in astrocytes, which is consistent with previous findings.^[^ ^88^ ^]^ In typically developing children, intense neuroplasticity occurs during the first few years of life, coinciding with significant astrocyte proliferation and refinement.^[^ ^89^ ^]^ Astrocytes express GABA receptors, including ionic GABAA and metabolic GABAB receptors, as well as GABA transporters (GATs), such as GAT‐1 and GAT‐3.^[^ ^90^ ^]^ This suggests that astrocytes play a crucial role in the regulation of GABAergic neurons. In ASD, alterations in GABAergic inhibition affect the BOLD activity, further emphasizing the role of astrocytes in ASD‐related neural dysfunction.^[^ ^91^, ^92^ ^]^ In the pseudotime analysis of the AST‐PP, we found that the trajectory was not arranged according to the developmental stages of children and adolescents, rather mapped to the progression of ASD. This pattern was particularly evident in childhood, highlighting the developmental specificity of ASD in the AST‐PP during early childhood.

Neurotransmitter transport and synaptic signal transduction were found to be important for ASD at different developmental stages in our study, which is supported by previous findings on ASD.^[^ ^22^, ^93^, ^94^, ^95^, ^96^, ^97^ ^]^ DEGs related to synaptic signal transduction in childhood and adolescence showed a downregulation trend, while DEGs related to synaptic signal transduction and synaptic assembly in adulthood exhibited an upregulation trend, possibly related to the imbalance between excitatory and inhibitory metabolism in individuals with ASD.^[^ ^98^ ^]^ We also observed a significant downregulation of genes related to ATP synthesis in adulthood, suggesting a potential link with previous findings that ATP produced by astrocytes may regulate ASD‐like behavior through P2 × 2 receptors and possibly through GABAergic synaptic transmission.^[^ ^99^ ^]^ Through the above analyses, we speculate that GABAergic synaptic‐related genes in the PFC are significantly upregulated in adulthood and negatively regulate ATP generation.

The prevalence of ASD in males is 3.8 times higher than that in females, a gender disparity that has been consistently observed for a long time. In the child stage, ZFY, a transcription factor located on the Y chromosome that regulates hundreds of genes, may serve as a key regulator of sex differences in ASD.^[^ ^100^ ^]^ Additionally, CLOCK, a transcription factor associated with circadian rhythm regulation, has been strongly linked to ASD, suggesting that sleep disturbances in patients with ASD may stem from molecular deficits rather than pure behavioral manifestations.^[^ ^101^ ^]^ These transcription factors were highly and specifically enriched only during childhood. In adolescents, ESRRG, a member of the estrogen receptor family, plays a notable role in steroid level regulation.^[^ ^102^ ^]^ Moreover, the PBX1 transcription factor has been implicated in ASD, further supporting its potential involvement in the pathophysiology of ASD.^[^ ^103^ ^]^

Our study had several limitations. The first is the choice of the sliding window; although we used different windows to validate our findings on the images, the choice of window was not determined by a suitable technique to achieve optimal results. Furthermore, in single‐cell analysis, the small sample size (N = 1 or 2 per age group) reduces the statistical power and may affect the reproducibility and generalizability of the findings. Although integrating external datasets can enhance the robustness, batch effects from different sequencing platforms and processing methods remain a challenge. Future studies with larger cohorts are required to validate these findings.

## 4 Conclusion

In summary, charactering the aberrant switching behaviors of brain for ASD from childhood to adulthood not only provides new fundamental insights into the origins, developmental processes, and potential influencing factors of abnormal dynamics in ASD, but also offers novel perspectives on molecular and neurotransmitter mechanisms underlying functional connectivity dynamic switching behaviors abnormalities associated with ASD by integrating functional MRI, transcriptomic, and neurotransmitter data. Importantly, single‐cell sequencing was used to identify the DEGs and cell types associated with macroscopic neuroimaging phenotypic changes. The prefrontal cortical regions were found to exhibit heterogeneous RFSI across different ages, potentially due to the disruption of gene networks in SST interneurons and altered differentiation patterns in the astrocyte State2. We also found that dysregulation of the MAF transcription factor in childhood and the DLX2 transcription factor in adulthood may be key factors contributing to the disruption of interneuron gene networks. Our results provide primary evidence of aberrant switching behaviors and the associated cellular and molecular bases of ASD, which may facilitate better diagnosis and new treatment strategies for ASD.

## 5 Experimental Section

### Human Subjects and MRI Data Acquisition

In our study, a public dataset from the ABIDE was used to reveal aberrant switching patterns in whole‐brain functional connectivity dynamics (FCD) in developmental ASD.^[^ ^104^, ^105^ ^]^ The study protocols and MRI scans of the participants in the ABIDE dataset were approved by the local Ethics Committee. Details of the MRI scanners, magnetic strength, repetition time, echo time, voxel‐wise resolution, and fMRI measurements are shown in Table S1, Supporting Information. After quantity control, 1700 subjects (820 ASD and 880 typical controls) were used for further analyses (Table S2, Supporting Information).

### Human Structural MRI Data Preprocessing

The T1‐weighted (T1w) images was initially corrected for intensity non‐uniformity using the *N4BiasFieldCorrection* function.^[^ ^106^ ^]^ Subsequently, the T1w image was skull‐stripped using a Nipype implementation of the antsBrainExtraction workflow (from ANTs), employing OASIS30ANTs as the target template. Brain tissue segmentation of the cerebrospinal fluid (CSF), white matter (WM), and gray matter was performed using FAST in the FSL toolkit.^[^ ^107^ ^]^ A T1w reference map was created using the *mri_robust_template* function (FreeSurfer 7.2.0).^[^ ^108^ ^]^ Brain surfaces were reconstructed using recon‐all^[^ ^109^ ^]^ and the previously estimated brain mask was refined with a custom variation of the method to reconcile ANTs‐derived and FreeSurfer‐derived segmentations of the cortical gray matter of Mindboggle.^[^ ^110^ ^]^ Volume‐based spatial normalization to two standard spaces (MNI152NLin6Asym, MNI152NLin2009cAsym) was performed through nonlinear registration using antsRegistration (ANTs 2.3.3). The templates selected for spatial normalization were FSL's MNI ICBM 152 nonlinear sixth Generation Asymmetric Average Brain Stereotaxic Registration model and^[^ ^111^ ^]^ ICBM 152 Nonlinear Asymmetrical template version 2009c.^[^ ^112^ ^]^

### Human fMRI Data Preprocessing

The human fMRI data were first head‐motion‐corrected using FSL's mcflirt^[^ ^113^ ^]^ and slice time‐corrected using AFNI's 3dTshift from AFNI.^[^ ^114^ ^]^ Subsequently, the fMRI data were coregistered to the T1w reference using bbregister (FreeSurfer), which implements a boundary‐based registration^[^ ^115^ ^]^ with six degrees of freedom. Several confounding time series were calculated based on the preprocessed BOLD data: frame‐wise displacement (FD), DVARS, and three region‐wise global signals. In addition, a set of physiological regressors was extracted to allow component‐based noise correction (CompCor).^[^ ^116^ ^]^ The BOLD time series were resampled into a standard space, generating a preprocessed BOLD run in the MNI152NLin6Asym space. First, a reference volume and its skull‐stripped version were generated using the custom fMRIPrep methodology. Automatic removal of motion artifacts using independent component analysis was performed on the preprocessed BOLD on the MNI space time series after removal of non‐steady‐state volumes and spatial smoothing with an isotropic,^[^ ^117^ ^]^ Gaussian kernel of 6 mm FWHM (full‐width half‐maximum FWHM). All resampling steps were performed with a single interpolation step by composing all pertinent transformations (i.e., head‐motion transform matrices, susceptibility distortion correction when available, and co‐registration with anatomical and output spaces). Confounders, including white matter and cerebrospinal fluid signals, were computed and regressed from the preprocessed fMRI data for each individual. The residuals of this regression were then subjected to a high‐pass filtering of 0–0.01 Hz.

### ASD Macaques, fMRI Data Acquisition, and Preprocessing

To validate the findings of aberrant switching patterns of human FCD in patients with ASD, we investigated cortical FCD in macaques with ASD. We utilized a total of nine female cynomolgus monkeys including five ASD monkeys (age = 8.8 ± 0.84) and four WT monkeys (age = 9 ± 0.82), from the colonies at the State Key Laboratory of Primate Biomedical Research, Institute of Primate Translational Medicine, Kunming University of Science and Technology were used in this study. ASD monkeys were generated using TALEN‐based *MECP2* mutations and demonstrated behavioral phenotypes similar to those of human ASD in our previous studies.^[^ ^27^, ^28^ ^]^ Experimental procedures adhered to the guidelines and regulations of the National Care and Use of Animals and were approved by the National Animal Research Authority of China and the Institutional Animal Care and Committee of the State Key Laboratory of Primate Biomedical Research, Institute of Primate Translational Medicine, Kunming University of Science and Technology (permit number: KUST202301017).

Resting‐state fMRI data were acquired using a 3.0 T Siemens Prisma MRI scanner equipped with a specific monkey coil. All the monkeys were under anesthesia during MRI acquisition and were scanned using a standardized echo planar imaging sequence with the following parameters: repetition time (TR) = 2000 ms, echo time (TE) = 22 ms, flip angle = 90°, matrix size = 64 × 64, voxel size = 1.5 × 1.5 × 1.5 mm^3^, 250 volumes.

Resting‐state fMRI data of ASD monkeys underwent preprocessing with the following steps. The first 10 volumes were discarded to promote magnetization equilibrium and realigned to the first volume to correct for head motion. All fMRI images were normalized to a standard template with dimensions of 1 mm × 1 × 1 mm^3^. Smoothing was performed using a Gaussian kernel with a full width of 3 mm at half‐maximum. The fMRI data were detrended to exclude linear bias. Regressions were performed on the Firston‐24 head motion parameters, mean WM, and CSF signals. Temporal bandpass filtering of 0–0.05 Hz was also applied. To exclude the effect of head motion, data with the head motion translated or rotated by more than one voxel in any direction were removed. None of the participants were excluded based on this criterion. Scrubbing was used to exclude poor images (two time points before and one time point after) with excessive motion beyond the preset criterion (frame displacement: FD, FD < 0.2). No global signal regression was performed to avoid false discovery of negatively correlated regions in the brain, ensuring the reliability of the results obtained.^[^ ^118^ ^]^

### Region‐Wise FCD Switching Index

To identify aberrant switching patterns of whole‐brain FCD in patients with ASD, RFSI was calculated as follows: First, the entire cortex was subdivided into 400 regions of interest (ROIs) using the Schaefer cortical atlas.^[^ ^119^ ^]^ Subsequently, the average time series within each ROI was calculated, generating a 400 × *t* time series matrix (where *t* represents the time points of the fMRI data) for each participant. The FCD was computed using the sliding window method with the window length set at *w* = 30 time points and a step of one time point (a total of *t* – *w* + 1 windows). In each window, the ROI‐wise functional connectivity was calculated using Pearson's correlation coefficient to yield a 400 × 400 matrix. The functional connectivity matrix in each window was vectorized and Pearson's correlations between the vectorized functional connectivity matrices in different windows were calculated to form a (*t* – *w* + 1) × (*t* – *w* + 1) FCD matrix for each subject. The FCD matrix was averaged to form a (*t* – *w* + 1) vector representing the whole‐brain mean FCD for each participant. Next, the SWSTD of time series within each window was calculated for each ROI, resulting in a 400 × (*t* – *w* + 1) SWSTD matrix for each subject. The switching behavior of the cortical regions was captured by calculating the Spearman correlation between the first‐order derivatives of the FCD‐mean and SWSTD.^[^ ^12^ ^]^ The RFSI, which describes region‐wise switching behaviors, was used as the metric. To validate the results obtained with a window length of *w* = 30, the RFSI at different window lengths (*w* = 24 and 36) were calculated (validation analysis section).

### Harmonization of Multi‐Site RFSI

To determine RFSI differences between individuals with ASD and TC, we initially corrected individual RFSI biases due to variations in sites, scanning machines, and parameters, and different time measurements using the ComBat harmonization algorithm before statistical analyses.^[^ ^120^ ^]^ The *z*‐score map was generated for each individual after multisite harmonization.

ComBat can be described as follows: Assuming that the data are collected from *m* different sites, where i=1,2,….

(1) $$y_{ijv}=a_{v}+X_{i,j}\beta_{v}+\gamma_{i,v}+\delta_{i,v}\varepsilon_{i,j,v}$$

In Equation (1), *j* represents the individual sample index and *a_v_* denotes the RFSI in region *v*. *X* is the design matrix of the covariates of interest (e.g., gender and age). β_*v*_ is the region‐specific vector of regression coefficients corresponding to *X*. The terms γ_*i*,*v*_ and δ_*i*,*v*_ denote the additive and multiplicative site effects at site *i* of region *v*.

### Abnormal RFSI in ASD

To identify abnormal RFSI in developmental ASD, 1700 participants were divided into three age groups: children (6–12 years old, ASD: *n* = 253, TC: *n* = 345), adolescents (12–18 years old, ASD: *n* = 293, TC: *n* = 252), and adults (18 years and older, ASD: *n* = 274, TC: *n* = 283). Subsequently, we conducted MANOVA to investigate the aberrant switching behaviors of individuals with ASD across different developmental stages. Factors for MANOVA included group (ASD and TC) and age (children, adolescents, and adults). The significance level for MANOVA was set at *p* < 0.05 corrected using the FDR method.

### Differences of RFSI in Severe and Mild ASD

Further exploration of the RFSI differences between severe and mild ASD, as defined by clinical symptoms, was conducted. Participants with ASD were first categorized into severe and mild ASD groups based on scores above and below the average on the Autism Diagnostic Interview (ADI) and Autism Diagnostic Observation Schedule (ADOS) (Table S3, Supporting Information). Unpaired two‐sample *t*‐tests were used to compare the RFSI in different brain regions between patients with severe and mild ASD. A significant difference in RFSI was observed between the two groups in a particular brain region, indicating that regional RFSI was associated with clinical phenotypes. The regional RFSI differences were corrected using FDR with *p* < 0.05.

### Functional Decoding for Brain Areas with RFSI Differences in ASD

To understand the cognitive functions of brain areas showing abnormalities in RFSI, functional decoding for these brain areas was conducted using Neurosynth meta‐analysis. The statistical *F*‐map (group effects) obtained by MANOVA was sorted with *F*‐values and 20 binary masks were generated as the threshold values increased by 5% each time. Using the 24 topic terms selected by Margulies et al,^[^ ^121^ ^]^ meta‐analysis based functional decoding was performed for each mask to assess the topic terms related to disease factors. For each region of interest map, the analysis output was a *z*‐statistic associated with the feature term. Terms with *z*‐statistic > 3.1 (corresponding to *p* < 0.05, FDR corrected) were considered significant associations.

### Spatial Correlation Analysis with Neurotransmitter Density Maps

To reveal abnormal RFSI‐associated neurotransmitters, spatial correlations between neurotransmitter density maps and group effects were performed to identify ASD‐related neurotransmitters. A total of 37 neurotransmitter receptors obtained from the published literature and provided by the Neuromaps toolbox were tested in our study (Table S4, Supporting Information).^[^ ^122^ ^]^ First, an *F*‐map based on MANOVA (group effects; Figure 2A) and neurotransmitter density maps were converted from the MNI‐152 voxel space to the fslR32K surface space. Next, we correlated the transformed *F*‐map with each of the neurotransmitter density maps (Pearson's *r*) and significance was tested using a null model that preserves spatial autocorrelation (spin‐test, randomly “rotates” Group effects *F*‐map 1000 times to account for spatial correlations),^[^ ^123^ ^]^ with a significance level of *p* < 0.05.

### Association between Alterations in RFSI and Gene Expression Profiles

Human gene expression data derived from six autopsy donors (five males and one female) from the Allen Institute,^[^ ^124^ ^]^ aged 24–57 years (42.5 ± 13.4), are available in the AHBA dataset (http://human.brain‐map.org). The AHBA microarray expression data were preprocessed and integrated into a matrix of 400 ROIs × 15 633 genes.^[^ ^124^, ^125^, ^126^ ^]^ Due to under‐sampling in the right hemisphere, only the gene expression data in the left hemisphere were analyzed. After removing ROIs with null values from the expression matrix, a 192 × 15 633 gene expression matrix (192 regions, 15 633 genes) was used for the analysis.

In our study, PLS regression was employed to link gene expression profiles to RFSI differences generated by MANOVA (*F*‐map for Group effects) to reveal the molecular basis. The first two components of the PLS regression (PLS1 and PLS2) explained ≈30% of the variance in the Group effect *F*‐map, with the first principal component (PLS1), a linear combination of gene expression values, showing the highest correlation with the regions of between‐group variation. To account for spatial autocorrelation, the null hypothesis that PLS1 or PLS2 explained no more covariance between the Group effect *F*‐map and whole‐genome expression than would be expected by chance was tested using a permutation test based on a spin test (10 000 times). Finally, bootstrapping was used to estimate the degree of PLS1 variation for each gene and *z*‐scores were calculated to rank the genes according to their contributions to PLS1.^[^ ^127^, ^128^ ^]^ A set of positively associated genes corrected with *p* < 0.05 using the FDR method were selected for gene enrichment analysis using Metascape to identify significant biological processes.^[^ ^129^ ^]^

### Single‐Nucleus RNA Sequencing of Prefrontal Cortex for ASD

From the statistical *F*‐map (group × age effects), significant differences in the RFSI of the PFC were identified. To further determine the cellular and molecular basis of RFSI differences in the PFC, public snRNA‐seq data for the PFC from five typical controls and five patients with ASD were accessed (https://www.ebi.ac.uk/ena/browser/view/PRJNA434002). We matched the data samples to age groups and categorized them into child (ASD = 1, TC = 1), adolescent (ASD = 2, TC = 2), and adult (ASD = 2, TC = 2) groups (Table S5, Supporting Information). Notably, the snRNA‐seq dataset also included data from other brain regions within the PFC. However, these samples were excluded to avoid anatomical bias. The analysis primarily focused on snRNA‐seq data from regions outside of BA9 and BA46.^[^ ^130^ ^]^

### 10X Genomic Data Preprocessing and Annotation

First, for the fastq data accessed from the EMBL‐EBI database, Cellranger software (v. 7.1.0) was used to perform alignment,^[^ ^131^ ^]^ filtering, barcode counting, UMI counting, and alignment to the GRCh38 human genome reference and annotation. For quality control, we filtered the count expression matrix obtained using CellRanger, retaining nuclei with genes (nFeature_RNA) and UMIs (nCount_RNA) within one standard deviation of their means. Additionally, we retained nuclei in which the expression of mitochondrial and ribosomal genes accounted for less than 5% of total gene expression. DecontX (v. 1.2.0) and DoubletFinder (v. 2.0.4) were used to remove potential RNA contaminants and doublet cells, respectively.^[^ ^132^, ^133^ ^]^ We then clustered nuclei for further analysis. The count expression matrix was merged and analyzed using Seurat (v 5.1.0).^[^ ^134^ ^]^ To mitigate the impact of varying cell sequencing depths, gene read counts were normalized based on equalized library sizes and standardized accordingly. Subsequently, singular value decomposition with k = 50 was performed. The JackStraw algorithm and elbow plot visualization were employed to identify and select the top 16 principal components deemed significant. These PCs were utilized in the K‐nearest neighbors algorithm to identify neighboring points and reduce the dimensionality of batch‐corrected data. Subsequently, the Shared Nearest Neighbor algorithm was applied to cluster the reduced dimensions to identify and delineate clusters representing similar cell or gene expression patterns. To achieve visualization in 2D space, Uniform Manifold Approximation and Projection embedding was rendered. To delineate the distinct cell types, we annotated the aggregation of 16 clusters based on marker genes provided in the literature.^[^ ^130^ ^]^ Notably, fibrous astrocytes, predominantly found in the white matter of the brain, were observed exclusively in one subject. Given the primary focus of this study on cell types within the cerebral cortex, this particular cell type was excluded from subsequent investigations.

### DEGs Selection and GO Enrichment Analysis

The Wilcoxon rank‐sum test was conducted to identify DEGs between child, adolescent, and adult patients across different cell types compared to the corresponding typical controls. Genes exhibiting a corrected *p*‐value less than 0.05 were considered significantly differentially expressed in each cross‐sectional comparison (Bonferroni correction). Subsequently, GO enrichment for DEGs was performed to identify the main molecular function using the clusterProfiler package in R.^[^ ^135^ ^]^

### Cell Type Analysis of the DEGs using AUCell

The AUCell method was employed to identify the enrichment of DEGs in specific or multiple cell types.^[^ ^136^ ^]^ First, the DEGs were ranked by sorting the genes from highest to lowest expression values for each cell. Subsequently, the enrichment of the gene signatures was computed by assessing the area under the curve (AUC) of the recovery curve to ascertain the extent to which a gene set was enriched at the top of the gene ranked for each cell. The AUC provides an estimate of the proportion of genes within the gene set that exhibit increased expression in each cell. Cells expressing more genes from a gene set were characterized by higher AUC values than those expressing fewer genes. To calculate the AUC, only the top 10% of ranked genes were considered. The gene sets used for the different age groups were composed of DEGs between ASD and TC across age groups without classification into distinct cell types. This gene set was further subdivided into upregulated and downregulated genes. Finally, AUCell was employed to map these gene subsets onto cell types within different age groups to facilitate the computation of the percent and average expression metrics.

### Transcription Factors Analysis

To identify differences in TFs, a single‐cell gene expression matrix was analyzed for regulon activity using the pySCENIC pipeline (v 0.12.1).^[^ ^136^ ^]^ The workflow consisted of three steps: (1) Construction of co‐expression networks using GENIE3. Using the single‐cell gene expression matrix as the input file and considering each target gene as the output, a random forest tree was constructed for each gene. Importance metrics were computed for each TF‐gene pair based on the constructed random forest trees. Ultimately, this process yielded co‐expression modules for TF‐gene pair; (2) Motif enrichment and target gene prediction using RcisTarget. Using the gene‐motif ranking database, each motif was accumulated across all genes within a module. The larger the AUC of the gene accumulation curve in the module, the higher the enrichment level of the motif within that module. For each module, significant motif enrichment was selected and their target genes were predicted. Ultimately, a gene regulatory network module (regulon) encompassing both TFs and their target genes was formed by integrating TF‐gene modules and the results of target gene predictions; (3) Quantification of regulon activity using AUCell. Within SCENIC, gene sets refer to all genes within the regulons. For each cell line, the genes were ranked in descending order based on their expression levels. Using the positions of the genes within the regions along the ranked sequence, the cumulative AUC value was computed. AUC represents the regulon activity score (RAS) of the cell. Regulon specificity scores were computed in accordance with the cell clusters identified by Seurat and the top regulons for each cell cluster were selected following the SCENIC protocol implemented in Python.

### Pseudotime Analysis

Pseudotime analysis of the astrocyte cluster derived from cell clustering was executed using Monocle2 (v 2.32.0).^[^ ^137^ ^]^ Initially, a Seurat object exclusively containing astrocyte subtypes was employed to construct the CellDataSet object for preprocessing. Subsequently, the detectGenes function was applied to assess the number of cells expressing specific genes to facilitate the trajectory analysis of single‐cell differentiation. Finally, the dpFeature method was selected to replace the highly variable genes or DEGs identified by Seurat within the subtypes to identify the genes governing cellular progression; reduced the data to two dimensions and arranged cells using the orderCells function.

### Statistical Analysis

Statistical analyses were performed using SPSS, Python, and MATLAB software. To better understand how brain developmental changes affect ASD, this study applied *Combat* corrections to individual RFSI values before analysis to eliminate biases caused by differences in acquisition sites, scanning devices and parameters, as well as measurements taken at different times. Age and sex were included as covariates for adjustment and individual data were standardized (*z*‐scores). Individuals were divided into three groups based on age: children, adolescents, and adults. A 2 × 3 two‐way MANOVA model was constructed for each ROIs and MANOVA was performed using SPSS software. Benjamini‐Hochberg FDR correction was applied to identify significantly different brain regions (Figure 2A; Table S6, Supporting Information). The behavioral scores of the ASD group were divided into two categories based on whether they were above or below the mean (Figure 2B; Figure S2B,C, Supporting Information). Unpaired two‐sample *t*‐tests were conducted to compare the differences in RFSI between the two groups in specific brain regions and *p*‐values were corrected using Benjamini‐Hochberg FDR. Unpaired two‐sample *t*‐tests were performed to test the intergroup differences between ASD and TC within each network for each age group and the Bonferroni correction was applied (Figure 2C; Table S7, Supporting Information). Spin tests and Pearson's correlation analyses were performed to evaluate the correlation between RFSI changes and neurotransmitter levels. The *F*‐map for each group was projected onto the fslr32k surface space to create a surface‐based segmentation representation. Using a spherical projection of the mean surface, the spatial coordinates for each parcel were defined by selecting the vertex closest to the centroid of each parcel. These parcel coordinates were then randomly rotated and the original parcel values were reassigned to the closest rotated parcels (repeated 10 000 times). In addition, unpaired two‐sample *t*‐tests were conducted to assess intergroup differences between ASD and TC across different age groups (Figure S3, Supporting Information) and the same method was used to compare intergroup differences in the monkeys (Figure 4). Spin tests were employed to evaluate the correlation between the Group *F*‐map and PLS1 and PLS2 scores (repeated 10 000 times) (Figure S5, Supporting Information). Bootstrapping (repeated 10 000 times) was used to estimate the variance of each gene in PLS1 and z‐scores were calculated. Differential expression between the ASD and TC groups was calculated using the Wilcoxon rank‐sum test. Correction was performed using Bonferroni correction. For all statistical analyses, the significance level was set at *p* < 0.05. All values in the text and figure legends are represented as the mean ± SD.

## Conflict of Interest

The authors declare no conflict of interest.

## Supporting information



Supporting Information

## Data Availability

The data that support the findings of this study are available from the corresponding author upon reasonable request.;

## References

[1] K. J. Friston , Brain Connect. 2011, 1, 13. 22432952 10.1089/brain.2011.0008

[2] R. F. Betzel , Network Neurosci. 2020, 4, 234. 10.1162/netn_a_00121 PMC7055648 32166210

[3] A. Gozzi , A. J. Schwarz , NeuroImage 2016, 127, 496. 26706448 10.1016/j.neuroimage.2015.12.017

[4] Y. Ma , M. A. Shaik , M. G. Kozberg , S. H. Kim , J. P. Portes , D. Timerman , E. M. C. Hillman , Proc. Natl. Acad. Sci. U.S.A. 2016, 113, E8463. 27974609 10.1073/pnas.1525369113 PMC5206542

[5] R. M. Hutchison , T. Womelsdorf , J. S. Gati , S. Everling , R. S. Menon , Hum. Brain Mapp. 2013, 34, 2154. 22438275 10.1002/hbm.22058 PMC6870538

[6] R. Liégeois , J. Li , R. Kong , C. Orban , D. Van De Ville , T. Ge , M. R. Sabuncu , B. T. Yeo , Nat. Commun. 2019, 10, 2317. 31127095 10.1038/s41467-019-10317-7 PMC6534566

[7] D. J. Lurie , D. Kessler , D. S. Bassett , R. F. Betzel , M. Breakspear , S. Kheilholz , A. Kucyi , R. Liégeois , M. A. Lindquist , A. R. McIntosh , Network Neurosci. 2020, 4, 30. 10.1162/netn_a_00116 PMC7006871 32043043

[8] D. Vidaurre , S. M. Smith , M. W. Woolrich , Proc. Natl. Acad. Sci. U.S.A. 2017, 114, 12827. 29087305 10.1073/pnas.1705120114 PMC5715736

[9] A. Zalesky , A. Fornito , L. Cocchi , L. L. Gollo , M. Breakspear , Proc. Natl. Acad. Sci. U.S.A. 2014, 111, 10341. 24982140 10.1073/pnas.1400181111 PMC4104861

[10] G. Deco , M. L. Kringelbach , V. K. Jirsa , P. Ritter , Sci. Rep. 2017, 7, 3095. 28596608 10.1038/s41598-017-03073-5 PMC5465179

[11] E. C. Hansen , D. Battaglia , A. Spiegler , G. Deco , V. K. Jirsa , NeuroImage 2015, 105, 525. 25462790 10.1016/j.neuroimage.2014.11.001

[12] X. Kong , R. Kong , C. Orban , P. Wang , S. Zhang , K. Anderson , A. Holmes , J. D. Murray , G. Deco , M. van den Heuvel , Nat. Commun. 2021, 12, 6373. 34737302 10.1038/s41467-021-26704-y PMC8568904

[13] B. S. Abrahams , D. H. Geschwind , Nat. Rev. Genet. 2008, 9, 341. 18414403 10.1038/nrg2346 PMC2756414

[14] D. American Psychiatric Association, A. P. Association , Diagnostic and statistical manual of mental disorders: DSM‐5, Vol. 5, American Psychiatric Association, Washington, DC 2013.

[15] H. Chen , J. S. Nomi , L. Q. Uddin , X. Duan , H. Chen , Hum. Brain Mapp. 2017, 38, 5740. 28792117 10.1002/hbm.23764 PMC5783325

[16] M. Falahpour , W. K. Thompson , A. E. Abbott , A. Jahedi , M. E. Mulvey , M. Datko , T. T. Liu , R.‐A. Müller , Brain Connect. 2016, 6, 403. 26973154 10.1089/brain.2015.0389 PMC4913487

[17] V. Harlalka , R. S. Bapi , P. K. Vinod , D. Roy , Front. Hum. Neurosci. 2019, 13, 6. 30774589 10.3389/fnhum.2019.00006 PMC6367662

[18] Y. Li , Y. Zhu , B. A. Nguchu , Y. Wang , H. Wang , B. Qiu , X. Wang , Autism Res. 2020, 13, 230. 31614075 10.1002/aur.2212

[19] N. De Lacy , D. Doherty , B. H. King , S. Rachakonda , V. D. Calhoun , NeuroImage: Clin. 2017, 15, 513. 28652966 10.1016/j.nicl.2017.05.024 PMC5473646

[20] Z. Fu , Y. Tu , X. Di , Y. Du , J. Sui , B. B. Biswal , Z. Zhang , N. de Lacy , V. D. Calhoun , NeuroImage 2019, 190, 191. 29883735 10.1016/j.neuroimage.2018.06.003 PMC6281849

[21] L. Q. Uddin , V. Menon , Neurosci. Biobehav. Rev. 2009, 33, 1198. 19538989 10.1016/j.neubiorev.2009.06.002 PMC2743776

[22] Y. Xie , Z. Xu , M. Xia , J. Liu , X. Shou , Z. Cui , X. Liao , Y. He , Biol. Psychiatry 2022, 91, 945. 35144804 10.1016/j.biopsych.2021.12.004

[23] E. J. Vallender , C. E. Hotchkiss , A. D. Lewis , J. Rogers , J. A. Stern , S. M. Peterson , B. Ferguson , K. Sayers , Orphanet Journal of. Rare Dis. 2023, 18, 20. 10.1186/s13023-023-02619-3 PMC9887761 36721163

[24] A. S. Fox , R. A. Harris , L. D. Rosso , M. Raveendran , S. Kamboj , E. L. Kinnally , J. P. Capitanio , J. Rogers , Mol. Psychiatry 2021, 26, 6609. 34035480 10.1038/s41380-021-01156-4 PMC8613309

[25] J. Rogers , S. E. Shelton , W. Shelledy , R. Garcia , N. H. Kalin , Genes, Brain Behav. 2008, 7, 463. 18045243 10.1111/j.1601-183X.2007.00381.x PMC2785008

[26] D. E. Williamson , K. Coleman , S.‐A. Bacanu , B. J. Devlin , J. Rogers , N. D. Ryan , J. L. Cameron , Biol. Psychiatry 2003, 53, 284. 12586447 10.1016/s0006-3223(02)01601-3

[27] Y. Chen , J. Yu , Y. Niu , D. Qin , H. Liu , G. Li , Y. Hu , J. Wang , Y. Lu , Y. Kang , Cell 2017, 169, 945. 28525759 10.1016/j.cell.2017.04.035 PMC5540256

[28] J. Wang , Z. Wang , H. Zhang , S. Feng , Y. Lu , S. Wang , H. Wang , Y. E. Sun , Y. Chen , Cereb. Cortex 2021, 31, 5396. 34117744 10.1093/cercor/bhab166

[29] S. Kanthaswamy , R. Reader , R. Tarara , K. Oslund , M. Allen , J. Ng , C. Grinberg , D. Hyde , D. G. Glenn , N. Lerche , J. Med. Primatol. 2014, 43, 288. 25422529 10.1111/jmp.12127 PMC4240635

[30] J. R. Reader , D. R. Canfield , J. F. Lane , S. Kanthaswamy , A. Ardeshir , A. M. Allen , R. P. Tarara , Comp. Med. 2016, 66, 162. 27053572 PMC4825967

[31] G. Cai , S. A. Cole , M. E. Tejero , J. M. Proffitt , J. H. Freeland‐Graves , J. Blangero , A. G. Comuzzie , Obes. Res. 2004, 12, 1766. 15601971 10.1038/oby.2004.219

[32] B. K. Dray , M. Raveendran , R. A. Harris , F. Benavides , S. B. Gray , C. J. Perez , M. J. McArthur , L. E. Williams , W. B. Baze , H. Doddapaneni , D. M. Muzny , C. R. Abee , J. Rogers , Genes Cancer 2018, 9, 142. 30108684 10.18632/genesandcancer.170 PMC6086002

[33] H. A. Simmons , J. A. Mattison , Antioxid. Redox Signaling 2011, 14, 221. 10.1089/ars.2010.3311 PMC3014768 20524847

[34] A. Fornito , A. Arnatkevičiūtė , B. D. Fulcher , Trends Cognit. Sci. 2019, 23, 34. 30455082 10.1016/j.tics.2018.10.005

[35] V. Beliveau , M. Ganz , L. Feng , B. Ozenne , L. Højgaard , P. M. Fisher , C. Svarer , D. N. Greve , G. M. Knudsen , J. Neurosci. 2017, 37, 120. 28053035 10.1523/JNEUROSCI.2830-16.2016 PMC5214625

[36] J. Y. Hansen , G. Shafiei , R. D. Markello , K. Smart , S. M. L. Cox , M. Nørgaard , V. Beliveau , Y. Wu , J.‐D. Gallezot , É. Aumont , S. Servaes , S. G. Scala , J. M. DuBois , G. Wainstein , G. Bezgin , T. Funck , T. W. Schmitz , R. N. Spreng , M. Galovic , M. J. Koepp , J. S. Duncan , J. P. Coles , T. D. Fryer , F. I. Aigbirhio , C. J. McGinnity , A. Hammers , J.‐P. Soucy , S. Baillet , S. Guimond , J. Hietala , et al., Nat. Neurosci. 2022, 25, 1569. 36303070 10.1038/s41593-022-01186-3 PMC9630096

[37] M. Nørgaard , V. Beliveau , M. Ganz , C. Svarer , L. H. Pinborg , S. H. Keller , P. S. Jensen , D. N. Greve , G. M. Knudsen , NeuroImage 2021, 232, 117878. 33610745 10.1016/j.neuroimage.2021.117878 PMC8256681

[38] K. Zilles , N. Palomero‐Gallagher , Front. Neuroanat. 2017, 11, 78. 28970785 10.3389/fnana.2017.00078 PMC5609104

[39] S. R. Krishnaswami , R. V. Grindberg , M. Novotny , P. Venepally , B. Lacar , K. Bhutani , S. B. Linker , S. Pham , J. A. Erwin , J. A. Miller , R. Hodge , J. K. McCarthy , M. Kelder , J. McCorrison , B. D. Aevermann , F. D. Fuertes , R. H. Scheuermann , J. Lee , E. S. Lein , N. Schork , M. J. McConnell , F. H. Gage , R. S. Lasken , Nat. Protoc. 2016, 11, 499. 26890679 10.1038/nprot.2016.015 PMC4941947

[40] S. Scidraw , Human Brain 2020.

[41] S. Scidraw , human brain silhouette 2020.

[42] M. S. Breault , Human Brain 2020.

[43] M. S. Breault , Monkey Brain 2020.

[44] P. Kraemer , synapse 2020.

[45] X. Kong , R. Kong , C. Orban , P. Wang , S. Zhang , K. Anderson , A. Holmes , J. D. Murray , G. Deco , M. Van Den Heuvel , B. T. T. Yeo , Nat. Commun. 2021, 12, 6373. 34737302 10.1038/s41467-021-26704-y PMC8568904

[46] B. T. T. Yeo , F. M. Krienen , J. Sepulcre , M. R. Sabuncu , D. Lashkari , M. Hollinshead , J. L. Roffman , J. W. Smoller , L. Zöllei , J. R. Polimeni , B. Fischl , H. Liu , R. L. Buckner , J. Neurophysiol. 2011, 106, 1125. 21653723 10.1152/jn.00338.2011 PMC3174820

[47] S. Aibar , C. B. González‐Blas , T. Moerman , V. A. Huynh‐Thu , H. Imrichova , G. Hulselmans , F. Rambow , J.‐C. Marine , P. Geurts , J. Aerts , J. van den Oord , Z. K. Atak , J. Wouters , S. Aerts , Nat. Methods 2017, 14, 1083. 28991892 10.1038/nmeth.4463 PMC5937676

[48] M. Linnerbauer , M. A. Wheeler , F. J. Quintana , Neuron 2020, 108, 608. 32898475 10.1016/j.neuron.2020.08.012 PMC7704785

[49] C. Lord , M. Elsabbagh , G. Baird , J. Veenstra‐Vanderweele , Lancet 2018, 392, 508. 30078460 10.1016/S0140-6736(18)31129-2 PMC7398158

[50] J. D. Lewis , A. C. Evans , J. R. Pruett , K. Botteron , L. Zwaigenbaum , A. Estes , G. Gerig , L. Collins , P. Kostopoulos , R. McKinstry , Transl. Psychiatr. 2014, 4, 388. 10.1038/tp.2014.24 PMC4035719 24802306

[51] L. Q. Uddin , B. T. T. Yeo , R. N. Spreng , Brain Topogr 2019, 32, 926. 31707621 10.1007/s10548-019-00744-6 PMC7325607

[52] X. Liao , M. Cao , M. Xia , Y. He , NeuroImage 2017, 152, 94. 28242315 10.1016/j.neuroimage.2017.02.066

[53] T. Watanabe , G. Rees , Nat. Commun. 2017, 8, 16048. 28677689 10.1038/ncomms16048 PMC5504272

[54] E. A. Allen , E. Damaraju , S. M. Plis , E. B. Erhardt , T. Eichele , V. D. Calhoun , Cereb. Cortex 2014, 24, 663. 23146964 10.1093/cercor/bhs352 PMC3920766

[55] V. D. Calhoun , R. Miller , G. Pearlson , T. Adalı , Neuron 2014, 84, 262. 25374354 10.1016/j.neuron.2014.10.015 PMC4372723

[56] C. Chang , G. H. Glover , NeuroImage 2010, 50, 81. 20006716 10.1016/j.neuroimage.2009.12.011 PMC2827259

[57] X. Liu , J. H. Duyn , Proc. Natl. Acad. Sci. U.S.A. 2013, 110, 4392. 23440216 10.1073/pnas.1216856110 PMC3600481

[58] J. M. Huntenburg , P.‐L. Bazin , D. S. Margulies , Trends Cognit. Sci. 2018, 22, 21. 29203085 10.1016/j.tics.2017.11.002

[59] G. Shafiei , R. D. Markello , R. Vos de Wael , B. C. Bernhardt , B. D. Fulcher , B. Misic , elife 2020, 9, 62116. 10.7554/eLife.62116 PMC7771969 33331819

[60] S. Krohn , N. Von Schwanenflug , L. Waschke , A. Romanello , M. Gell , D. D. Garrett , C. Finke , Sci. Adv. 2023, 9, abq3851. 10.1126/sciadv.abq3851 PMC9891702 36724223

[61] R. V. Raut , A. Z. Snyder , A. Mitra , D. Yellin , N. Fujii , R. Malach , M. E. Raichle , Sci. Adv. 2021, 7, abf2709. 10.1126/sciadv.abf2709 PMC8294763 34290088

[62] R. Chaudhuri , K. Knoblauch , M.‐A. Gariel , H. Kennedy , X.‐J. Wang , Neuron 2015, 88, 419. 26439530 10.1016/j.neuron.2015.09.008 PMC4630024

[63] J. H. Lee , R. Durand , V. Gradinaru , F. Zhang , I. Goshen , D.‐S. Kim , L. E. Fenno , C. Ramakrishnan , K. Deisseroth , Nature 2010, 465, 788. 20473285 10.1038/nature09108 PMC3177305

[64] V. Menon , L. Q. Uddin , Brain Struct. Funct. 2010, 214, 655. 20512370 10.1007/s00429-010-0262-0 PMC2899886

[65] T. White , V. D. Calhoun , J. Exp. Neurosci. 2019, 13, 1179069519851809. 31210734 10.1177/1179069519851809 PMC6545633

[66] E. A. Crone , R. E. Dahl , Nat. Rev. Neurosci. 2012, 13, 636. 22903221 10.1038/nrn3313

[67] R. N. Spreng , E. Dimas , L. Mwilambwe‐Tshilobo , A. Dagher , P. Koellinger , G. Nave , A. Ong , J. M. Kernbach , T. V. Wiecki , T. Ge , Y. Li , A. J. Holmes , B. T. T. Yeo , G. R. Turner , R. I. M. Dunbar , D. Bzdok , Nat. Commun. 2020, 11, 6393. 33319780 10.1038/s41467-020-20039-w PMC7738683

[68] J. Engelmann , E. Damaraju , S. Padmala , L. Pessoa , Front. Hum. Neurosci. 2009, 3, 2009 10.3389/neuro.09.004.2009 PMC2679199 19434242

[69] D. M. Small , D. Gitelman , K. Simmons , S. M. Bloise , T. Parrish , M.‐M. Mesulam , Cereb. Cortex 2005, 15, 1855. 15746002 10.1093/cercor/bhi063

[70] M. Jaber , J. Neural. Transm. (Vienna) 2023, 130, 425. 36318343 10.1007/s00702-022-02555-9

[71] Z. Li , Y.‐X. Zhu , L.‐J. Gu , Y. Cheng , Zool. Res. 2021, 42, 800. 34755500 10.24272/j.issn.2095-8137.2021.251 PMC8645879

[72] M. Varghese , N. Keshav , S. Jacot‐Descombes , T. Warda , B. Wicinski , D. L. Dickstein , H. Harony‐Nicolas , S. De Rubeis , E. Drapeau , J. D. Buxbaum , P. R. Hof , Acta Neuropathol. 2017, 134, 537. 28584888 10.1007/s00401-017-1736-4 PMC5693718

[73] L. Caselli , L. Chelazzi , PLoS One 2011, 6, 21489. 10.1371/journal.pone.0021489 PMC3123358 21720549

[74] N. Higo , Front. Syst. Neurosci. 2021, 15. 10.3389/fnsys.2021.760311 PMC8606408 34819842

[75] L. Li , Z. Liu , Neurosci. Bull. 2023, 39, 1561. 37258795 10.1007/s12264-023-01067-0 PMC10533465

[76] J. Bachevalier , K. A. Loveland , Neurosci. Biobeh. Rev. 2006, 30, 97. 10.1016/j.neubiorev.2005.07.002 16157377

[77] G. S. Dichter , J. N. Felder , J. W. Bodfish , Soc. Cogn. Affect Neurosci. 2009, 4, 215. 19574440 10.1093/scan/nsp017 PMC2728636

[78] P. Duret , F. Samson , B. Pinsard , E. B. Barbeau , A. Boré , I. Soulières , L. Mottron , NeuroImage Clin. 2018, 20, 415. 30128280 10.1016/j.nicl.2018.04.036 PMC6095946

[79] J. O'Doherty , M. L. Kringelbach , E. T. Rolls , J. Hornak , C. Andrews , Nat. Neurosci. 2001, 4, 95. 11135651 10.1038/82959

[80] J. Salmi , U. Roine , E. Glerean , J. Lahnakoski , T. Nieminen‐von Wendt , P. Tani , S. Leppämäki , L. Nummenmaa , I. P. Jääskeläinen , S. Carlson , P. Rintahaka , M. Sams , NeuroImage Clin. 2013, 3, 489. 24273731 10.1016/j.nicl.2013.10.011 PMC3830058

[81] N. F. Wymbs , M. B. Nebel , J. B. Ewen , S. H. Mostofsky , Cereb. Cortex 2021, 31, 2639. 33386399 10.1093/cercor/bhaa380 PMC8023826

[82] A. Kepecs , G. Fishell , Nature 2014, 505, 318. 24429630 10.1038/nature12983 PMC4349583

[83] K. M. Anderson , M. A. Collins , R. Chin , T. Ge , M. D. Rosenberg , A. J. Holmes , Nat. Commun. 2020, 11, 2889. 32514083 10.1038/s41467-020-16710-x PMC7280213

[84] Y. Kim , G. R. Yang , K. Pradhan , K. U. Venkataraju , M. Bota , L. C. García Del Molino , G. Fitzgerald , K. Ram , M. He , J. M. Levine , P. Mitra , Z. J. Huang , X.‐J. Wang , P. Osten , Cell 2017, 171, 456. 28985566 10.1016/j.cell.2017.09.020 PMC5870827

[85] X.‐J. Wang , J. Tegnér , C. Constantinidis , P. Goldman‐Rakic , Proc. Natl. Acad. Sci. USA 2004, 101, 1368. 14742867 10.1073/pnas.0305337101 PMC337059

[86] E. L.‐L. Pai , D. Vogt , A. Clemente‐Perez , G. L. McKinsey , F. S. Cho , J. S. Hu , M. Wimer , A. Paul , S. Fazel Darbandi , R. Pla , T. J. Nowakowski , L. V. Goodrich , J. T. Paz , J. L. R. Rubenstein , Cell Rep. 2019, 26, 1157. 30699346 10.1016/j.celrep.2019.01.031 PMC6602795

[87] X. Liu , N. Novosedlik , A. Wang , M. L. Hudson , I. L. Cohen , A. E. Chudley , C. J. Forster‐Gibson , S. M. E. Lewis , J. J. A. Holden , Eur. J. Hum. Genet. 2009, 17, 228. 18728693 10.1038/ejhg.2008.148 PMC2986060

[88] G. Vakilzadeh , V. Martinez‐Cerdeño , NDT 2023, 19, 841. 10.2147/NDT.S390053 PMC10106330 37077706

[89] R. K. Sigaard , M. Kjær , B. Pakkenberg , Cereb. Cortex 2016, 26, 89. 25122465 10.1093/cercor/bhu178

[90] Y. Xiong , J. Chen , Y. Li , Front. Neurosci. 2023, 17, 1125428. 37021129 10.3389/fnins.2023.1125428 PMC10067592

[91] D. Attwell , A. M. Buchan , S. Charpak , M. Lauritzen , B. A. MacVicar , E. A. Newman , Nature 2010, 468, 232. 21068832 10.1038/nature09613 PMC3206737

[92] A. Kocharyan , P. Fernandes , X.‐K. Tong , E. Vaucher , E. Hamel , J. Cereb. Blood Flow Metab. 2008, 28, 221. 17895909 10.1038/sj.jcbfm.9600558

[93] K. Lepeta , M. V. Lourenco , B. C. Schweitzer , P. V. Martino Adami , P. Banerjee , S. Catuara‐Solarz , M. de La Fuente Revenga , A. M. Guillem , M. Haidar , O. M. Ijomone , B. Nadorp , L. Qi , N. D. Perera , L. K. Refsgaard , K. M. Reid , M. Sabbar , A. Sahoo , N. Schaefer , R. K. Sheean , A. Suska , R. Verma , C. Vicidomini , D. Wright , X.‐D. Zhang , C. Seidenbecher , J. Neurochem. 2016, 138, 785. 27333343 10.1111/jnc.13713 PMC5095804

[94] W. Li , L. Pozzo‐Miller , J. Neurosci. Res. 2020, 98, 2130. 31758607 10.1002/jnr.24560 PMC7242149

[95] R. Reig‐Viader , C. Sindreu , À. Bayés , Prog. Neuro‐Psychopharmacol. Biol. Psychiatry 2018, 84, 353. 10.1016/j.pnpbp.2017.09.011 28941771

[96] X. Wang , R. Kery , Q. Xiong , Prog. Neuro‐Psychopharmacol. Biol. Psychiatry 2018, 84, 398. 10.1016/j.pnpbp.2017.09.026 28986278

[97] Z. Yan , B. Rein , Mol. Psychiatry 2022, 27, 445. 33875802 10.1038/s41380-021-01092-3 PMC8523584

[98] S. Maier , A. L. Düppers , K. Runge , M. Dacko , T. Lange , T. Fangmeier , A. Riedel , D. Ebert , D. Endres , K. Domschke , E. Perlov , K. Nickel , L. Tebartz Van Elst , Autism Res. 2022, 15, 1222. 35587691 10.1002/aur.2740

[99] J. Li , P. Rubini , Y. Tang , P. Illes , Neurosci. Bull. 2022, 38, 104. 34748187 10.1007/s12264-021-00788-4 PMC8782982

[100] A. S. F. Berry , B. M. Finucane , S. M. Myers , L. K. Walsh , J. M. Seibert , C. L. Martin , D. H. Ledbetter , M. T. Oetjens , Nat. Commun. 2024, 15, 8897. 39406744 10.1038/s41467-024-53211-7 PMC11480344

[101] S. V. Mullegama , L. Pugliesi , B. Burns , Z. Shah , R. Tahir , Y. Gu , D. L. Nelson , S. H. Elsea , Eur. J. Hum. Genet. 2015, 23, 781. 25271084 10.1038/ejhg.2014.200 PMC4795052

[102] S. Berto , A. H. Treacher , E. Caglayan , D. Luo , J. R. Haney , M. J. Gandal , D. H. Geschwind , A. A. Montillo , G. Konopka , Nat. Commun. 2022, 13, 3328. 35680911 10.1038/s41467-022-31053-5 PMC9184501

[103] E. Golovina , T. Fadason , T. J. Lints , C. Walker , M. H. Vickers , J. M. O'Sullivan , Sci. Rep. 2021, 11, 15867. 34354167 10.1038/s41598-021-95447-z PMC8342620

[104] A. Di Martino , C.‐G. Yan , Q. Li , E. Denio , F. X. Castellanos , K. Alaerts , J. S. Anderson , M. Assaf , S. Y. Bookheimer , M. Dapretto , Mol. Psychiatry 2014, 19, 659. 23774715 10.1038/mp.2013.78 PMC4162310

[105] A. Di Martino , D. O'connor , B. Chen , K. Alaerts , J. S. Anderson , M. Assaf , J. H. Balsters , L. Baxter , A. Beggiato , S. Bernaerts , Sci. Data 2017, 4, 170010. 28291247 10.1038/sdata.2017.10 PMC5349246

[106] N. J. Tustison , B. B. Avants , P. A. Cook , Y. Zheng , A. Egan , P. A. Yushkevich , J. C. Gee , IEEE Trans. Med. Imaging 2010, 29, 1310. 20378467 10.1109/TMI.2010.2046908 PMC3071855

[107] Y. Zhang , M. Brady , S. Smith , IEEE Trans. Med. Imaging 2001, 20, 45. 11293691 10.1109/42.906424

[108] M. Reuter , H. D. Rosas , B. Fischl , Neuroimage 2010, 53, 1181. 20637289 10.1016/j.neuroimage.2010.07.020 PMC2946852

[109] A. M. Dale , B. Fischl , M. I. Sereno , NeuroImage 1999, 9, 179. 9931268 10.1006/nimg.1998.0395

[110] A. Klein , S. S. Ghosh , F. S. Bao , J. Giard , Y. Häme , E. Stavsky , N. Lee , B. Rossa , M. Reuter , E. C. Neto , PLoS Comput. Biol. 2017, 13, 1005350. 10.1371/journal.pcbi.1005350 PMC5322885 28231282

[111] A. C. Evans , A. L. Janke , D. L. Collins , S. Baillet , NeuroImage 2012, 62, 911. 22248580 10.1016/j.neuroimage.2012.01.024

[112] V. S. Fonov , A. C. Evans , R. C. McKinstry , C. R. Almli , D. L. Collins , NeuroImage 2009, 47, S102.

[113] M. Jenkinson , P. Bannister , M. Brady , S. Smith , NeuroImage 2002, 17, 825. 12377157 10.1016/s1053-8119(02)91132-8

[114] R. W. Cox , J. S. Hyde , NMR Biomed. 1997, 10, 171. 9430344 10.1002/(sici)1099-1492(199706/08)10:4/5<171::aid-nbm453>3.0.co;2-l

[115] D. N. Greve , B. Fischl , NeuroImage 2009, 48, 63. 19573611 10.1016/j.neuroimage.2009.06.060 PMC2733527

[116] Y. Behzadi , K. Restom , J. Liau , T. T. Liu , NeuroImage 2007, 37, 90. 17560126 10.1016/j.neuroimage.2007.04.042 PMC2214855

[117] R. H. Pruim , M. Mennes , D. van Rooij , A. Llera , J. K. Buitelaar , C. F. Beckmann , NeuroImage 2015, 112, 267. 25770991 10.1016/j.neuroimage.2015.02.064

[118] K. Murphy , R. M. Birn , D. A. Handwerker , T. B. Jones , P. A. Bandettini , NeuroImage 2009, 44, 893. 18976716 10.1016/j.neuroimage.2008.09.036 PMC2750906

[119] A. Schaefer , R. Kong , E. M. Gordon , T. O. Laumann , X.‐N. Zuo , A. J. Holmes , S. B. Eickhoff , B. T. T. Yeo , Cereb. Cortex 2018, 28, 3095. 28981612 10.1093/cercor/bhx179 PMC6095216

[120] B. Bostami , V. D. Calhoun , H. J. Van Der Horn , V. Vergara , in 2021 IEEE 21st International Conference on Bioinformatics and Bioengineering (BIBE), IEEE, Piscataway, 2021, pp. 1–4.

[121] D. S. Margulies , S. S. Ghosh , A. Goulas , M. Falkiewicz , J. M. Huntenburg , G. Langs , G. Bezgin , S. B. Eickhoff , F. X. Castellanos , M. Petrides , E. Jefferies , J. Smallwood , Proc. Natl. Acad. Sci. U.S.A. 2016, 113, 12574. 27791099 10.1073/pnas.1608282113 PMC5098630

[122] R. D. Markello , J. Y. Hansen , Z.‐Q. Liu , V. Bazinet , G. Shafiei , L. E. Suárez , N. Blostein , J. Seidlitz , S. Baillet , T. D. Satterthwaite , Nat. Methods 2022, 19, 1472. 36203018 10.1038/s41592-022-01625-w PMC9636018

[123] A. F. Alexander‐Bloch , H. Shou , S. Liu , T. D. Satterthwaite , D. C. Glahn , R. T. Shinohara , S. N. Vandekar , A. Raznahan , NeuroImage 2018, 178, 540. 29860082 10.1016/j.neuroimage.2018.05.070 PMC6095687

[124] M. J. Hawrylycz , E. S. Lein , A. L. Guillozet‐Bongaarts , E. H. Shen , L. Ng , J. A. Miller , L. N. Van De Lagemaat , K. A. Smith , A. Ebbert , Z. L. Riley , Nature 2012, 489, 391. 22996553 10.1038/nature11405 PMC4243026

[125] A. Arnatkevic̆iūtė , B. D. Fulcher , A. Fornito , NeuroImage 2019, 189, 353. 30648605 10.1016/j.neuroimage.2019.01.011

[126] R. D. Markello , A. Arnatkeviciute , J.‐B. Poline , B. D. Fulcher , A. Fornito , B. Misic , elife 2021, 10, 72129. 10.7554/eLife.72129 PMC8660024 34783653

[127] S. E. Morgan , J. Seidlitz , K. J. Whitaker , R. Romero‐Garcia , N. E. Clifton , C. Scarpazza , T. Van Amelsvoort , M. Marcelis , J. Van Os , G. Donohoe , D. Mothersill , A. Corvin , A. Pocklington , A. Raznahan , P. McGuire , P. E. Vértes , E. T. Bullmore , Proc. Natl. Acad. Sci. U.S.A. 2019, 116, 9604. 31004051 10.1073/pnas.1820754116 PMC6511038

[128] P. E. Vértes , T. Rittman , K. J. Whitaker , R. Romero‐Garcia , F. Váša , M. G. Kitzbichler , K. Wagstyl , P. Fonagy , R. J. Dolan , P. B. Jones , I. M. Goodyer , Philos. Trans. R. Soc. London, Ser. B 2016, 371, 20150362. 27574314 10.1098/rstb.2015.0362 PMC5003862

[129] Y. Zhou , B. Zhou , L. Pache , M. Chang , A. H. Khodabakhshi , O. Tanaseichuk , C. Benner , S. K. Chanda , Nat. Commun. 2019, 10, 1523. 30944313 10.1038/s41467-019-09234-6 PMC6447622

[130] D. Velmeshev , L. Schirmer , D. Jung , M. Haeussler , Y. Perez , S. Mayer , A. Bhaduri , N. Goyal , D. H. Rowitch , A. R. Kriegstein , Science 2019, 364, 685. 31097668 10.1126/science.aav8130 PMC7678724

[131] G. X. Y. Zheng , J. M. Terry , P. Belgrader , P. Ryvkin , Z. W. Bent , R. Wilson , S. B. Ziraldo , T. D. Wheeler , G. P. McDermott , J. Zhu , M. T. Gregory , J. Shuga , L. Montesclaros , J. G. Underwood , D. A. Masquelier , S. Y. Nishimura , M. Schnall‐Levin , P. W. Wyatt , C. M. Hindson , R. Bharadwaj , A. Wong , K. D. Ness , L. W. Beppu , H. J. Deeg , C. McFarland , K. R. Loeb , W. J. Valente , N. G. Ericson , E. A. Stevens , J. P. Radich , et al., Nat. Commun. 2017, 8, 14049. 28091601 10.1038/ncomms14049 PMC5241818

[132] C. S. McGinnis , L. M. Murrow , Z. J. Gartner , Cells 2019, 8, 329.30965679

[133] S. Yang , S. E. Corbett , Y. Koga , Z. Wang , W. E. Johnson , M. Yajima , J. D. Campbell , Genome Biol. 2020, 21, 57. 32138770 10.1186/s13059-020-1950-6 PMC7059395

[134] T. Stuart , A. Butler , P. Hoffman , C. Hafemeister , E. Papalexi , W. M. Mauck , Y. Hao , M. Stoeckius , P. Smibert , R. Satija , Cell 2019, 177, 1888. 31178118 10.1016/j.cell.2019.05.031 PMC6687398

[135] G. Yu , L.‐G. Wang , Y. Han , Q.‐Y. He , OMICS 2012, 16, 284. 22455463 10.1089/omi.2011.0118 PMC3339379

[136] B. Van de Sande , C. Flerin , K. Davie , M. De Waegeneer , G. Hulselmans , S. Aibar , R. Seurinck , W. Saelens , R. Cannoodt , Q. Rouchon , T. Verbeiren , D. De Maeyer , J. Reumers , Y. Saeys , S. Aerts , Nat. Protoc. 2020, 15, 2247. 32561888 10.1038/s41596-020-0336-2

[137] X. Qiu , Q. Mao , Y. Tang , L. Wang , R. Chawla , H. A. Pliner , C. Trapnell , Nat. Methods 2017, 14, 979. 28825705 10.1038/nmeth.4402 PMC5764547

