# Supplementary material for: Disentangling the Switching Behavior in Functional Connectivity Dynamics in Autism Spectrum Disorder: Insights from Developmental Cohort Analysis and Molecular‐Cellular Associations
Source: Adv Sci (Weinh). 2025 May 9;12(20):2403801. doi: 10.1002/advs.202403801 (PMC12120798; doi:10.1002/advs.202403801)
Supplement: Supplementary file 1 — Supporting Information [file ADVS-12-2403801-s001.docx]

Supporting Information

**Title:** **Cellular and Molecular Basis Underlying Aberrant Cortical Dynamics Driving Patterns in Developmental Autism Spectrum Disorder**

*Wei Li, Xia Qiu, Jin Chen, Kexuan Chen, Meiling Chen,* *Yinyan Wang, Wenjie Sun, Jing Su, Yongchang Chen, Xiaobao Liu^*^, Congying Chu^*^, Jiaojian Wang^*^*

W. Li, X. Qiu, J. Chen, W. Sun, J. Su, Y. Chen, J. Wang

State Key Laboratory of Primate Biomedical Research, Institute of Primate Translational Medicine, Kunming University of Science and Technology, Kunming 650500, China

E-mail: [jiaojianwang@uestc.edu.cn](mailto:jiaojianwang@uestc.edu.cn)

W. Li, X. Liu

Faculty of Mechanical and Electrical Engineering, Kunming University of Science and Technology, Kunming 650500, China

E-mail: [forcan2008@kust.edu.cn](mailto:forcan2008@kust.edu.cn)

K. Chen

Medical School, Kunming University of Science and Technology, Kunming 650500, China

M. Chen

Department of Clinical Psychology, the First People’s Hospital of Yunnan Province, The Affiliated Hospital of Kunming University of Science and Technology, Kunming 650500, China

Y. Wang

Department of Neurosurgery, Beijing Tiantan Hospital, Capital Medical University, Beijing 100070, China

C. Chu

Brainnetome Center & National Laboratory of Pattern Recognition, Institute of Automation, Chinese Academy of Sciences, Beijing 100190, China

E-mail: [chucongying@gmail.com](mailto:chucongying@gmail.com)

J. Wang

Yunnan Key Laboratory of Primate Biomedical Research, Kunming 650500, China

**Supplementary Table**

**Table S1.** Scanning parameters for each site.

| **ABIDE I Collections:** | | | | | | |
| --- | --- | --- | --- | --- | --- | --- |
| **SITE ID** | **MRI Scanner** | **TR[ms]** | **TE[ms]** | **Voxel size** | **Field Strength** | **Sequence name** |
| **Carnegie Mellon University** | SIEMENS MAGNETOM Verio syngo MR B17 | a2000/b1500 | 30 | 3.0×3.0×3.0 | 3T | BOLD |
| **Kennedy Krieger Institute** | Philips Achieva | 2500 | 30 | 3.0×3.0×3.0 | 3T | BOLD |
| **University of Leuven: Sample 1** | Philips Intera | 1667 | 33 | 3.59×3.59×4 | 3T | BOLD |
| **NYU Langone Medical Center** | SIEMENS MAGNETOM Allegra syngo MR 2004A | 2000 | 15 | 3.0×3.0×4.0 | 3T | BOLD |
| **Oregon Health and Science University** | SIEMENS MAGNETOM TrioTim syngo MR B17 | 2500 | 30 | 3.8×3.8×3.8 | 3T | BOLD |
| **Olin, Institute of Living at Hartford Hospital** | SIEMENS MAGNETOM Allegra syngo MR 2004A | 1500 | 27 | 3.4×3.4×4.0 | 3T | BOLD |
| **Social Brain Lab BCN NIC UMC Groningen and Netherlands Institute for Neurosciences** | Philips Intera | 2200 | 30 | 2.75×2.75×2.72 | 3T | BOLD |
| **Trinity Centre for Health Sciences** | Philips Achieva | 2000 | 28 | 3×3×3.5 | 3T | BOLD |
| **University of California, Los Angeles: Sample 1** | SIEMENS MAGNETOM TrioTim syngo MR B15 | 3000 | 28 | 3.0×3.0×4.0 | 3T | BOLD |
| **University of California, Los Angeles: Sample 2** | SIEMENS MAGNETOM TrioTim syngo MR B15 | 3000 | 28 | 3.0×3.0×4.0 | 3T | BOLD |
| **University of Michigan: Sample 1** | Signa | 2000 | 30 | 3.438×3.438×3.0 | 3T | BOLD |
| **University of Michigan: Sample 2** | Signa | 2000 | 30 | 3.438×3.438×3.0 | 3T | BOLD |
| **University of Utah School of Medicine** | SIEMENS MAGNETOM TrioTim syngo MR B17 | 2000 | 28 | 3.4×3.4×3.0 | 3T | BOLD |
| **Yale Child Study Center** | SIEMENS MAGNETOM TrioTim syngo MR B17 | 2000 | 25 | 3.4×3.4×4.0 | 3T | BOLD |
| **California Institute of Technology** | SIEMENS MAGNETOM TrioTim syngo MR B17 | 2000 | 30 | 3.5×3.5×3.5 | 3T | BOLD |
| **University of Pittsburgh School of Medicine** | SIEMENS MAGNETOM Allegra syngo MR A30 | 1500 | 25 | 3.1×3.1×4.0 | 3T | BOLD |
| **Ludwig Maximilians University Munich** | SIEMENS MAGNETOM Verio syngo MR B17 | 3000 | 30 | 3.0×3.0×4.0、3.0×3.0×3.0 | 3T | BOLD |
| **ABIDE II Collections:** | | | | | | |
| **SITE ID** | **MRI Scanner** | **TR[ms]** | **TE[ms]** | **Voxel size** | **Field Strength** | **Sequence name** |
| **Barrow Neurological Institute** | Philips Ingenia | 3000 | 25 | 3.75×3.75×4.00 | 3T | BOLD |
| **Erasmus University Medical Center Rotterdam** | General Electric Discovery MR750 | 2000 | 30 | 3.5938×3.5938×4 | 3T | BOLD |
| **ETH Zürich** | Phillips Achieva | 2000 | 25 | 3×3.1×3 | 3T | BOLD |
| **Georgetown University** | SIEMENS MAGNETOM TrioTim syngo MR B17 | 2000 | 30 | 3×3×2.5 | 3T | BOLD |
| **Indiana University** | SIEMENS MAGNETOM TrioTim syngo MR B17 | 813 | 28 | 3.4×3.4×3.4 | 3T | BOLD |
| **Kennedy Krieger Institute** | Philips Achieva | 2500 | 30 | 3.05×3.15×3.00 | 3T | BOLD |
| **Katholieke Universiteit Leuven** | Philips | 2500 | 30 | 2.5×2.56×2.7 | 3T | BOLD |
| **Oregon Health and Science University** | SIEMENS MAGNETOM TrioTim syngo MR B17 | 2500 | 30 | 3.8×3.8×3.8 | 3T | BOLD |
| **Olin Neuropsychiatry Research Center, Institute of Living at Hartford Hospital** | SIEMENS MAGNETOM Skyra syngo MR D13 | 450 | 30 | 3.0×3.0×3.0 | 3T | BOLD |
| **San Diego State University** | General Electric Discovery MR750 | 2000 | 30 | 3.4375×3.4375×3.4 | 3T | BOLD |
| **University of California Davis** | SIEMENS MAGNETOM TrioTim syngo MR B17 | 2000 | 24 | 3.5×3.5×4 | 3T | BOLD |
| **University of California Los Angeles** | SIEMENS MAGNETOM TrioTim syngo MR B17 | 3000 | 28 | 3.0×3.0×4.0 | 3T | BOLD |
| **University of California Los Angeles: Longitudinal Sample** | SIEMENS MAGNETOM TrioTim syngo MR B17 | 3000 | 28 | 3.0×3.0×4.0 | 3T | BOLD |
| **University of Pittsburgh School of Medicine: Longitudinal Sample** | SIEMENS MAGNETOM Allegra syngo MR A30 | 1500 | 25 | 3.1×3.1×4.0 | 3T | BOLD |
| **Trinity Centre for Health Sciences** | Philips Intera Achieva | 2000 | 27 | 3.0×3.0×3.2 | 3T | BOLD |
| **University of Utah School of Medicine** | SIEMENS MAGNETOM TrioTim syngo MR B17 | 2000 | 28 | 3.4×3.4×3.0 | 3T | BOLD |

**Table S2.** Demographic information for matched subjects from ABIDE I and II database.

|  | **Children** | | | | **Adolescents** | | | **Adults** | | |
| --- | --- | --- | --- | --- | --- | --- | --- | --- | --- | --- |
|  | ASD | | TC | p-value | ASD | TC | p-value | ASD | TC | p-value |
| **Participants matched after preprocessing (n)** | 253 | | 345 | \ | 293 | 252 | \ | 274 | 283 | \ |
| **Number of participants from each site** | | 2^1^,4^2^,7^3^,  19^5^,12^7^,16^8^,  37^9^,13^10^,5^11^,  8^12^,29^13^,15^14^,  19^15^,4^16^,26^17^,  2^18^,9^19^,26^20^ | 2^1^,15^3^,16^5^,  4^6^,4^7^,28^8^,  31^9^,9^10^,5^11^,  4^12^,27^13^,10^14^,  49^15^,3^16^,96^17^,2^18^,13^19^,27^20^ | \ | 11^1^,13^2^,6^3^,  13^4^,25^5^,20^6^,  16^7^,21^9^,20^10^,  5^11^,2^12^,9^13^,  18^14^,21^15^,11^16^,7^17^,9^18^,7^19^,  3^25^,2^26^,2^28^,  4^29^,6^30^,10^31^,  18^32^,14^33^ | 12^1^,8^2^,13^4^,  28^5^,8^6^,10^7^,  3^8^,36^9^,19^10^,  6^11^,2^12^,9^13^,  14^14^,14^15^,12^16^,  13^17^,3^19^,2^26^,  7^30^,12^31^,14^32^,  7^33^ | \ | 10^1^,9^2^,10^4^,  2^5^,37^6^,17^9^,  16^12^,6^16^,6^18^,  10^21^,14^22^,17^23^,  13^24^,18^25^,29^26^,  11^27^,19^28^,28^29^,  2^30^ | 12^1^,6^2^,9^4^,  8^5^,28^6^,27^9^,  27^12^,7^16^,12^18^,  11^21^,15^22^,18^23^,  14^24^,29^25^,29^26^,11^27^,20^28^ | \ |
| **Age in years (mean ± std, range)** | | 9.88$\pm$1.46 | 9.85$\pm$1.28 | 0.22^34^ | 14.68$\pm$1.82 | 14.53$\pm$1.76 | 0.56^34^ | 26.56$\pm$9.90 | 26.95$\pm$8.86 | 0.05^34^ |
| **Sex** | | 38 females | 106 females | \ | 32 females | 48 females | \ | 26 females | 40 females | \ |

**ABIDE I Collections:**

^1^ University of Pittsburgh School of Medicine

^2^ Olin, Institute of Living at Hartford Hospital

^3^ Oregon Health and Science University

^4^ Trinity Centre for Health Sciences

^5^ University of Michigan: Sample 1

^6^ University of Utah School of Medicine

^7^ Yale Child Study Center

^8^ Kennedy Krieger Institute

^9^ NYU Langone Medical Center

^10^ University of California, Los Angeles: Sample 1

^11^ University of California, Los Angeles: Sample 2

^12^ Ludwig Maximilians University Munich

^21^ Carnegie Mellon University

^22^ University of Leuven: Sample 1

^23^ California Institute of Technology

^24^ Social Brain Lab BCN NIC UMC Groningen and Netherlands Institute for Neurosciences

^31^ University of Michigan: Sample 2

**ABIDE II Collections:**

^13^ Georgetown University

^14^ San Diego State University

^15^ Oregon Health and Science University

^16^ Trinity Centre for Health Sciences

^17^ Kennedy Krieger Institute

^18^ University of Utah School of Medicine

^19^ University of California Los Angeles

^20^ Erasmus University Medical Center Rotterdam

^25^ Olin Neuropsychiatry Research Center, Institute of Living at Hartford Hospital

^26^ Barrow Neurological Institute

^27^ ETH Zürich

^28^ Indiana University

^29^ Katholieke Universiteit Leuven

^30^ University of Pittsburgh School of Medicine: Longitudinal Sample

^32^ University of California Davis

^33^ University of California Los Angeles: Longitudinal Sample

^34^ Using Kolmogorov-Smirnov test (${ks}_{Children}=0.09,{ks}_{Adolescents}=0.07,{ks}_{Adults}=0.11$)

**Table S3.** Demographic and clinical information on participants.

| **Scale** | **Children (ASD)** | **Adolescents (ASD)** | **Adults (ASD)** | ***p*-value^9^** |
| --- | --- | --- | --- | --- |
| **ADI_RRB_TOTAL_C^1^ (Number of subjects, Behavioral score)** | $n$=204, score=6.08$\pm$2.52 | $n$=185, score=5.94$\pm$2.46 | $n$=88, score=5.61$\pm$2.57 | 0.017 |
| **ADI_R_ONSET_TOTAL_D^2^ (Number of subjects, Behavioral score)** | $n$=180, score=3.15$\pm$1.23 | $n$=150, score=3.05$\pm$1.33 | $n$=85, score=2.84$\pm$1.47 | 0.018 |
| **ADI_R_SOCIAL_TOTAL_A^3^ (Number of subjects, Behavioral score)** | $n$=204, score=19.63$\pm$5.52 | $n$=184, score=19.65$\pm$5.12 | $n$=88, score=20.24$\pm$6.18 | 0.005 |
| **ADI_R_VERBAL_TOTAL_BV^4^ (Number of subjects, Behavioral score)** | $n$=204, score=15.73$\pm$4.59 | $n$=185, score=15.31$\pm$4.23 | $n$=88, score=15.90$\pm$5.24 | 0.164 |
| **ADOS_COMM^5^ (Number of subjects, Behavioral score)** | $n$=127, score=3.51$\pm$1.49 | $n$=142, score=3.75$\pm$1.72 | $n$=216, score=3.64$\pm$1.52 | 0.007 |
| **ADOS_SOCIAL^6^ (Number of subjects, Behavioral score)** | $n$=127, score =7.98$\pm$2.73 | $n$=143, score=7.86$\pm$2.92 | $n$=216, score=7.34$\pm$2.74 | 0.045 |
| **ADOS_STEREO_BEHAV^7^ (Number of subjects, Behavioral score)** | $n$=117, score=2.49$\pm$1.64 | $n$=126, score=1.74$\pm$1.59 | $n$=199, score=1.55$\pm$1.39 | 0.001 |
| **ADOS_TOTAL^8^ (Number of subjects, Behavioral score)** | $n$=127, score=11.64$\pm$3.79 | $n$=156, score=11.49$\pm$4.15 | $n$=226, score=10.98$\pm$3.76 | 0.007 |

**Note:** All subjects selected from the ABIDE I and ABIDE II datasets containing clinical scores, scores are shown in $mean\pm SD$.

The selected scales include:

^1^Restricted, Repetitive, and Stereotyped Patterns of Behavior Subscore (C)Total for Autism Diagnostic Interview-Revised

^2^Abnormality of Development Evident at or Before 36 Months Subscore (D)Total for Autism Diagnostic Interview-Revised

^3^Reciprocal Social Interaction Subscore (A)Total for Autism DiagnosticInterview-Revised

^4^Abnormalities in Communication Subscore (B) Total for Autism DiagnosticInterview-Revised

^5^Communication Total Subscore of the Classic ADOS

^6^Social Total Subscore of the Classic ADOS

^7^Sterotyped Behaviors and Restricted Interests Total Subscore of the Classic ADOS

^8^Classic Total ADOS Score (Communication subscore + Social Interactionsubscore)

^9^Using the Jarque-Bera test for whole age

**Table S4.** Volumetric PET images of different neurotransmitter receptors and transporters collected in multiple studies.

| **Annotation** | **Description** | **Tags** | **N (males)** | **Age (years)** | **Study** |
| --- | --- | --- | --- | --- | --- |
| ('aghourian2017', 'feobv') | PET tracer binding (SUVR) to VAChT (acetylcholine transporter) | receptors; PET | 18 (5) | 66.8 +/- 6.8 | [Aghourian et al., 2017](#Aghourian) |
| ('alarkurtti2015', 'raclopride') | PET tracer binding (BPnd) to D2 (dopamine receptor) | receptors; PET | 7 (7) | 24 +/- 2 | [Alakurtti et al., 2015](#Alakurtti) |
| ('bedard2019', 'feobv') | PET tracer binding (SUVR) to VAChT (acetylcholine transporter) | receptors; PET | 5 (4) | 68.3 +/- 3.1 | [Bedard et al., 2019](#Bedard) |
| ('beliveau2017', 'az10419369') | PET tracer binding (Bmax) to 5-HT1b (serotonin receptor) | receptors; PET | 36 (24) | 27.8 +/- 6.9 | [Beliveau et al., 2017](#Beliveau) |
| ('beliveau2017', 'cimbi36') | PET tracer binding (Bmax) to 5-HT2a (serotonin receptor) | receptors; PET | 29 (15) | 22.6 +/- 2.7 | [Beliveau et al., 2017](#Beliveau) |
| ('beliveau2017', 'cumi101') | PET tracer binding (Bmax) to 5-HT1a (serotonin receptor) | receptors; PET | 8 (3) | 28.4 +/- 8.8 | [Beliveau et al., 2017](#Beliveau) |
| ('beliveau2017', 'dasb') | PET tracer binding (Bmax) to 5-HTT (serotonin transporter) | receptors; PET | 100 (29) | 25.1 +/- 5.8 | [Beliveau et al., 2017](#Beliveau) |
| ('beliveau2017', 'sb207145') | PET tracer binding (Bmax) to 5-HT4 (serotonin receptor) | receptors; PET | 59 (41) | 25.9 +/- 5.3 | [Beliveau et al., 2017](#Beliveau) |
| ('ding2010', 'mrb') | PET tracer binding (BPnd) to NET (norepinephrine transporter) | receptors; PET | 77 (50) | 33.4 +/- 9.17 | [Ding et al., 2010](#Ding) |
| ('dubois2015', 'abp688') | PET tracer binding (BPnd) to mGluR5 (glutamate receptor) | receptors; PET | 28 (15) | 33.1 +/- 11.2 | [DuBois et al., 2016](#DuBois) |
| ('dukart2018', 'flumazenil') | PET tracer binding (BPnd) to GABAa (gaba receptor) | receptors; PET | 6 (6) | 43 +/- 4 | [Dukart et al., 2018](#Dukart) |
| ('dukart2018', 'fpcit') | SPECT tracer binding (SUVR) to DAT (dopamine transporter) | receptors; PET | 174 (109) | 61 +/- 11 | [Dukart et al., 2018](#Dukart) |
| ('fazio2016', 'madam') | PET tracer binding (BPnd) to 5-HTT (serotonin transporter) | receptors; PET | 10 (8) | 51-67 | [Fazio et al., 2016](#Fazio) |
| ('finnema2016', 'ucbj') | PET tracer binding (BPnd) to SV2A (synaptic vesicle glycoprotein 2A, a synapse marker) | PET | 76 (45) | 48.9 +/- 18.4 | [Finnema et al., 2018](#Finnema) |
| ('gallezot2010', 'p943') | PET tracer binding (BPnd) to 5-HT1b (serotonin receptor) | receptors; PET | 23 (15) | 28.7 +/- 7 | [Gallezot et al., 2010](#Gallezot) |
| ('gallezot2017', 'gsk189254') | PET tracer binding (Vt) to H3 (histamine receptor) | receptors; PET | 8 (7) | 31.69 +- 8.95 | [Gallezot et al., 2017](#Gallezot2017) |
| ('hesse2017', 'methylreboxetine') | PET tracer binding (BPnd) to NET (norepinephrine transporter) | receptors; PET | 10 (n/a) | 33.3 (mean) | [Hesse et al., 2017](#Hesse2017) |
| ('hillmer2016', 'flubatine') | PET tracer binding (Vt) to a4b2 (acetylcholine receptor) | receptors; PET | 30 (20) | 33.50 +/- 10.71 | [Hillmer et al., 2016](#Hillmer) |
| ('jaworska2020', 'fallypride') | PET tracer binding (BPnd) to D2 (dopamine receptor) | receptors; PET | 49 (16) | 18.41 $\pm$ 0.57 | [Jaworska et al., 2020](#Jaworska) |
| ('kaller2017', 'sch23390') | PET tracer binding (BPnd) to D1 (dopamine receptor) | receptors; PET | 13 (6) | 33 $\pm$ 13 | [Kaller et al., 2017](#Kaller) |
| ('kantonen2020', 'carfentanil') | PET tracer binding (BPnd) to MOR (mu-opioid receptor) | receptors; PET | 204 (132) | 32.3 $\pm$ 10.8 | [Kantonen et al., 2020](#Kantonen) |
| ('laurikainen2018', 'fmpepd2') | PET tracer binding (Vt) to CB1 (cannabinoid receptor) | receptors; PET | 22 (11) | 27.5 $\pm$ 8.05 | [Laurikainen et al., 2019](#Laurikainen) |
| ('naganawa2020', 'lsn3172176') | PET tracer binding (BPnd) to M1 (acetylcholine receptor) | receptors; PET | 24 (13) | 40.45 $\pm$ 11.71 | [Naganawa et al., 2021](#Naganawa) |
| ('norgaard2021', 'flumazenil') | PET and autoradiography informed GABAa benzodiazepine binding-site density (Bmax; gaba receptor) | receptors; PET | 16 (7) | 26.6 $\pm$ 8 | [Nørgaard et al., 2021](#Nørgaard) |
| ('normandin2015', 'omar') | PET tracer binding (Vt) to CB1 (cannabinoid receptor) | receptors; PET | 77 (49) | 30.01 $\pm$ 8.87 | [Normandin et al., 2015](#Normandin) |
| ('radnakrishnan2018', 'gsk215083') | PET tracer binding (BPnd) to 5-HT6 (serotonin receptor) | receptors; PET | 30 (30) | 36.6 $\pm$ 9.04 | [Radhakrishnan et al., 2018](#Radhakrishnan) |
| ('rosaneto', 'abp688') | PET tracer binding (BPnd) to mGluR5 (glutamate receptor) | receptors; PET | 22 (12) | 67.9 $\pm$ 9.6 | [Hansen et al., 2022](#Hansen) |
| ('sandiego2015', 'flb457') | PET tracer binding (BPnd) to D2 (dopamine receptor) | receptors; PET | 55 (26) | 32.45 $\pm$ 9.69 | [Sandiego et al., 2015](#Sandiego) |
| ('sasaki2012', 'fepe2i') | PET tracer binding (BPnd) to DAT (dopamine transporter) | receptors; PET | 6 (6) | 31.06 $\pm$ 7.7 | [Sasaki et al., 2012](#Sasaki) |
| ('savli2012', 'altanserin') | PET tracer binding (BPnd) to 5-HT2a (serotonin receptor) | receptors; PET | 19 (11) | 28.2 $\pm$ 5.7 | [Savli et al., 2012](#Savli) |
| ('savli2012', 'dasb') | PET tracer binding (BPnd) to 5-HTT (serotonin transporter) | receptors; PET | 18 (12) | 30.5 $\pm$ 9.5 | [Savli et al., 2012](#Savli) |
| ('savli2012', 'p943') | PET tracer binding (BPnd) to 5-HT1b (serotonin receptor) | receptors; PET | 23 (15) | 28.7 $\pm$ 7 | [Savli et al., 2012](#Savli) |
| ('savli2012', 'way100635') | PET tracer binding (BPnd) to 5-HT1a (serotonin receptor) | receptors; PET | 35 (18) | 26.3 $\pm$ 5.2 | [Savli et al., 2012](#Savli) |
| ('smart2019', 'abp688') | PET tracer binding (BPnd) to mGluR5 (glutamate receptor) | receptors; PET | 73 (25) | 19.9 $\pm$ 3.04 | [Smart et al., 2019](#Smart) |
| ('smith2017', 'flb457') | PET tracer binding (BPnd) to D2 (dopamine receptor) | receptors; PET | 37 (17) | 48.36 $\pm$ 16.93 | [Smith et al., 2017](#Smith) |
| ('tuominen', 'feobv') | PET tracer binding (SUVR) to VAChT (acetylcholine transporter) | receptors; PET | 4 (3) | 37 $\pm$ 10.2 | [Hansen et al., 2022](#Hansen) |
| ('turtonen2020', 'carfentanil') | PET tracer binding (BPnd) to MOR (mu-opioid receptor) | receptors; PET | 39 (19) | 39.38 +/- 5.05 | [Turtonen et al., 2021](#Turtonen) |

1. Age are shown in $mean\pm SD$.

**Table S5.** Demographic and single-cell samples information on participants.

| Sample | Age | Sex | Group | Age group |
| --- | --- | --- | --- | --- |
| PFC2949 | 6 | Male | TC | Children |
| PFC2927 | 7 | Male | ASD | Children |
| PFC2922 | 14 | Female | TC | Adolescents |
| PFC2920 | 15 | Male | TC | Adolescents |
| PFC2925 | 15 | Female | ASD | Adolescents |
| PFC2948 | 16 | Male | ASD | Adolescents |
| PFC2937 | 19 | Female | TC | Adults |
| PFC2923 | 19 | Male | TC | Adults |
| PFC2950 | 19 | Female | ASD | Adults |
| PFC2940 | 20 | Male | ASD | Adults |

**Table S6.** ANOVA effect size report.

|  | Age | | | Group | | | Group*Age | | |
| --- | --- | --- | --- | --- | --- | --- | --- | --- | --- |
| ROI | *F* | *P* | Partial Eta-Squared (η²) | *F* | *P* | Partial Eta-Squared (η²) | *F* | *P* | Partial Eta-Squared (η²) |
| 1 | 0.718839213 | 0.487466187 | 0.001 | 2.568098067 | 0.10922519 | 0.002 | 1.266733542 | 0.28201719 | 0.001 |
| 2 | 2.239221093 | 0.106856723 | 0.003 | 0.112957778 | 0.736843176 | 0 | 0.242704089 | 0.784530897 | 0 |
| 3 | 2.109440864 | 0.121624305 | 0.002 | 0.00965409 | 0.921741251 | 0 | 0.002052174 | 0.997949932 | 0 |
| 4 | 1.128079584 | 0.323897221 | 0.001 | 0.5485618 | 0.459008583 | 0 | 2.032522726 | 0.131323974 | 0.002 |
| 5 | 2.501231762 | 0.082286692 | 0.003 | 0.32373469 | 0.569447256 | 0 | 0.263110924 | 0.768688036 | 0 |
| 6 | 0.422772933 | 0.655296512 | 0 | 0.121400159 | 0.727564133 | 0 | 0.05983663 | 0.941920393 | 0 |
| 7 | 4.329565962 | 0.013319343 | 0.005 | 3.99852159 | 0.045699638 | 0.002 | 0.169025354 | 0.844501733 | 0 |
| 8 | 2.681195069 | 0.068771882 | 0.003 | 0.325221491 | 0.568562116 | 0 | 0.143543973 | 0.866293242 | 0 |
| 9 | 4.431557034 | 0.012034181 | 0.005 | 0.364897448 | 0.545879705 | 0 | 0.406700336 | 0.665908687 | 0 |
| 10 | 4.630404343 | 0.009874561 | 0.005 | 5.25839E-05 | 0.994215066 | 0 | 0.736218068 | 0.479074917 | 0.001 |
| 11 | 5.640284518 | 0.003618891 | 0.007 | 0.273722976 | 0.600913658 | 0 | 0.269338571 | 0.763917289 | 0 |
| 12 | 2.62879002 | 0.072460114 | 0.003 | 0.629954378 | 0.427483209 | 0 | 1.010421037 | 0.364284971 | 0.001 |
| 13 | 0.260388261 | 0.770783118 | 0 | 0.198309567 | 0.656145688 | 0 | 1.724136201 | 0.178639804 | 0.002 |
| 14 | 0.332507975 | 0.717169743 | 0 | 0.609880461 | 0.43494194 | 0 | 0.622746111 | 0.536591978 | 0.001 |
| 15 | 2.834963404 | 0.058999308 | 0.003 | 6.010076672 | 0.014324782 | 0.004 | 0.003954361 | 0.996053456 | 0 |
| 16 | 13.3256569 | 1.81048E-06 | 0.015 | 0.462159049 | 0.496709551 | 0 | 3.739950291 | 0.023951661 | 0.004 |
| 17 | 0.030602266 | 0.969861779 | 0 | 0.323413309 | 0.569638938 | 0 | 0.245930827 | 0.78200423 | 0 |
| 18 | 3.423185496 | 0.032834135 | 0.004 | 3.149799609 | 0.076115835 | 0.002 | 0.662031461 | 0.515935837 | 0.001 |
| 19 | 1.490690989 | 0.225512262 | 0.002 | 3.290088531 | 0.069876125 | 0.002 | 1.28766764 | 0.276183486 | 0.002 |
| 20 | 0.01123674 | 0.98882623 | 0 | 2.854707893 | 0.091290794 | 0.002 | 3.743895493 | 0.023857767 | 0.004 |
| 21 | 5.203648581 | 0.005584675 | 0.006 | 0.39297934 | 0.53082195 | 0 | 0.168942391 | 0.844571784 | 0 |
| 22 | 3.678053303 | 0.025474165 | 0.004 | 1.716609368 | 0.190307705 | 0.001 | 3.757128782 | 0.023545504 | 0.004 |
| 23 | 2.023108506 | 0.132563144 | 0.002 | 1.274644314 | 0.259057068 | 0.001 | 0.475521481 | 0.621643782 | 0.001 |
| 24 | 3.589340282 | 0.02782678 | 0.004 | 1.374940648 | 0.241129687 | 0.001 | 0.258021482 | 0.772608993 | 0 |
| 25 | 10.01193335 | 4.75741E-05 | 0.012 | 1.684084051 | 0.194559339 | 0.001 | 0.154263766 | 0.85705797 | 0 |
| 26 | 1.351149013 | 0.259221474 | 0.002 | 0.003056968 | 0.955914067 | 0 | 6.571227938 | 0.001436036 | 0.008 |
| 27 | 5.451318249 | 0.00436625 | 0.006 | 1.035540577 | 0.3090062 | 0.001 | 2.057594828 | 0.128080073 | 0.002 |
| 28 | 3.03870155 | 0.048158206 | 0.004 | 0.082158643 | 0.774428173 | 0 | 0.585532553 | 0.556921904 | 0.001 |
| 29 | 11.52327053 | 1.06966E-05 | 0.013 | 3.968615216 | 0.046516154 | 0.002 | 0.46717459 | 0.62685138 | 0.001 |
| 30 | 10.78698397 | 2.21234E-05 | 0.013 | 2.852893891 | 0.091393651 | 0.002 | 0.474171437 | 0.622483124 | 0.001 |
| 31 | 3.725542809 | 0.024297706 | 0.004 | 3.538358833 | 0.060136153 | 0.002 | 0.563805836 | 0.569146009 | 0.001 |
| 32 | 0.611965304 | 0.542403909 | 0.001 | 3.991662779 | 0.045885555 | 0.002 | 2.523470953 | 0.080482194 | 0.003 |
| 33 | 2.146684933 | 0.117188775 | 0.003 | 1.970277013 | 0.160601746 | 0.001 | 1.528002142 | 0.217267595 | 0.002 |
| 34 | 3.895421151 | 0.020517218 | 0.005 | 0.028796376 | 0.865270308 | 0 | 1.182370843 | 0.306803947 | 0.001 |
| 35 | 1.618563697 | 0.19848948 | 0.002 | 0.610167108 | 0.434834045 | 0 | 0.366675654 | 0.693089383 | 0 |
| 36 | 4.057238017 | 0.017465084 | 0.005 | 0.119858801 | 0.729230675 | 0 | 0.372791538 | 0.688865303 | 0 |
| 37 | 0.135323464 | 0.873442782 | 0 | 1.432327641 | 0.231552403 | 0.001 | 0.293870665 | 0.745410879 | 0 |
| 38 | 1.056944315 | 0.347745149 | 0.001 | 0.294022039 | 0.587725887 | 0 | 0.343588393 | 0.709270201 | 0 |
| 39 | 1.2677873 | 0.281720612 | 0.001 | 0.266562166 | 0.605714665 | 0 | 1.851323612 | 0.157346752 | 0.002 |
| 40 | 7.732455379 | 0.000454018 | 0.009 | 7.792977522 | 0.005303838 | 0.005 | 0.344101229 | 0.708906703 | 0 |
| 41 | 0.515764946 | 0.597137439 | 0.001 | 1.052073037 | 0.305176571 | 0.001 | 1.509569555 | 0.221302235 | 0.002 |
| 42 | 8.194286097 | 0.000287323 | 0.01 | 5.422645182 | 0.019994356 | 0.003 | 1.008641934 | 0.364932876 | 0.001 |
| 43 | 6.151533628 | 0.002178099 | 0.007 | 1.14540022 | 0.284666583 | 0.001 | 0.083254233 | 0.920120958 | 0 |
| 44 | 7.669848344 | 0.000483079 | 0.009 | 0.540755288 | 0.462221674 | 0 | 0.628632664 | 0.533444898 | 0.001 |
| 45 | 7.376155995 | 0.000646315 | 0.009 | 0.256914086 | 0.612314159 | 0 | 1.393055858 | 0.248599643 | 0.002 |
| 46 | 4.686037403 | 0.009343053 | 0.006 | 0.124416918 | 0.724336379 | 0 | 4.423152861 | 0.012135215 | 0.005 |
| 47 | 7.340730247 | 0.000669417 | 0.009 | 1.67653845 | 0.195561498 | 0.001 | 0.717469227 | 0.488133901 | 0.001 |
| 48 | 10.46396035 | 3.0437E-05 | 0.012 | 0.519729296 | 0.471056654 | 0 | 0.683750541 | 0.504859702 | 0.001 |
| 49 | 1.208623305 | 0.298865451 | 0.001 | 0.001413744 | 0.970011207 | 0 | 2.019092614 | 0.133095301 | 0.002 |
| 50 | 1.517788354 | 0.219494069 | 0.002 | 4.312234943 | 0.037989663 | 0.003 | 0.502649152 | 0.605016189 | 0.001 |
| 51 | 2.224369462 | 0.10845133 | 0.003 | 3.173222999 | 0.075033717 | 0.002 | 1.488014213 | 0.226115654 | 0.002 |
| 52 | 6.785888686 | 0.001160563 | 0.008 | 4.563429333 | 0.032804578 | 0.003 | 0.305673685 | 0.736667574 | 0 |
| 53 | 0.350685171 | 0.704256534 | 0 | 4.487354171 | 0.034291735 | 0.003 | 1.451897807 | 0.234416734 | 0.002 |
| 54 | 1.174336152 | 0.309275499 | 0.001 | 0.714419225 | 0.398099991 | 0 | 0.517860666 | 0.595888078 | 0.001 |
| 55 | 0.789360363 | 0.454302152 | 0.001 | 0.010614697 | 0.917953221 | 0 | 0.708286108 | 0.492633333 | 0.001 |
| 56 | 0.166166604 | 0.846918927 | 0 | 0.341402435 | 0.559098627 | 0 | 0.121355688 | 0.885726564 | 0 |
| 57 | 6.285976392 | 0.001905967 | 0.007 | 0.06309235 | 0.801703937 | 0 | 1.340601762 | 0.261965634 | 0.002 |
| 58 | 4.398264223 | 0.01243943 | 0.005 | 2.400319715 | 0.121497246 | 0.001 | 0.019404016 | 0.980783248 | 0 |
| 59 | 1.898285226 | 0.150143888 | 0.002 | 3.695338635 | 0.05473224 | 0.002 | 0.279439542 | 0.756242297 | 0 |
| 60 | 3.065379766 | 0.046894909 | 0.004 | 0.014954828 | 0.90268391 | 0 | 0.61065705 | 0.543113463 | 0.001 |
| 61 | 2.876461736 | 0.056608935 | 0.003 | 0.394593636 | 0.529979297 | 0 | 0.223518949 | 0.79972332 | 0 |
| 62 | 0.041624308 | 0.959231069 | 0 | 0.022154205 | 0.881695326 | 0 | 0.257067532 | 0.77334615 | 0 |
| 63 | 3.281983583 | 0.037792622 | 0.004 | 2.228217732 | 0.135696292 | 0.001 | 2.129347195 | 0.119233074 | 0.003 |
| 64 | 2.210200034 | 0.109994893 | 0.003 | 0.14072997 | 0.707603361 | 0 | 1.009391405 | 0.364659797 | 0.001 |
| 65 | 0.246226843 | 0.781772845 | 0 | 0.013257253 | 0.908347685 | 0 | 0.231194024 | 0.793610512 | 0 |
| 66 | 0.653567703 | 0.520317708 | 0.001 | 0.742449568 | 0.388999272 | 0 | 0.569645567 | 0.565834245 | 0.001 |
| 67 | 0.138522558 | 0.870653472 | 0 | 3.019223969 | 0.082464568 | 0.002 | 1.791089595 | 0.167094039 | 0.002 |
| 68 | 0.082332024 | 0.92096981 | 0 | 0.077165253 | 0.781210272 | 0 | 0.226529367 | 0.797320076 | 0 |
| 69 | 6.868268133 | 0.001069493 | 0.008 | 1.345940994 | 0.246152452 | 0.001 | 0.938419163 | 0.391449136 | 0.001 |
| 70 | 0.514825462 | 0.597698362 | 0.001 | 0.432408158 | 0.510899 | 0 | 0.411892753 | 0.662461634 | 0 |
| 71 | 2.609078603 | 0.073898085 | 0.003 | 0.346081647 | 0.556418061 | 0 | 0.464418022 | 0.62858077 | 0.001 |
| 72 | 0.110900903 | 0.895033936 | 0 | 9.56273E-05 | 0.992198827 | 0 | 0.002219934 | 0.997782531 | 0 |
| 73 | 0.477729459 | 0.620273491 | 0.001 | 0.677403437 | 0.410598123 | 0 | 1.63849518 | 0.194579898 | 0.002 |
| 74 | 2.981017567 | 0.051007429 | 0.004 | 0.576942392 | 0.447618993 | 0 | 1.119081628 | 0.32682089 | 0.001 |
| 75 | 1.439986866 | 0.237220738 | 0.002 | 6.017811351 | 0.014262359 | 0.004 | 1.275530114 | 0.27955097 | 0.002 |
| 76 | 0.211967603 | 0.80901236 | 0 | 1.052729327 | 0.30502582 | 0.001 | 3.574939834 | 0.028228685 | 0.004 |
| 77 | 0.013649306 | 0.986443532 | 0 | 0.561144328 | 0.453903545 | 0 | 0.742218248 | 0.476211479 | 0.001 |
| 78 | 1.773615114 | 0.170033342 | 0.002 | 4.095200402 | 0.043161857 | 0.002 | 1.074724419 | 0.34162448 | 0.001 |
| 79 | 8.619394735 | 0.000188609 | 0.01 | 0.025199993 | 0.873888661 | 0 | 0.459013899 | 0.631985033 | 0.001 |
| 80 | 2.940920632 | 0.053086812 | 0.003 | 2.557816014 | 0.109936583 | 0.002 | 0.323448501 | 0.723693906 | 0 |
| 81 | 3.795066129 | 0.022672799 | 0.004 | 10.18054361 | 0.0014453 | 0.006 | 0.208897838 | 0.811499035 | 0 |
| 82 | 2.697084189 | 0.067691196 | 0.003 | 13.52840001 | 0.000242319 | 0.008 | 0.620278386 | 0.537916802 | 0.001 |
| 83 | 3.991811272 | 0.018640209 | 0.005 | 4.745487089 | 0.029512709 | 0.003 | 1.317323893 | 0.268125386 | 0.002 |
| 84 | 2.86097206 | 0.057489609 | 0.003 | 3.031814394 | 0.081828207 | 0.002 | 0.528906148 | 0.589346443 | 0.001 |
| 85 | 0.385516254 | 0.680159094 | 0 | 0.570816633 | 0.450039626 | 0 | 1.224604029 | 0.294134067 | 0.001 |
| 86 | 0.270866813 | 0.762751102 | 0 | 0.726832883 | 0.394032221 | 0 | 2.03571534 | 0.130906377 | 0.002 |
| 87 | 2.320900941 | 0.098497222 | 0.003 | 1.615217137 | 0.203934476 | 0.001 | 0.817330173 | 0.441783173 | 0.001 |
| 88 | 5.635552758 | 0.003635941 | 0.007 | 0.073511346 | 0.786324268 | 0 | 0.878144247 | 0.41574244 | 0.001 |
| 89 | 1.034099854 | 0.355770613 | 0.001 | 0.026502596 | 0.870698334 | 0 | 1.328202177 | 0.265228948 | 0.002 |
| 90 | 0.680484509 | 0.506509954 | 0.001 | 9.270659754 | 0.002364416 | 0.005 | 1.971548467 | 0.139560448 | 0.002 |
| 91 | 0.958394632 | 0.383715909 | 0.001 | 5.202612364 | 0.022676641 | 0.003 | 0.455720396 | 0.634068782 | 0.001 |
| 92 | 4.92245845 | 0.007385698 | 0.006 | 2.069164875 | 0.150488525 | 0.001 | 0.197544213 | 0.820762757 | 0 |
| 93 | 0.738743896 | 0.477867433 | 0.001 | 1.572839553 | 0.209968148 | 0.001 | 0.416059867 | 0.659708167 | 0 |
| 94 | 7.516230348 | 0.000562524 | 0.009 | 0.248323676 | 0.6183226 | 0 | 0.70559246 | 0.493960993 | 0.001 |
| 95 | 2.571072135 | 0.076751852 | 0.003 | 2.187634156 | 0.139308785 | 0.001 | 0.831342131 | 0.435642027 | 0.001 |
| 96 | 0.509476071 | 0.600902309 | 0.001 | 2.43182781 | 0.119080983 | 0.001 | 0.529657949 | 0.588903815 | 0.001 |
| 97 | 5.287222328 | 0.005139557 | 0.006 | 2.900408165 | 0.088740561 | 0.002 | 0.639414581 | 0.527728491 | 0.001 |
| 98 | 3.173821075 | 0.042092339 | 0.004 | 4.967072543 | 0.025965102 | 0.003 | 0.347161514 | 0.706741445 | 0 |
| 99 | 3.328662326 | 0.036075595 | 0.004 | 0.723370083 | 0.395160877 | 0 | 0.017372931 | 0.982777283 | 0 |
| 100 | 0.074975888 | 0.927768935 | 0 | 4.619498951 | 0.03175175 | 0.003 | 0.001593724 | 0.998407547 | 0 |
| 101 | 1.034904203 | 0.355484913 | 0.001 | 2.901480957 | 0.088681632 | 0.002 | 1.647925612 | 0.192757071 | 0.002 |
| 102 | 0.553654703 | 0.574949062 | 0.001 | 0.162718659 | 0.686716657 | 0 | 0.151709038 | 0.859249922 | 0 |
| 103 | 1.491132509 | 0.225412891 | 0.002 | 7.114895881 | 0.007717425 | 0.004 | 0.678084845 | 0.507725891 | 0.001 |
| 104 | 4.453240117 | 0.011777384 | 0.005 | 13.18096945 | 0.000291213 | 0.008 | 0.754233926 | 0.470528705 | 0.001 |
| 105 | 3.172449975 | 0.042149876 | 0.004 | 4.095655145 | 0.043150277 | 0.002 | 1.692973343 | 0.184282818 | 0.002 |
| 106 | 3.120226191 | 0.044401011 | 0.004 | 0.297620179 | 0.585450041 | 0 | 1.325195735 | 0.266026293 | 0.002 |
| 107 | 0.377676132 | 0.685510163 | 0 | 1.855466703 | 0.173330012 | 0.001 | 2.525584039 | 0.080312812 | 0.003 |
| 108 | 4.118945287 | 0.016424807 | 0.005 | 1.96333022 | 0.161340773 | 0.001 | 0.893695714 | 0.4093337 | 0.001 |
| 109 | 0.473686315 | 0.622785009 | 0.001 | 3.02756514 | 0.082042383 | 0.002 | 2.489805661 | 0.083229506 | 0.003 |
| 110 | 2.951926343 | 0.05250776 | 0.003 | 1.424298602 | 0.232864278 | 0.001 | 1.011315739 | 0.363959579 | 0.001 |
| 111 | 0.838167401 | 0.432681685 | 0.001 | 1.000377981 | 0.317361911 | 0.001 | 0.620029609 | 0.538050542 | 0.001 |
| 112 | 0.001381307 | 0.998619647 | 0 | 2.158225111 | 0.141994083 | 0.001 | 0.052318584 | 0.949028008 | 0 |
| 113 | 0.123704424 | 0.883648967 | 0 | 0.399570944 | 0.527396222 | 0 | 1.086895828 | 0.337496866 | 0.001 |
| 114 | 7.320746501 | 0.000682812 | 0.009 | 0.024792527 | 0.874903909 | 0 | 0.399217783 | 0.670907684 | 0 |
| 115 | 6.367257242 | 0.001758237 | 0.007 | 3.963458412 | 0.046658496 | 0.002 | 0.099134597 | 0.905626059 | 0 |
| 116 | 0.855072272 | 0.425435916 | 0.001 | 1.025289082 | 0.311412303 | 0.001 | 0.81608831 | 0.442331618 | 0.001 |
| 117 | 2.87699758 | 0.056578712 | 0.003 | 0.06373978 | 0.80071045 | 0 | 0.822804815 | 0.439373503 | 0.001 |
| 118 | 0.68565864 | 0.503898074 | 0.001 | 5.993545185 | 0.014459144 | 0.004 | 0.763227943 | 0.46631949 | 0.001 |
| 119 | 0.380370333 | 0.68366657 | 0 | 5.186529974 | 0.022886776 | 0.003 | 0.899988182 | 0.406768775 | 0.001 |
| 120 | 7.550634116 | 0.000543665 | 0.009 | 1.535271318 | 0.215494681 | 0.001 | 0.592198642 | 0.553224322 | 0.001 |
| 121 | 7.672825556 | 0.000481656 | 0.009 | 10.25695087 | 0.001386995 | 0.006 | 0.139590463 | 0.869724345 | 0 |
| 122 | 1.124964825 | 0.324906311 | 0.001 | 0.762108643 | 0.382793159 | 0 | 0.793529387 | 0.452413858 | 0.001 |
| 123 | 5.944461675 | 0.002675286 | 0.007 | 5.027542892 | 0.025076205 | 0.003 | 0.457416592 | 0.632994767 | 0.001 |
| 124 | 2.599800272 | 0.074584805 | 0.003 | 0.933260894 | 0.334154908 | 0.001 | 0.899280452 | 0.407056454 | 0.001 |
| 125 | 1.895001751 | 0.150636587 | 0.002 | 2.871962607 | 0.090318689 | 0.002 | 0.344512826 | 0.708615097 | 0 |
| 126 | 2.056827973 | 0.128178092 | 0.002 | 0.141490546 | 0.706850784 | 0 | 0.733906094 | 0.480182845 | 0.001 |
| 127 | 0.322869574 | 0.724112833 | 0 | 8.813828508 | 0.003031544 | 0.005 | 0.793728838 | 0.452323717 | 0.001 |
| 128 | 0.798211193 | 0.450302674 | 0.001 | 0.420830131 | 0.516611045 | 0 | 1.1683052 | 0.31114377 | 0.001 |
| 129 | 0.950101237 | 0.386907838 | 0.001 | 0.011512245 | 0.914567553 | 0 | 2.270205399 | 0.103605131 | 0.003 |
| 130 | 0.412552538 | 0.662024908 | 0 | 4.234154782 | 0.039771074 | 0.002 | 1.393311266 | 0.248536261 | 0.002 |
| 131 | 0.792123136 | 0.453049918 | 0.001 | 0.097203727 | 0.755249798 | 0 | 2.296865699 | 0.100886714 | 0.003 |
| 132 | 3.33917145 | 0.035699928 | 0.004 | 0.394735331 | 0.529905448 | 0 | 0.611931999 | 0.542421961 | 0.001 |
| 133 | 3.689423123 | 0.025187409 | 0.004 | 0.465089331 | 0.495348254 | 0 | 0.8085823 | 0.445661051 | 0.001 |
| 134 | 0.862449582 | 0.422312047 | 0.001 | 6.886091537 | 0.008764945 | 0.004 | 0.157769829 | 0.854058884 | 0 |
| 135 | 1.759687374 | 0.172413093 | 0.002 | 5.239279269 | 0.022204995 | 0.003 | 0.875153636 | 0.416986336 | 0.001 |
| 136 | 0.346621461 | 0.707123069 | 0 | 2.58514396 | 0.108056949 | 0.002 | 0.618971775 | 0.538619595 | 0.001 |
| 137 | 4.430281435 | 0.012049462 | 0.005 | 0.930663167 | 0.334828355 | 0.001 | 4.795460655 | 0.008379756 | 0.006 |
| 138 | 3.541586978 | 0.029181998 | 0.004 | 4.586313822 | 0.032370534 | 0.003 | 1.60438807 | 0.201317811 | 0.002 |
| 139 | 0.617484652 | 0.539420599 | 0.001 | 2.684962127 | 0.101485334 | 0.002 | 3.707112185 | 0.024747688 | 0.004 |
| 140 | 0.533315195 | 0.586755328 | 0.001 | 0.163674981 | 0.685846419 | 0 | 0.56962825 | 0.565844037 | 0.001 |
| 141 | 2.580523263 | 0.076032056 | 0.003 | 0.439982436 | 0.507221184 | 0 | 0.151099307 | 0.8597739 | 0 |
| 142 | 2.499650369 | 0.082416539 | 0.003 | 0.293087316 | 0.588320053 | 0 | 0.384860865 | 0.680604807 | 0 |
| 143 | 5.228693293 | 0.00544738 | 0.006 | 0.174238104 | 0.676425309 | 0 | 1.31224392 | 0.2694888 | 0.002 |
| 144 | 1.796203244 | 0.166243556 | 0.002 | 15.4366282 | 8.87466E-05 | 0.009 | 1.68392892 | 0.185953764 | 0.002 |
| 145 | 0.249688806 | 0.779071845 | 0 | 6.774318927 | 0.009328568 | 0.004 | 0.304094364 | 0.737831509 | 0 |
| 146 | 1.571774687 | 0.207979009 | 0.002 | 1.434852328 | 0.231141737 | 0.001 | 0.989231595 | 0.372077032 | 0.001 |
| 147 | 1.949790687 | 0.142623082 | 0.002 | 4.794163815 | 0.028692314 | 0.003 | 0.315599088 | 0.729394672 | 0 |
| 148 | 0.64856287 | 0.522926328 | 0.001 | 0.242089395 | 0.622764852 | 0 | 1.274789429 | 0.279757794 | 0.002 |
| 149 | 1.493541463 | 0.224871488 | 0.002 | 0.024166601 | 0.876480276 | 0 | 0.687393356 | 0.503025419 | 0.001 |
| 150 | 6.615040582 | 0.001374943 | 0.008 | 0.018007631 | 0.893266284 | 0 | 1.075089661 | 0.341499885 | 0.001 |
| 151 | 6.17889936 | 0.002119721 | 0.007 | 2.970378234 | 0.084984495 | 0.002 | 0.419273009 | 0.657592874 | 0 |
| 152 | 2.593053626 | 0.075088156 | 0.003 | 0.783617221 | 0.376162775 | 0 | 1.635421023 | 0.19517783 | 0.002 |
| 153 | 4.732901623 | 0.00891761 | 0.006 | 6.760441405 | 0.009401094 | 0.004 | 0.339353762 | 0.712278852 | 0 |
| 154 | 2.929337154 | 0.053703169 | 0.003 | 0.3132237 | 0.575782689 | 0 | 0.137169394 | 0.871832215 | 0 |
| 155 | 2.1422257 | 0.117711189 | 0.003 | 3.162973811 | 0.075505161 | 0.002 | 0.929896722 | 0.394795787 | 0.001 |
| 156 | 2.844847092 | 0.058420979 | 0.003 | 3.055656135 | 0.080637651 | 0.002 | 0.118893654 | 0.887909629 | 0 |
| 157 | 0.754002224 | 0.470637644 | 0.001 | 0.0352595 | 0.85107542 | 0 | 1.082721208 | 0.338906922 | 0.001 |
| 158 | 0.865101277 | 0.421194826 | 0.001 | 1.425644211 | 0.232643792 | 0.001 | 0.091847172 | 0.912249098 | 0 |
| 159 | 2.033086715 | 0.131250107 | 0.002 | 3.014242953 | 0.082717799 | 0.002 | 0.331061575 | 0.718207401 | 0 |
| 160 | 4.729387707 | 0.008948826 | 0.006 | 0.180887288 | 0.670665846 | 0 | 0.065536124 | 0.936567589 | 0 |
| 161 | 3.931609773 | 0.019791296 | 0.005 | 1.853138733 | 0.173599822 | 0.001 | 0.415307093 | 0.660204722 | 0 |
| 162 | 4.599919765 | 0.010178536 | 0.005 | 7.177706034 | 0.007452935 | 0.004 | 0.579517172 | 0.560279782 | 0.001 |
| 163 | 1.995872537 | 0.136214465 | 0.002 | 0.139596296 | 0.708729432 | 0 | 1.131040395 | 0.322940915 | 0.001 |
| 164 | 3.130958411 | 0.043928771 | 0.004 | 7.699083347 | 0.005585553 | 0.005 | 1.774778622 | 0.169836035 | 0.002 |
| 165 | 0.49761346 | 0.608068741 | 0.001 | 2.372214631 | 0.123698486 | 0.001 | 3.500763919 | 0.030392809 | 0.004 |
| 166 | 7.690371742 | 0.000473353 | 0.009 | 0.250849129 | 0.616542881 | 0 | 0.816193283 | 0.442285233 | 0.001 |
| 167 | 11.62091247 | 9.71427E-06 | 0.014 | 0.006615308 | 0.93518551 | 0 | 0.405809939 | 0.666501589 | 0 |
| 168 | 0.126205169 | 0.881442272 | 0 | 0.423439585 | 0.515313977 | 0 | 3.50543065 | 0.030251886 | 0.004 |
| 169 | 2.660574743 | 0.070200152 | 0.003 | 2.135611899 | 0.144098487 | 0.001 | 2.973252097 | 0.051403669 | 0.003 |
| 170 | 1.013410781 | 0.363198775 | 0.001 | 0.888239593 | 0.346089192 | 0.001 | 0.718810338 | 0.487480251 | 0.001 |
| 171 | 0.654691914 | 0.51973354 | 0.001 | 0.514511776 | 0.473291131 | 0 | 1.804247267 | 0.164914467 | 0.002 |
| 172 | 1.083003324 | 0.338811447 | 0.001 | 1.616463678 | 0.203760119 | 0.001 | 0.639297786 | 0.527790084 | 0.001 |
| 173 | 5.493900836 | 0.004185371 | 0.006 | 1.282817459 | 0.257536118 | 0.001 | 1.805233673 | 0.16475222 | 0.002 |
| 174 | 2.510584073 | 0.081522958 | 0.003 | 6.014213368 | 0.014291362 | 0.004 | 1.420238551 | 0.241943957 | 0.002 |
| 175 | 0.475173523 | 0.621860004 | 0.001 | 1.65388848 | 0.198606264 | 0.001 | 0.095123867 | 0.909265156 | 0 |
| 176 | 3.804760821 | 0.022455027 | 0.004 | 0.474003369 | 0.491245445 | 0 | 1.088559939 | 0.33693642 | 0.001 |
| 177 | 0.157912458 | 0.853937102 | 0 | 3.605832923 | 0.057746984 | 0.002 | 0.943074691 | 0.389632981 | 0.001 |
| 178 | 3.666087478 | 0.025779484 | 0.004 | 7.083893166 | 0.007851498 | 0.004 | 0.893579125 | 0.409381377 | 0.001 |
| 179 | 2.977141449 | 0.051204828 | 0.004 | 1.356174585 | 0.244365535 | 0.001 | 0.282041703 | 0.75427764 | 0 |
| 180 | 0.81437238 | 0.443090549 | 0.001 | 10.53171634 | 0.001196322 | 0.006 | 0.766842255 | 0.464638623 | 0.001 |
| 181 | 2.654451538 | 0.070629968 | 0.003 | 5.771113007 | 0.016398758 | 0.003 | 0.430458082 | 0.650282278 | 0.001 |
| 182 | 1.885072544 | 0.152136375 | 0.002 | 1.050270802 | 0.305591046 | 0.001 | 1.450949657 | 0.234638722 | 0.002 |
| 183 | 1.506452227 | 0.221991953 | 0.002 | 3.608709645 | 0.057647394 | 0.002 | 1.262172219 | 0.283304574 | 0.001 |
| 184 | 5.209679569 | 0.005551299 | 0.006 | 6.034736636 | 0.01412674 | 0.004 | 0.506480563 | 0.602703933 | 0.001 |
| 185 | 3.501439 | 0.030372383 | 0.004 | 0.388218254 | 0.533321325 | 0 | 1.23934859 | 0.289835212 | 0.001 |
| 186 | 6.778018439 | 0.00116966 | 0.008 | 3.568081624 | 0.059070998 | 0.002 | 0.21358238 | 0.807707367 | 0 |
| 187 | 15.95084474 | 1.37106E-07 | 0.018 | 0.785192147 | 0.375683656 | 0 | 0.263841944 | 0.76812649 | 0 |
| 188 | 1.894089955 | 0.150773692 | 0.002 | 0.83549682 | 0.360817946 | 0 | 2.10654836 | 0.121975737 | 0.002 |
| 189 | 1.219217228 | 0.295720489 | 0.001 | 0.009678625 | 0.921642191 | 0 | 2.908839225 | 0.054811473 | 0.003 |
| 190 | 1.854954548 | 0.156777715 | 0.002 | 4.97342859 | 0.025870149 | 0.003 | 7.475725832 | 0.000585568 | 0.009 |
| 191 | 0.421508346 | 0.656125302 | 0 | 3.165761525 | 0.075376617 | 0.002 | 5.335722223 | 0.004897717 | 0.006 |
| 192 | 1.496992713 | 0.224098101 | 0.002 | 6.305070771 | 0.01213177 | 0.004 | 2.019392836 | 0.133055444 | 0.002 |
| 193 | 0.553985938 | 0.574758775 | 0.001 | 12.81532327 | 0.000353482 | 0.008 | 0.005234857 | 0.994778837 | 0 |
| 194 | 0.185809845 | 0.830448414 | 0 | 12.33545806 | 0.000456091 | 0.007 | 0.669178244 | 0.512264576 | 0.001 |
| 195 | 4.613557721 | 0.010041405 | 0.005 | 13.14061086 | 0.000297504 | 0.008 | 1.822932111 | 0.161868137 | 0.002 |
| 196 | 6.404919008 | 0.001693727 | 0.008 | 17.10126664 | 3.71736E-05 | 0.01 | 0.5015452 | 0.60568407 | 0.001 |
| 197 | 17.65605603 | 2.57572E-08 | 0.02 | 9.525598863 | 0.002059074 | 0.006 | 0.291851868 | 0.746916711 | 0 |
| 198 | 3.762146059 | 0.023428185 | 0.004 | 3.687793483 | 0.054979809 | 0.002 | 0.395856641 | 0.673165433 | 0 |
| 199 | 2.562458786 | 0.077413783 | 0.003 | 10.83208255 | 0.001018069 | 0.006 | 0.20421763 | 0.81530499 | 0 |
| 200 | 5.754991982 | 0.003229174 | 0.007 | 12.16531655 | 0.000499303 | 0.007 | 0.197586552 | 0.820728015 | 0 |
| 201 | 1.380975083 | 0.251616164 | 0.002 | 0.802106103 | 0.370591666 | 0 | 0.200775331 | 0.818115677 | 0 |
| 202 | 2.567917445 | 0.076993628 | 0.003 | 0.194917915 | 0.658910962 | 0 | 0.981551209 | 0.374942383 | 0.001 |
| 203 | 0.695292801 | 0.499070666 | 0.001 | 0.712742038 | 0.398654228 | 0 | 0.28518939 | 0.751907936 | 0 |
| 204 | 1.496377913 | 0.224235675 | 0.002 | 2.17338681 | 0.140602494 | 0.001 | 0.921827407 | 0.3979909 | 0.001 |
| 205 | 1.465241087 | 0.231314928 | 0.002 | 0.453302279 | 0.500862753 | 0 | 1.3980953 | 0.247352041 | 0.002 |
| 206 | 4.194050696 | 0.015241991 | 0.005 | 0.000554396 | 0.981217831 | 0 | 1.01673868 | 0.361993537 | 0.001 |
| 207 | 11.36228188 | 1.2538E-05 | 0.013 | 0.000205237 | 0.988571507 | 0 | 0.52685429 | 0.590556185 | 0.001 |
| 208 | 1.88248557 | 0.152529582 | 0.002 | 1.89711094 | 0.168584008 | 0.001 | 0.284459822 | 0.75245652 | 0 |
| 209 | 1.30928452 | 0.27028627 | 0.002 | 0.641818742 | 0.423165321 | 0 | 0.88526732 | 0.412794656 | 0.001 |
| 210 | 1.402674798 | 0.246223744 | 0.002 | 0.000368597 | 0.984684719 | 0 | 0.964709706 | 0.38130308 | 0.001 |
| 211 | 9.480360126 | 8.04643E-05 | 0.011 | 0.000859371 | 0.976616791 | 0 | 1.771992129 | 0.170308951 | 0.002 |
| 212 | 6.093528633 | 0.002307215 | 0.007 | 0.169053761 | 0.681006141 | 0 | 1.721290005 | 0.179147937 | 0.002 |
| 213 | 2.193628345 | 0.111828076 | 0.003 | 0.851692559 | 0.356205385 | 0.001 | 0.923858107 | 0.397184398 | 0.001 |
| 214 | 0.98700819 | 0.372904264 | 0.001 | 0.447780503 | 0.503482032 | 0 | 5.191692696 | 0.005651433 | 0.006 |
| 215 | 0.910357565 | 0.402577084 | 0.001 | 7.063669322 | 0.00794024 | 0.004 | 1.012837507 | 0.363406798 | 0.001 |
| 216 | 0.411351154 | 0.662820345 | 0 | 0.083224303 | 0.773009866 | 0 | 0.13096336 | 0.877258797 | 0 |
| 217 | 0.56487632 | 0.568537479 | 0.001 | 5.40362E-05 | 0.994135725 | 0 | 3.637278889 | 0.026529675 | 0.004 |
| 218 | 0.342289311 | 0.710191827 | 0 | 0.403494389 | 0.525375893 | 0 | 0.259028509 | 0.771831583 | 0 |
| 219 | 7.19628968 | 0.00077249 | 0.008 | 0.795089604 | 0.372692198 | 0 | 0.242599782 | 0.78461271 | 0 |
| 220 | 5.437579136 | 0.004426264 | 0.006 | 0.017000538 | 0.896276436 | 0 | 0.939008625 | 0.391218715 | 0.001 |
| 221 | 0.476048895 | 0.621316189 | 0.001 | 0.028447741 | 0.866080615 | 0 | 0.97707289 | 0.376623312 | 0.001 |
| 222 | 2.900065614 | 0.055292826 | 0.003 | 0.445986024 | 0.504338287 | 0 | 0.377678051 | 0.685508848 | 0 |
| 223 | 1.092257274 | 0.335694549 | 0.001 | 2.871781982 | 0.090328807 | 0.002 | 1.244552238 | 0.288333125 | 0.001 |
| 224 | 1.264126654 | 0.282752237 | 0.001 | 0.001942288 | 0.964852688 | 0 | 4.360299016 | 0.012918251 | 0.005 |
| 225 | 6.576698815 | 0.001428262 | 0.008 | 2.172314938 | 0.140700369 | 0.001 | 0.085879141 | 0.917709133 | 0 |
| 226 | 3.516628144 | 0.029916413 | 0.004 | 0.169335201 | 0.680755368 | 0 | 0.046239306 | 0.954814648 | 0 |
| 227 | 0.29725852 | 0.742890686 | 0 | 3.163761877 | 0.075468799 | 0.002 | 1.490012408 | 0.225665074 | 0.002 |
| 228 | 4.096863012 | 0.016789749 | 0.005 | 0.972869456 | 0.324106441 | 0.001 | 1.574064893 | 0.207504121 | 0.002 |
| 229 | 14.46190879 | 5.91956E-07 | 0.017 | 0.438248668 | 0.508059005 | 0 | 0.386670033 | 0.679375151 | 0 |
| 230 | 0.488016811 | 0.613928827 | 0.001 | 0.106765219 | 0.743897743 | 0 | 1.078108714 | 0.340471738 | 0.001 |
| 231 | 0.034193163 | 0.966385484 | 0 | 5.85902181 | 0.015601969 | 0.003 | 0.896210869 | 0.40830654 | 0.001 |
| 232 | 0.171591267 | 0.842338027 | 0 | 2.01781531 | 0.1556467 | 0.001 | 0.801825621 | 0.448679556 | 0.001 |
| 233 | 4.53198717 | 0.01089005 | 0.005 | 2.381819281 | 0.122941292 | 0.001 | 0.334432447 | 0.715791439 | 0 |
| 234 | 5.164662152 | 0.005805324 | 0.006 | 1.219715424 | 0.269573351 | 0.001 | 1.007009167 | 0.365528503 | 0.001 |
| 235 | 0.214658245 | 0.80683907 | 0 | 3.254408582 | 0.071409204 | 0.002 | 0.259923034 | 0.771141681 | 0 |
| 236 | 6.130903953 | 0.002223169 | 0.007 | 4.867868389 | 0.027495045 | 0.003 | 0.742554521 | 0.47605151 | 0.001 |
| 237 | 1.290746059 | 0.275335873 | 0.002 | 1.41840223 | 0.233833416 | 0.001 | 0.996538495 | 0.369371371 | 0.001 |
| 238 | 1.214951862 | 0.296982721 | 0.001 | 3.089164386 | 0.078995876 | 0.002 | 1.590144659 | 0.204200307 | 0.002 |
| 239 | 1.811104687 | 0.16378984 | 0.002 | 0.871285587 | 0.350732673 | 0.001 | 0.750393974 | 0.472337377 | 0.001 |
| 240 | 1.910583877 | 0.148312721 | 0.002 | 6.835134869 | 0.009017438 | 0.004 | 0.938537966 | 0.391402684 | 0.001 |
| 241 | 0.634226109 | 0.530471643 | 0.001 | 4.168763965 | 0.04133045 | 0.002 | 0.078067498 | 0.924905323 | 0 |
| 242 | 5.277867878 | 0.005187559 | 0.006 | 1.663871091 | 0.197257529 | 0.001 | 0.672038671 | 0.51080253 | 0.001 |
| 243 | 0.846375891 | 0.429148058 | 0.001 | 3.365104437 | 0.066766659 | 0.002 | 0.7535854 | 0.470833683 | 0.001 |
| 244 | 6.271181791 | 0.001934164 | 0.007 | 0.683039773 | 0.408658339 | 0 | 0.322134199 | 0.72464532 | 0 |
| 245 | 4.299240556 | 0.013727335 | 0.005 | 1.027144679 | 0.310974976 | 0.001 | 2.731344606 | 0.065418504 | 0.003 |
| 246 | 11.99920903 | 6.68925E-06 | 0.014 | 0.169175175 | 0.680897927 | 0 | 0.44715759 | 0.639518584 | 0.001 |
| 247 | 6.971574377 | 0.000965331 | 0.008 | 1.797658484 | 0.180175591 | 0.001 | 2.970374289 | 0.051551293 | 0.003 |
| 248 | 12.8338402 | 2.93859E-06 | 0.015 | 0.004504042 | 0.946500333 | 0 | 0.457332094 | 0.633048227 | 0.001 |
| 249 | 1.685406567 | 0.185679737 | 0.002 | 0.506902793 | 0.476580733 | 0 | 1.625200477 | 0.197179012 | 0.002 |
| 250 | 0.605844139 | 0.545731835 | 0.001 | 0.249283188 | 0.617645094 | 0 | 3.362921621 | 0.034865301 | 0.004 |
| 251 | 3.786813409 | 0.022859845 | 0.004 | 0.200325466 | 0.654515493 | 0 | 0.029455129 | 0.970974943 | 0 |
| 252 | 4.869422448 | 0.007785606 | 0.006 | 0.047276486 | 0.827898217 | 0 | 2.20550436 | 0.110511258 | 0.003 |
| 253 | 4.39126947 | 0.012526293 | 0.005 | 1.981841372 | 0.15938003 | 0.001 | 1.213098929 | 0.297532731 | 0.001 |
| 254 | 0.584422939 | 0.557539789 | 0.001 | 1.202322554 | 0.273013819 | 0.001 | 0.387550144 | 0.678777761 | 0 |
| 255 | 0.651610432 | 0.521336322 | 0.001 | 0.016542733 | 0.897674749 | 0 | 0.251911265 | 0.777342824 | 0 |
| 256 | 2.430086817 | 0.088336009 | 0.003 | 0.757194753 | 0.384331131 | 0 | 0.689236624 | 0.502099814 | 0.001 |
| 257 | 0.970040594 | 0.379278111 | 0.001 | 0.264891246 | 0.606846683 | 0 | 0.038125674 | 0.962592786 | 0 |
| 258 | 0.935392654 | 0.392634341 | 0.001 | 1.184871651 | 0.276521286 | 0.001 | 0.060914805 | 0.940905458 | 0 |
| 259 | 1.153415911 | 0.315804704 | 0.001 | 0.635355249 | 0.425509454 | 0 | 1.023558356 | 0.359536206 | 0.001 |
| 260 | 0.674972754 | 0.509307176 | 0.001 | 0.098126165 | 0.754128566 | 0 | 0.838549508 | 0.432516549 | 0.001 |
| 261 | 0.607895982 | 0.544614028 | 0.001 | 0.043909327 | 0.834047596 | 0 | 0.017275391 | 0.982873146 | 0 |
| 262 | 0.501050274 | 0.605983736 | 0.001 | 0.315920658 | 0.574143916 | 0 | 0.650060712 | 0.522144253 | 0.001 |
| 263 | 0.867188952 | 0.420317322 | 0.001 | 0.181228497 | 0.670373686 | 0 | 0.961203261 | 0.382640922 | 0.001 |
| 264 | 0.521838242 | 0.593524043 | 0.001 | 0.073043266 | 0.786989175 | 0 | 0.289088844 | 0.748982604 | 0 |
| 265 | 0.049125456 | 0.952063037 | 0 | 0.582041325 | 0.445619521 | 0 | 0.154802164 | 0.85659674 | 0 |
| 266 | 0.868800251 | 0.419641302 | 0.001 | 1.163935069 | 0.280804524 | 0.001 | 0.518856917 | 0.595295081 | 0.001 |
| 267 | 0.270696084 | 0.762881295 | 0 | 3.022460186 | 0.08230049 | 0.002 | 0.385769877 | 0.67998669 | 0 |
| 268 | 0.84279273 | 0.430686985 | 0.001 | 0.017472152 | 0.894855825 | 0 | 0.952648214 | 0.385924749 | 0.001 |
| 269 | 0.097494349 | 0.907112557 | 0 | 1.416753815 | 0.234105225 | 0.001 | 0.313250469 | 0.731109119 | 0 |
| 270 | 0.825186739 | 0.438329209 | 0.001 | 0.731117879 | 0.392641992 | 0 | 1.462352904 | 0.231982817 | 0.002 |
| 271 | 0.723093837 | 0.485398364 | 0.001 | 0.003398666 | 0.953518076 | 0 | 0.062436104 | 0.939475251 | 0 |
| 272 | 3.13409178 | 0.043791847 | 0.004 | 3.626956954 | 0.057019921 | 0.002 | 0.774508584 | 0.461093383 | 0.001 |
| 273 | 0.922733669 | 0.397630771 | 0.001 | 1.236733443 | 0.266259326 | 0.001 | 0.065565948 | 0.936539658 | 0 |
| 274 | 10.45389087 | 3.07413E-05 | 0.012 | 1.981837065 | 0.159380483 | 0.001 | 1.428523838 | 0.239951001 | 0.002 |
| 275 | 0.499137766 | 0.607143108 | 0.001 | 0.485777778 | 0.485912443 | 0 | 1.408242076 | 0.244859015 | 0.002 |
| 276 | 7.277266062 | 0.000712891 | 0.009 | 4.844162789 | 0.02787434 | 0.003 | 2.94597199 | 0.052820252 | 0.003 |
| 277 | 4.709130458 | 0.009130928 | 0.006 | 3.35105105 | 0.067337713 | 0.002 | 0.110580517 | 0.895320701 | 0 |
| 278 | 6.203906214 | 0.002067746 | 0.007 | 2.745907079 | 0.097688906 | 0.002 | 0.067639541 | 0.934599821 | 0 |
| 279 | 0.701961881 | 0.495756123 | 0.001 | 0.04975827 | 0.823511331 | 0 | 2.767106307 | 0.063127668 | 0.003 |
| 280 | 0.36282121 | 0.695764858 | 0 | 2.962261894 | 0.085411225 | 0.002 | 0.167773063 | 0.845559746 | 0 |
| 281 | 0.907801425 | 0.403606337 | 0.001 | 4.925356333 | 0.026597371 | 0.003 | 0.129303559 | 0.878715858 | 0 |
| 282 | 1.753089331 | 0.173552073 | 0.002 | 5.186774035 | 0.022883572 | 0.003 | 0.944709384 | 0.388997279 | 0.001 |
| 283 | 5.031388144 | 0.006627662 | 0.006 | 0.096104649 | 0.756593386 | 0 | 0.154878359 | 0.856531486 | 0 |
| 284 | 3.140460984 | 0.043514837 | 0.004 | 0.744666382 | 0.388292309 | 0 | 0.683811027 | 0.50482919 | 0.001 |
| 285 | 0.037559816 | 0.963137607 | 0 | 3.793543661 | 0.051615611 | 0.002 | 0.41171823 | 0.662577202 | 0 |
| 286 | 4.835582146 | 0.008052029 | 0.006 | 5.034297483 | 0.024978897 | 0.003 | 2.580097332 | 0.076064349 | 0.003 |
| 287 | 2.392051343 | 0.091750721 | 0.003 | 3.133050907 | 0.076899885 | 0.002 | 0.071465241 | 0.931031446 | 0 |
| 288 | 2.921191086 | 0.054140907 | 0.003 | 2.539707203 | 0.111201923 | 0.001 | 0.534747173 | 0.585916237 | 0.001 |
| 289 | 1.711942718 | 0.180826916 | 0.002 | 1.610604987 | 0.20458112 | 0.001 | 0.641603288 | 0.526575582 | 0.001 |
| 290 | 9.187425053 | 0.000107507 | 0.011 | 7.706596659 | 0.005562459 | 0.005 | 1.849218994 | 0.157677532 | 0.002 |
| 291 | 3.48275887 | 0.030942702 | 0.004 | 0.56912161 | 0.450713032 | 0 | 2.001012796 | 0.135517724 | 0.002 |
| 292 | 1.578611323 | 0.206564603 | 0.002 | 2.69756738 | 0.100687083 | 0.002 | 3.291786482 | 0.037425372 | 0.004 |
| 293 | 4.123563619 | 0.016349492 | 0.005 | 1.289369386 | 0.256324825 | 0.001 | 0.056124644 | 0.945423045 | 0 |
| 294 | 1.76294075 | 0.17185424 | 0.002 | 4.071685212 | 0.04376514 | 0.002 | 0.46744698 | 0.62668075 | 0.001 |
| 295 | 0.932568369 | 0.393743595 | 0.001 | 11.65986097 | 0.000653697 | 0.007 | 0.683342067 | 0.5050658 | 0.001 |
| 296 | 0.259393734 | 0.771549829 | 0 | 3.709455798 | 0.054272205 | 0.002 | 0.445821934 | 0.640372882 | 0.001 |
| 297 | 2.301547165 | 0.100416793 | 0.003 | 3.2833443 | 0.070163181 | 0.002 | 0.783180614 | 0.457115698 | 0.001 |
| 298 | 5.57303069 | 0.003868934 | 0.007 | 1.56129529 | 0.211648277 | 0.001 | 0.763513261 | 0.466186579 | 0.001 |
| 299 | 1.784527039 | 0.168191884 | 0.002 | 2.069426515 | 0.150462744 | 0.001 | 1.966137728 | 0.140315859 | 0.002 |
| 300 | 1.257762364 | 0.284554801 | 0.001 | 1.362085389 | 0.243340649 | 0.001 | 1.131101393 | 0.322921243 | 0.001 |
| 301 | 4.2748452 | 0.014064608 | 0.005 | 2.485380047 | 0.115095304 | 0.001 | 1.692811204 | 0.18431264 | 0.002 |
| 302 | 0.71999565 | 0.486903267 | 0.001 | 0.920565464 | 0.337463462 | 0.001 | 0.261902315 | 0.769617353 | 0 |
| 303 | 0.468611283 | 0.625951931 | 0.001 | 7.231922776 | 0.007232096 | 0.004 | 2.085760012 | 0.12453156 | 0.002 |
| 304 | 2.01397079 | 0.133777113 | 0.002 | 3.49799499 | 0.061615508 | 0.002 | 0.383873188 | 0.681277051 | 0 |
| 305 | 0.090685381 | 0.913309442 | 0 | 12.33322055 | 0.000456634 | 0.007 | 0.229672466 | 0.794818627 | 0 |
| 306 | 4.51094597 | 0.011120375 | 0.005 | 10.08378237 | 0.001522717 | 0.006 | 0.695984682 | 0.498725771 | 0.001 |
| 307 | 1.620270638 | 0.198151605 | 0.002 | 1.283992839 | 0.257318299 | 0.001 | 1.721110893 | 0.179179962 | 0.002 |
| 308 | 2.190926893 | 0.112129801 | 0.003 | 2.517381435 | 0.11278405 | 0.001 | 0.912805735 | 0.401593769 | 0.001 |
| 309 | 4.060808657 | 0.01740313 | 0.005 | 3.435825647 | 0.063970439 | 0.002 | 0.919056781 | 0.399093913 | 0.001 |
| 310 | 4.201942031 | 0.015122773 | 0.005 | 0.388522821 | 0.533160804 | 0 | 1.606577951 | 0.200878264 | 0.002 |
| 311 | 1.445911718 | 0.235821772 | 0.002 | 0.359576505 | 0.548821722 | 0 | 0.546210791 | 0.579242104 | 0.001 |
| 312 | 6.236785358 | 0.002001346 | 0.007 | 0.000262762 | 0.987068824 | 0 | 0.569790787 | 0.565752136 | 0.001 |
| 313 | 0.016278493 | 0.983853439 | 0 | 3.443852152 | 0.063661075 | 0.002 | 1.032613552 | 0.356299143 | 0.001 |
| 314 | 0.013592221 | 0.986499844 | 0 | 0.758061644 | 0.384059171 | 0 | 3.048699535 | 0.047680827 | 0.004 |
| 315 | 0.355559149 | 0.700833779 | 0 | 0.226537905 | 0.634164262 | 0 | 0.075814923 | 0.9269909 | 0 |
| 316 | 1.116139838 | 0.327782473 | 0.001 | 0.624284686 | 0.429570099 | 0 | 0.541913956 | 0.581734762 | 0.001 |
| 317 | 1.98013612 | 0.138369849 | 0.002 | 2.921810656 | 0.087572903 | 0.002 | 0.625369783 | 0.535187016 | 0.001 |
| 318 | 0.053445289 | 0.947959402 | 0 | 1.367625616 | 0.242384775 | 0.001 | 0.978871859 | 0.375947168 | 0.001 |
| 319 | 9.118826001 | 0.000115056 | 0.011 | 4.19519471 | 0.040692556 | 0.002 | 0.09371055 | 0.910551002 | 0 |
| 320 | 4.680588731 | 0.009393817 | 0.005 | 1.204021076 | 0.272675422 | 0.001 | 0.184857407 | 0.831239568 | 0 |
| 321 | 1.386756401 | 0.250168047 | 0.002 | 2.507850581 | 0.113467003 | 0.001 | 0.799991038 | 0.449502672 | 0.001 |
| 322 | 1.460431255 | 0.232428266 | 0.002 | 8.237964043 | 0.004153238 | 0.005 | 0.276797521 | 0.758242288 | 0 |
| 323 | 3.603088416 | 0.027448427 | 0.004 | 2.959172034 | 0.085574289 | 0.002 | 2.003505682 | 0.135181109 | 0.002 |
| 324 | 0.260462741 | 0.77072573 | 0 | 8.058683472 | 0.004582622 | 0.005 | 0.110622202 | 0.895283386 | 0 |
| 325 | 10.31621752 | 3.52199E-05 | 0.012 | 2.536672202 | 0.111415557 | 0.001 | 0.631445447 | 0.531947654 | 0.001 |
| 326 | 0.147005392 | 0.863300334 | 0 | 1.722175818 | 0.189590993 | 0.001 | 2.673204735 | 0.069321848 | 0.003 |
| 327 | 5.854602971 | 0.002924997 | 0.007 | 1.959041856 | 0.161798923 | 0.001 | 0.524975571 | 0.591666027 | 0.001 |
| 328 | 2.981814377 | 0.050966944 | 0.004 | 2.007397003 | 0.156717565 | 0.001 | 0.293090013 | 0.745992811 | 0 |
| 329 | 8.056737982 | 0.000329261 | 0.009 | 0.189878178 | 0.663073547 | 0 | 0.359823626 | 0.697852709 | 0 |
| 330 | 4.278208329 | 0.014017623 | 0.005 | 1.586149391 | 0.208050641 | 0.001 | 0.132672557 | 0.875760903 | 0 |
| 331 | 9.211105383 | 0.000105018 | 0.011 | 0.135111733 | 0.713235719 | 0 | 0.632526833 | 0.531373152 | 0.001 |
| 332 | 4.066238005 | 0.017309347 | 0.005 | 0.9331107 | 0.334193795 | 0.001 | 2.909042732 | 0.054800358 | 0.003 |
| 333 | 0.659435448 | 0.517275907 | 0.001 | 0.019439651 | 0.889129983 | 0 | 2.830241857 | 0.059277602 | 0.003 |
| 334 | 2.435464687 | 0.087863578 | 0.003 | 11.99735783 | 0.00054602 | 0.007 | 0.277046149 | 0.758053854 | 0 |
| 335 | 1.965160445 | 0.140452737 | 0.002 | 2.088179967 | 0.148627774 | 0.001 | 1.751985331 | 0.173743385 | 0.002 |
| 336 | 1.951566044 | 0.14237068 | 0.002 | 0.347057148 | 0.555862303 | 0 | 1.147665062 | 0.317623604 | 0.001 |
| 337 | 0.289598558 | 0.748601065 | 0 | 0.090518471 | 0.763555981 | 0 | 0.858404524 | 0.424022043 | 0.001 |
| 338 | 2.163192832 | 0.115274944 | 0.003 | 3.512001413 | 0.06109782 | 0.002 | 0.253499194 | 0.776109806 | 0 |
| 339 | 4.16170709 | 0.015740532 | 0.005 | 0.024446571 | 0.875772629 | 0 | 0.137520785 | 0.871525964 | 0 |
| 340 | 0.968939991 | 0.379695298 | 0.001 | 5.024636448 | 0.025118196 | 0.003 | 2.030701581 | 0.131562778 | 0.002 |
| 341 | 1.373022615 | 0.253621837 | 0.002 | 10.97032259 | 0.000945313 | 0.006 | 0.924779093 | 0.396819163 | 0.001 |
| 342 | 1.097924943 | 0.333799767 | 0.001 | 5.880581082 | 0.015412729 | 0.003 | 0.181472909 | 0.834057052 | 0 |
| 343 | 3.142256379 | 0.043437069 | 0.004 | 2.904028504 | 0.088541865 | 0.002 | 0.052802492 | 0.948568905 | 0 |
| 344 | 1.444179353 | 0.236229958 | 0.002 | 3.445410945 | 0.06360118 | 0.002 | 0.038578652 | 0.962156871 | 0 |
| 345 | 4.841153356 | 0.008007548 | 0.006 | 0.058540972 | 0.80884632 | 0 | 3.40525595 | 0.033425739 | 0.004 |
| 346 | 0.741689176 | 0.476463276 | 0.001 | 6.693234165 | 0.009760597 | 0.004 | 0.187183559 | 0.829308648 | 0 |
| 347 | 1.891849198 | 0.151111163 | 0.002 | 0.05748852 | 0.810539404 | 0 | 0.429259194 | 0.651061965 | 0.001 |
| 348 | 0.999431607 | 0.368305537 | 0.001 | 1.091776887 | 0.296226919 | 0.001 | 0.521287596 | 0.593850754 | 0.001 |
| 349 | 1.733077452 | 0.177052884 | 0.002 | 0.190950994 | 0.662181985 | 0 | 0.620757495 | 0.537659331 | 0.001 |
| 350 | 1.757559614 | 0.172779575 | 0.002 | 0.352073275 | 0.553021051 | 0 | 0.073249022 | 0.929372311 | 0 |
| 351 | 2.76854225 | 0.06303738 | 0.003 | 0.057805376 | 0.810027965 | 0 | 0.601218094 | 0.548260458 | 0.001 |
| 352 | 0.351428432 | 0.703733499 | 0 | 0.102924437 | 0.748387597 | 0 | 0.38875822 | 0.677958616 | 0 |
| 353 | 0.37488355 | 0.687426329 | 0 | 2.162643179 | 0.141586986 | 0.001 | 1.229783174 | 0.292616834 | 0.001 |
| 354 | 2.72977698 | 0.065520806 | 0.003 | 1.203299169 | 0.272819184 | 0.001 | 0.499542246 | 0.606897725 | 0.001 |
| 355 | 0.639014953 | 0.527939269 | 0.001 | 0.094518125 | 0.758547798 | 0 | 1.659043691 | 0.190629981 | 0.002 |
| 356 | 0.112132347 | 0.893932576 | 0 | 14.34660309 | 0.000157364 | 0.008 | 2.059021129 | 0.127897965 | 0.002 |
| 357 | 2.737053023 | 0.065047326 | 0.003 | 9.73513907 | 0.001838264 | 0.006 | 0.744337258 | 0.475204334 | 0.001 |
| 358 | 2.061669939 | 0.127560456 | 0.002 | 11.13637588 | 0.000864835 | 0.007 | 1.075578349 | 0.34133325 | 0.001 |
| 359 | 4.587179664 | 0.010308333 | 0.005 | 0.423640885 | 0.515214155 | 0 | 0.760651077 | 0.4675216 | 0.001 |
| 360 | 0.924940596 | 0.396755151 | 0.001 | 0.412186438 | 0.520948704 | 0 | 0.48443466 | 0.61613069 | 0.001 |
| 361 | 0.24529749 | 0.782499515 | 0 | 0.042189369 | 0.837283901 | 0 | 0.7927171 | 0.452781154 | 0.001 |
| 362 | 2.486933704 | 0.083468179 | 0.003 | 4.922385354 | 0.026643007 | 0.003 | 0.462029982 | 0.630082817 | 0.001 |
| 363 | 0.249489406 | 0.779227162 | 0 | 0.067190926 | 0.795503072 | 0 | 0.519464377 | 0.594933795 | 0.001 |
| 364 | 6.624481065 | 0.001362124 | 0.008 | 0.259584071 | 0.610472411 | 0 | 0.537769664 | 0.584149101 | 0.001 |
| 365 | 3.348014707 | 0.035386847 | 0.004 | 0.038884712 | 0.843700894 | 0 | 0.419354642 | 0.657539222 | 0 |
| 366 | 7.333582109 | 0.000674178 | 0.009 | 6.36327063 | 0.011741602 | 0.004 | 0.019576616 | 0.980613983 | 0 |
| 367 | 2.900031115 | 0.055294727 | 0.003 | 0.904998833 | 0.341580575 | 0.001 | 1.734188648 | 0.176856654 | 0.002 |
| 368 | 0.936364124 | 0.392253515 | 0.001 | 3.605136395 | 0.057771125 | 0.002 | 0.753728609 | 0.47076632 | 0.001 |
| 369 | 2.45812313 | 0.085900718 | 0.003 | 2.694772512 | 0.100863479 | 0.002 | 0.481988057 | 0.617639101 | 0.001 |
| 370 | 1.27523806 | 0.279632503 | 0.002 | 1.336618961 | 0.247794125 | 0.001 | 0.090793138 | 0.913211043 | 0 |
| 371 | 2.648214159 | 0.071070507 | 0.003 | 0.097777836 | 0.754551281 | 0 | 1.32283327 | 0.26665453 | 0.002 |
| 372 | 4.158423825 | 0.015792044 | 0.005 | 8.066431463 | 0.004563161 | 0.005 | 0.235755096 | 0.790000033 | 0 |
| 373 | 0.353212478 | 0.702479647 | 0 | 0.073096928 | 0.786912832 | 0 | 0.461147584 | 0.630638744 | 0.001 |
| 374 | 0.32030414 | 0.725972175 | 0 | 11.94463014 | 0.000561579 | 0.007 | 3.364355141 | 0.034815554 | 0.004 |
| 375 | 1.247711769 | 0.2874249 | 0.001 | 0.001018949 | 0.974538821 | 0 | 0.331256001 | 0.718067831 | 0 |
| 376 | 1.53911785 | 0.214870202 | 0.002 | 3.22480243 | 0.0727087 | 0.002 | 0.328606555 | 0.719972093 | 0 |
| 377 | 0.156930817 | 0.854775617 | 0 | 2.297904281 | 0.129735257 | 0.001 | 0.025442046 | 0.974879248 | 0 |
| 378 | 0.729383757 | 0.482357428 | 0.001 | 1.171920263 | 0.279161097 | 0.001 | 0.303125352 | 0.738546567 | 0 |
| 379 | 0.307240353 | 0.735514781 | 0 | 0.294021745 | 0.587726073 | 0 | 1.976323578 | 0.138897162 | 0.002 |
| 380 | 1.796320985 | 0.166224025 | 0.002 | 3.181538487 | 0.074653546 | 0.002 | 2.067500227 | 0.126820703 | 0.002 |
| 381 | 0.41742295 | 0.658809984 | 0 | 3.845100559 | 0.050055162 | 0.002 | 0.76177741 | 0.466995783 | 0.001 |
| 382 | 4.966250319 | 0.007071041 | 0.006 | 4.194042057 | 0.040720158 | 0.002 | 2.80540506 | 0.060763299 | 0.003 |
| 383 | 1.805586453 | 0.164694233 | 0.002 | 0.001260756 | 0.971679544 | 0 | 0.997152893 | 0.369144766 | 0.001 |
| 384 | 2.736545601 | 0.065080235 | 0.003 | 0.081344567 | 0.775518363 | 0 | 0.382113124 | 0.682476655 | 0 |
| 385 | 2.38685928 | 0.092226988 | 0.003 | 7.034564426 | 0.008069757 | 0.004 | 2.688348662 | 0.068283214 | 0.003 |
| 386 | 1.812133574 | 0.163621764 | 0.002 | 0.841191511 | 0.359186774 | 0 | 0.460601117 | 0.630983274 | 0.001 |
| 387 | 3.219640158 | 0.040214151 | 0.004 | 6.837708345 | 0.009004509 | 0.004 | 0.766222137 | 0.464926583 | 0.001 |
| 388 | 0.582797887 | 0.558445932 | 0.001 | 2.034818242 | 0.153916828 | 0.001 | 0.04966322 | 0.951551218 | 0 |
| 389 | 1.998011903 | 0.135924048 | 0.002 | 0.918682561 | 0.3379579 | 0.001 | 0.332378022 | 0.717262911 | 0 |
| 390 | 3.97848059 | 0.01888918 | 0.005 | 8.042233148 | 0.004624221 | 0.005 | 1.479915158 | 0.227951184 | 0.002 |
| 391 | 1.486927446 | 0.226361092 | 0.002 | 0.203400878 | 0.652047369 | 0 | 0.300853575 | 0.740225688 | 0 |
| 392 | 0.838593454 | 0.432497561 | 0.001 | 3.58697194 | 0.058404487 | 0.002 | 4.10582436 | 0.016640681 | 0.005 |
| 393 | 5.165058466 | 0.005803038 | 0.006 | 9.99249375 | 0.001599608 | 0.006 | 0.359823489 | 0.697852805 | 0 |
| 394 | 2.754392109 | 0.063932769 | 0.003 | 6.238492331 | 0.012594496 | 0.004 | 3.125848496 | 0.044152988 | 0.004 |
| 395 | 1.189857059 | 0.30451891 | 0.001 | 8.71702114 | 0.003195913 | 0.005 | 3.438005712 | 0.032353048 | 0.004 |
| 396 | 3.521703956 | 0.029765573 | 0.004 | 10.16012226 | 0.001461299 | 0.006 | 4.808093995 | 0.008275147 | 0.006 |
| 397 | 5.629866674 | 0.003656537 | 0.007 | 12.34678987 | 0.000453351 | 0.007 | 0.814416266 | 0.443071123 | 0.001 |
| 398 | 5.37965138 | 0.004688516 | 0.006 | 1.411589961 | 0.234959175 | 0.001 | 0.629624409 | 0.532916511 | 0.001 |
| 399 | 8.786085019 | 0.000159921 | 0.01 | 7.302722453 | 0.006953749 | 0.004 | 1.560176465 | 0.210400736 | 0.002 |
| 400 | 8.559144578 | 0.000200201 | 0.01 | 5.848201798 | 0.015697845 | 0.003 | 0.991370383 | 0.371283016 | 0.001 |

**Table S7.** Statistical information on Frontoparietal Control, Default, and Salience/Ventral Attention at different developmental stages.

|  |  |  |  |  | **95% Confidence Interval for Mean** | | **unpaired two-sided *t*-test** | | | |
| --- | --- | --- | --- | --- | --- | --- | --- | --- | --- | --- |
|  |  |  | **Mean** | **SD** | **Lower Bound** | **Upper Bound** | ***t-stat*** | ***p*** | **Bonferroni** | **Cohen's *d*** |
| Frontoparietal Control | Children | ASD | -0.3177 | 1.0447 | -0.4399 | -0.1955 | 1.8086 | 0.071 | 0.213 | 0.081 |
|  |  | TC | -0.3998 | 1.0195 | -0.5175 | -0.282 |  |  |  |  |
|  | Adolescents | ASD | -0.2846 | 1.0127 | -0.4067 | -0.1624 | 3.8231 | 0.00014705 | 0.00044115 | 0.171 |
|  |  | TC | -0.4579 | 0.982 | -0.5759 | -0.34 |  |  |  |  |
|  | Adults | ASD | -0.3357 | 1.0236 | -0.4579 | -0.2135 | 2.0012 | 0.0459 | 0.1377 | 0.092 |
|  |  | TC | -0.4292 | 0.9964 | -0.547 | -0.3113 |  |  |  |  |
| Default | Children | ASD | -0.1825 | 0.9974 | -0.2985 | -0.0664 | 2.8124 | 0.0051 | 0.0153 | 0.142 |
|  |  | TC | -0.3223 | 0.9953 | -0.4342 | -0.2104 |  |  |  |  |
|  | Adolescents | ASD | -0.2458 | 0.9633 | -0.3618 | -0.1299 | 3.3328 | 0.00091824 | 0.00275472 | 0.174 |
|  |  | TC | -0.4168 | 1.0029 | -0.5289 | -0.3048 |  |  |  |  |
|  | Adults | ASD | -0.3047 | 0.965 | -0.4207 | -0.1886 | 2.0054 | 0.0454 | 0.1362 | 0.099 |
|  |  | TC | -0.4026 | 0.9829 | -0.5145 | -0.2906 |  |  |  |  |
| Salience/Ventral Attention | Children | ASD | 0.3713 | 0.8956 | 0.2649 | 0.4777 | -3.1924 | 0.0015 | 0.0045 | 0.158 |
|  |  | TC | 0.5097 | 0.8642 | 0.4073 | 0.612 |  |  |  |  |
|  | Adolescents | ASD | 0.385 | 0.9052 | 0.2787 | 0.4913 | -3.8675 | 0.00012327 | 0.00036981 | 0.202 |
|  |  | TC | 0.5615 | 0.8367 | 0.459 | 0.664 |  |  |  |  |
|  | Adults | ASD | 0.4106 | 0.8793 | 0.3042 | 0.5169 | -2.659 | 0.0081 | 0.0243 | 0.139 |
|  |  | TC | 0.5324 | 0.8678 | 0.4299 | 0.6348 |  |  |  |  |

**Supplementary Figures**

**Figure S1.** The shift in sliding window standard deviation (SWSTD) corresponds to the shift in FCD. (A) The two states of FCD were simulated, with the coherent state exhibiting FC patterns over time characterized by large coherent amplitudes in the regional fMRI signal, corresponding to a high standard deviation. The incoherent state exhibits an FC pattern over time characterized by noise in the regional fMRI, corresponding to a low standard deviation. (B) shows the FCD and SWSTD of a single subject randomly selected from the dataset. The top panel represents the individual's FCD matrix. The middle panel represents the average time course of FCD obtained by averaging the rows or columns of the FCD matrix. The bottom panel indicates the standard deviation of the fMRI time course for each region. The red dashed line indicates that sharp transitions in sliding window standard deviation (SWSTD) correspond to sharp FCD transitions.


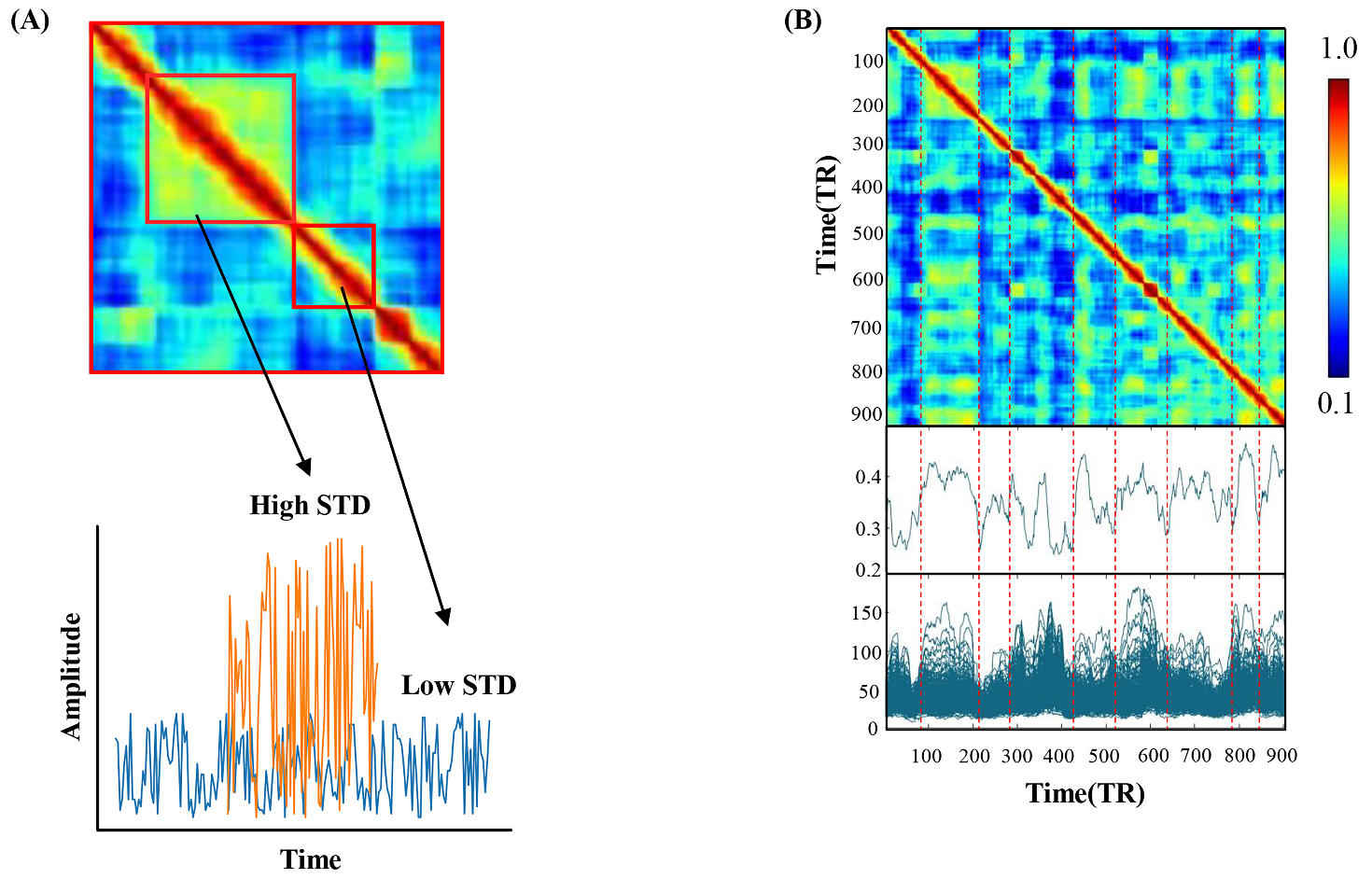


**Figure S2.** Overlapping brain regions correspond to clinical symptoms. (A) ANOVA interaction effects between Age and Group (with threshold map $p_{uncorr}$ < 0.05) (B) The two groups (severe and mild group) of subjects above and below the overall score mean were significantly different in five brain regions after an unpaired two-sided *t*-test. ROIs with significant differences in the brain space map are shown in different colored blocks. The bar graph shows the mean value of high group and low group. Areas associated with clinical symptoms ADI_RRB_ToTal_C (Restricted, Repetitive, and Stereotyped Patterns of Behavior Subscore (C)Total for Autism Diagnostic Interview-Revised). (C) same as (B). Areas associated with clinical symptoms ADI_R_ONSET_TOTAL_C (Abnormality of Development Evident at or Before 36 Months Subscore (D)Total for Autism Diagnostic Interview-Revised).


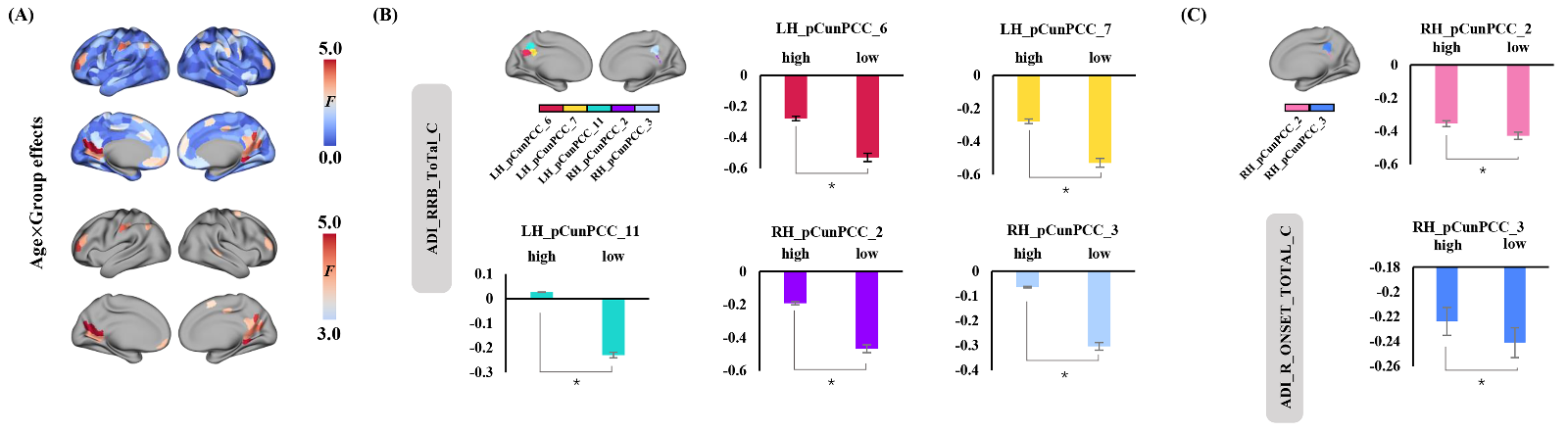


**Figure S3.** Differential analysis of brain functional dynamics in different age groups. (A) Top-to-bottom spatial maps representing ASD versus TC brain dynamics at different time periods, obtained by calculating group-averaged rFSI, with high correlation values clustered in unimodal regions. (B) Differential Patterns of ASD-TC in childhood, adolescence, and adulthood (C) Spatial correlation (Pearson’s *r*) of the difference patterns in the three periods, with correlations between t-value maps of children and adolescents (*r* = 0.53) higher than correlations between adolescents and adults (*r* = 0.31), and correlations between children and adults being lowest (*r* = 0.15). (D) Comparison of correlation coefficient *r*-values. * indicate *p* < 0.05, *** indicate *p* < 0.001.


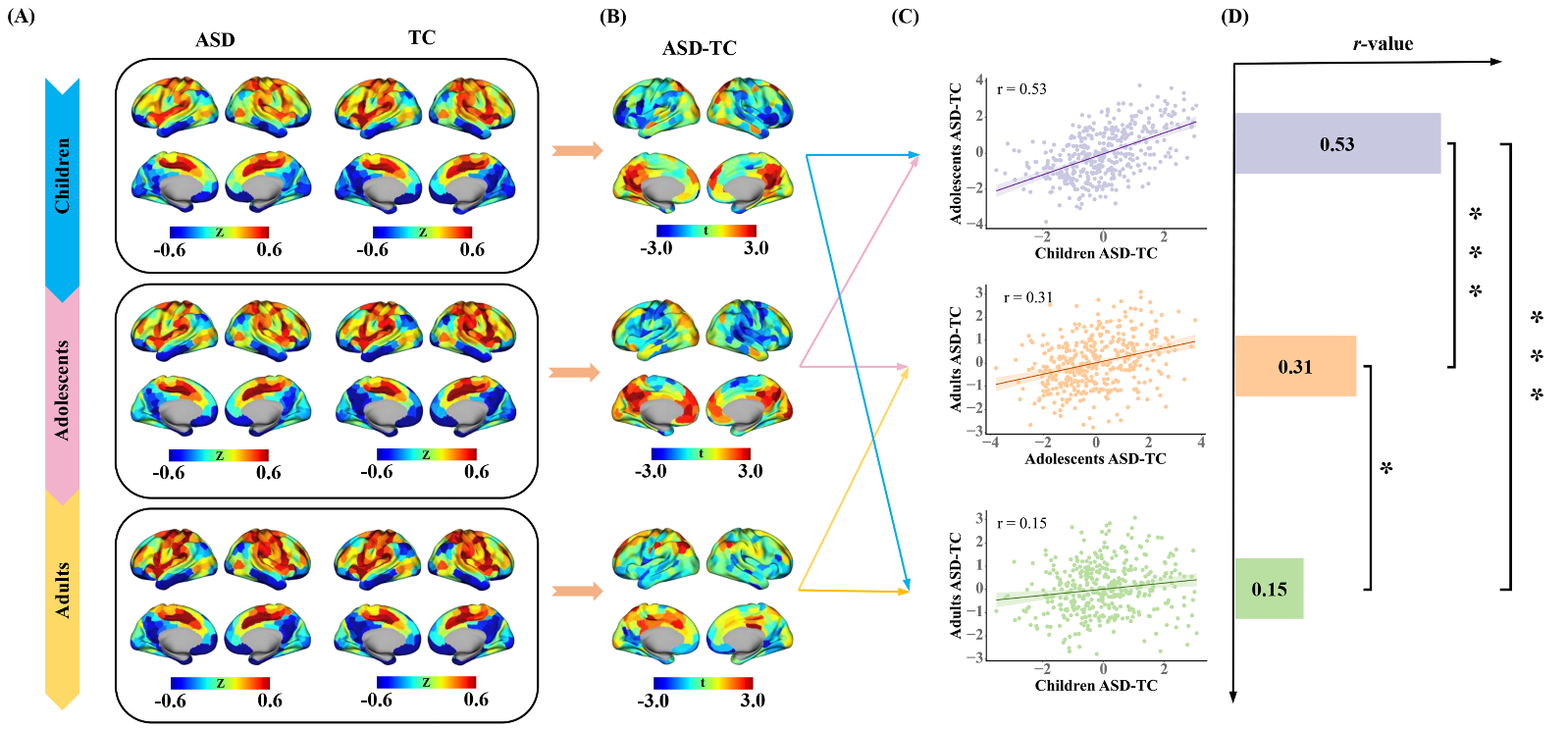


**Figure S4.** Decoding of Age effects in brain function. Relationships between Age mapping and 24 cognitive components based on the NeuroSynth meta-analysis database. Each row indicates that the components are sorted from left to right by five percent increments of the F-statistic. Each column indicates that the cognitive components were sorted by the weighted average of the resulting z-statistic values.


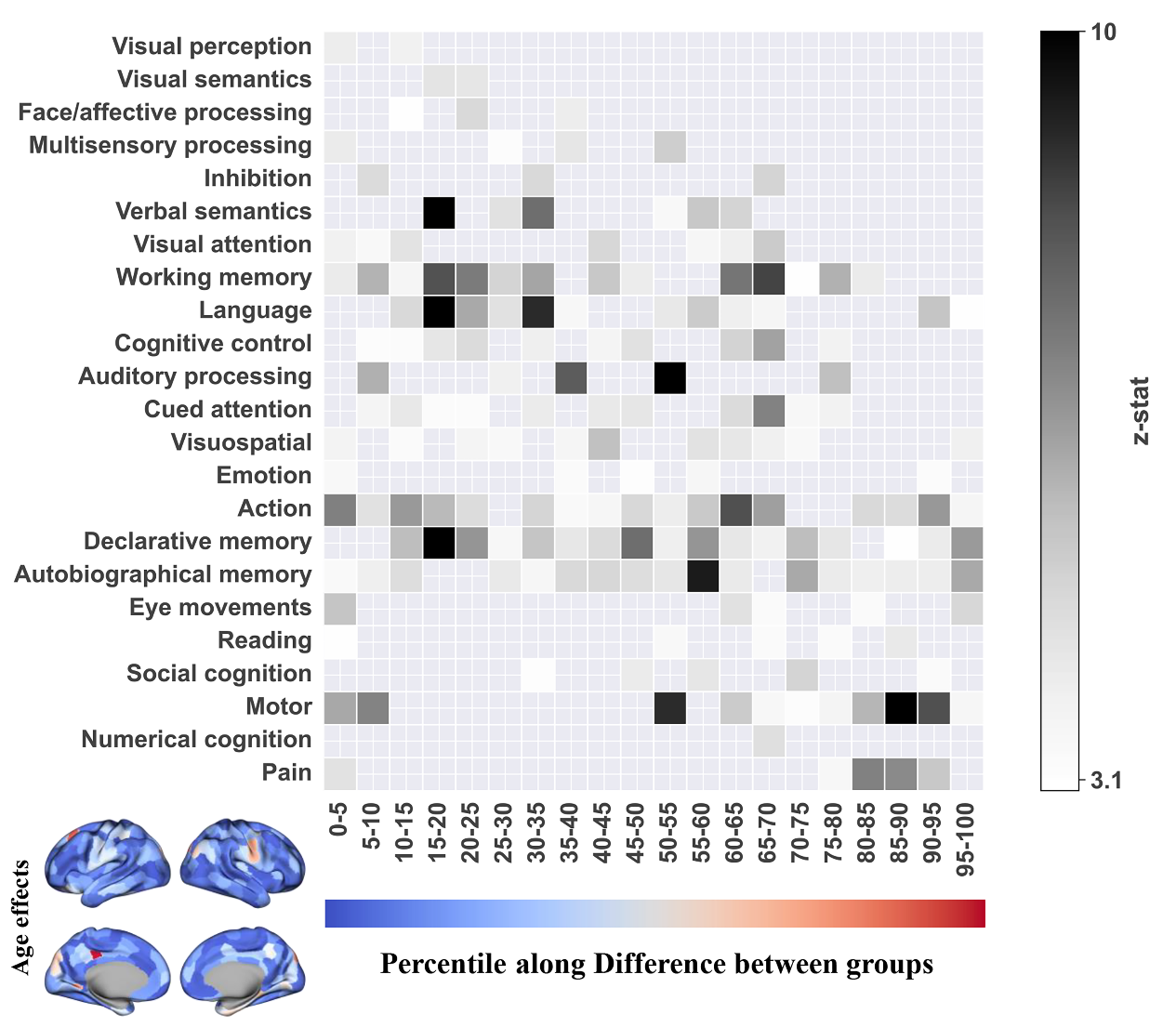


**Figure S5.** Gene expression profiles associated with changes in ASD brain dynamics. (A) Ability of the first 15 components to explain variance. (B) Significance assessment of PLS results as a function of number of first 8 components. (C) Spatial correlation between PLS1 and PLS2 scores (weighted sum of 15633 gene expression scores) in left hemisphere regions and case-control differences (Group effects F-map) ($r_{1}$ = 0.41, $p_{spin}$ < 0.005; $r_{2}$ = 0.31, $p_{spin}$ < 0.005; permutation 10,000 times). (D) Spatial maps of PLS1 and PLS2 scores (Deleted ROIs are filled in using linear interpolation). (E) Gene set enrichment analysis of genes associated with dynamic sexual variation in the ASD brain. PLS1+ genes (*z* > 2.8, $p_{FDR}$ < 0.05) were selected for gene enrichment analysis; the size of the circle indicates the ratio of the number of genes involved in a given term, and the color represents the significance level of the enrichment.


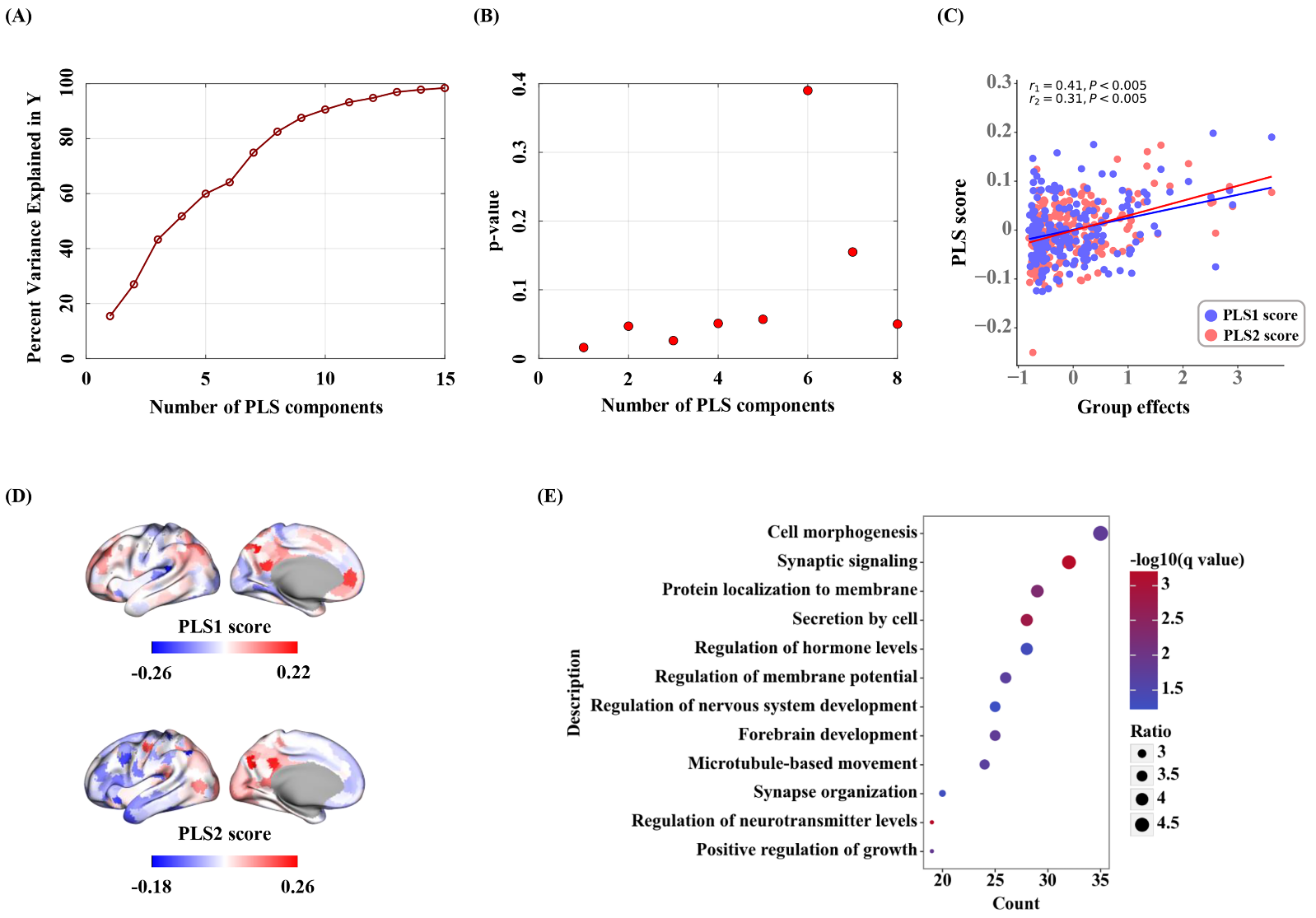


**Figure S6.** SnRNA-seq data preprocessing. (A) The proportion of cells removed due to ambient RNA contamination in different samples. (B) The proportion of doublet cells removed in different samples. (C) Distribution of the number of genes and transcripts per nucleus in the ASD and TC groups. (D) Violin distribution of mitochondrial gene transcription ratios within each cluster after quality control.


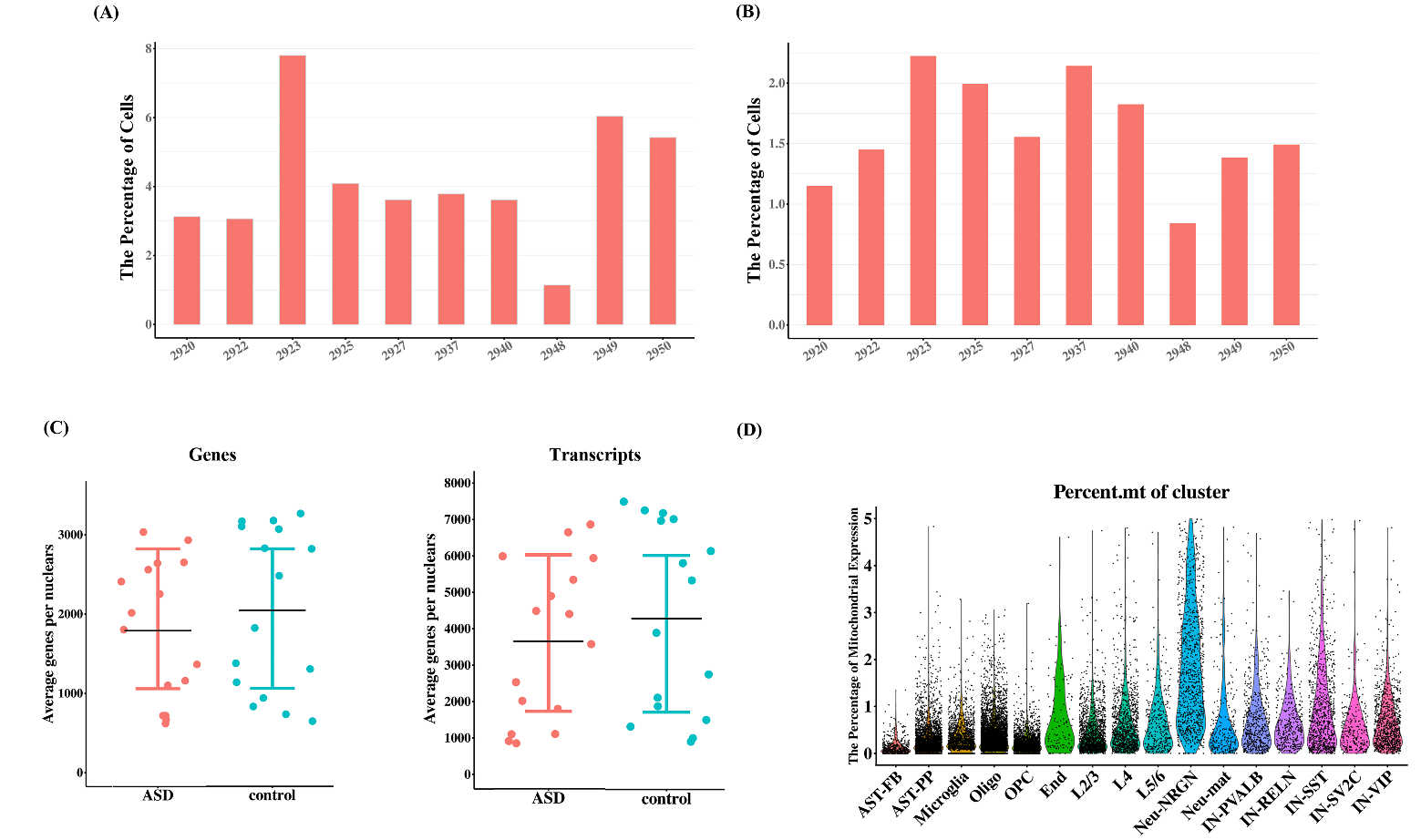


**Figure S7.** Feature plot for different markers across different cell types.


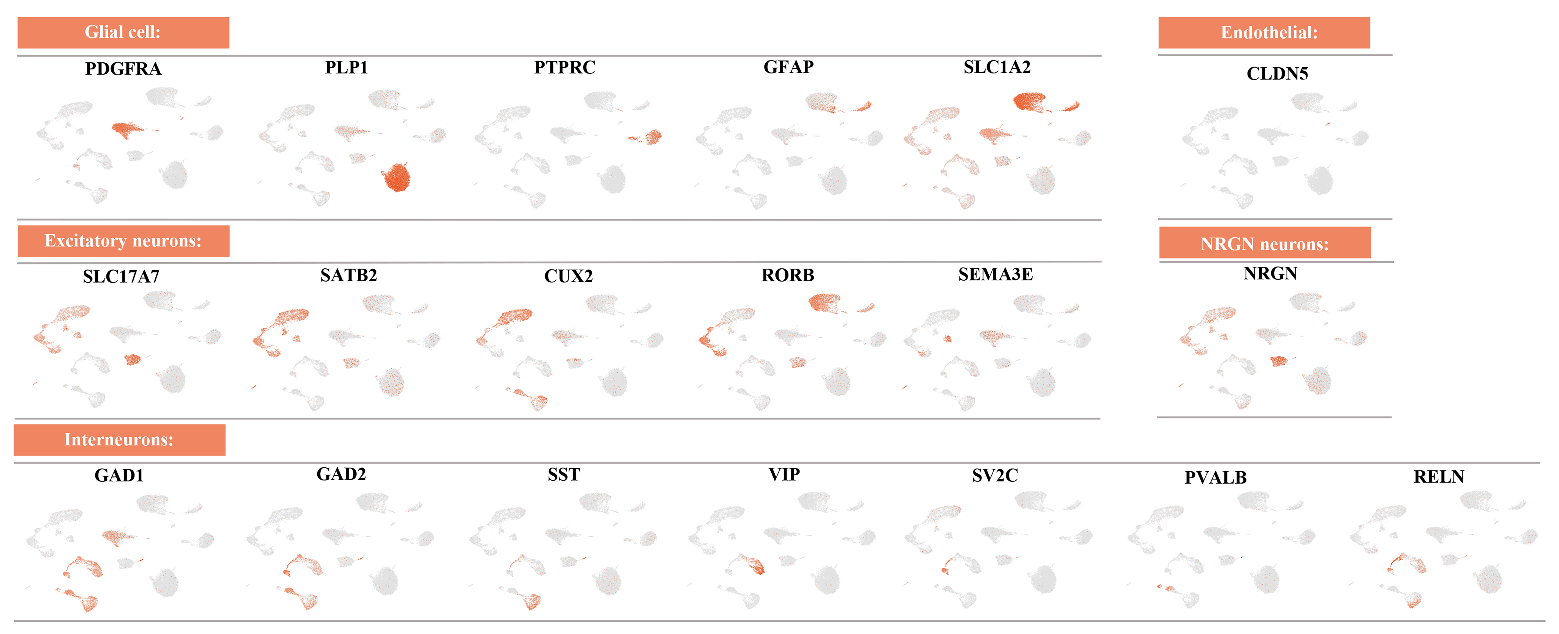


**Figure S8.** Distribution of cell number and percentage per clusters. (A-C) Histogram of the distribution of the number of cells per cell population in children, adolescents and adults (D) Stacked histogram of the percentage of cells per subgroup in each cluster.


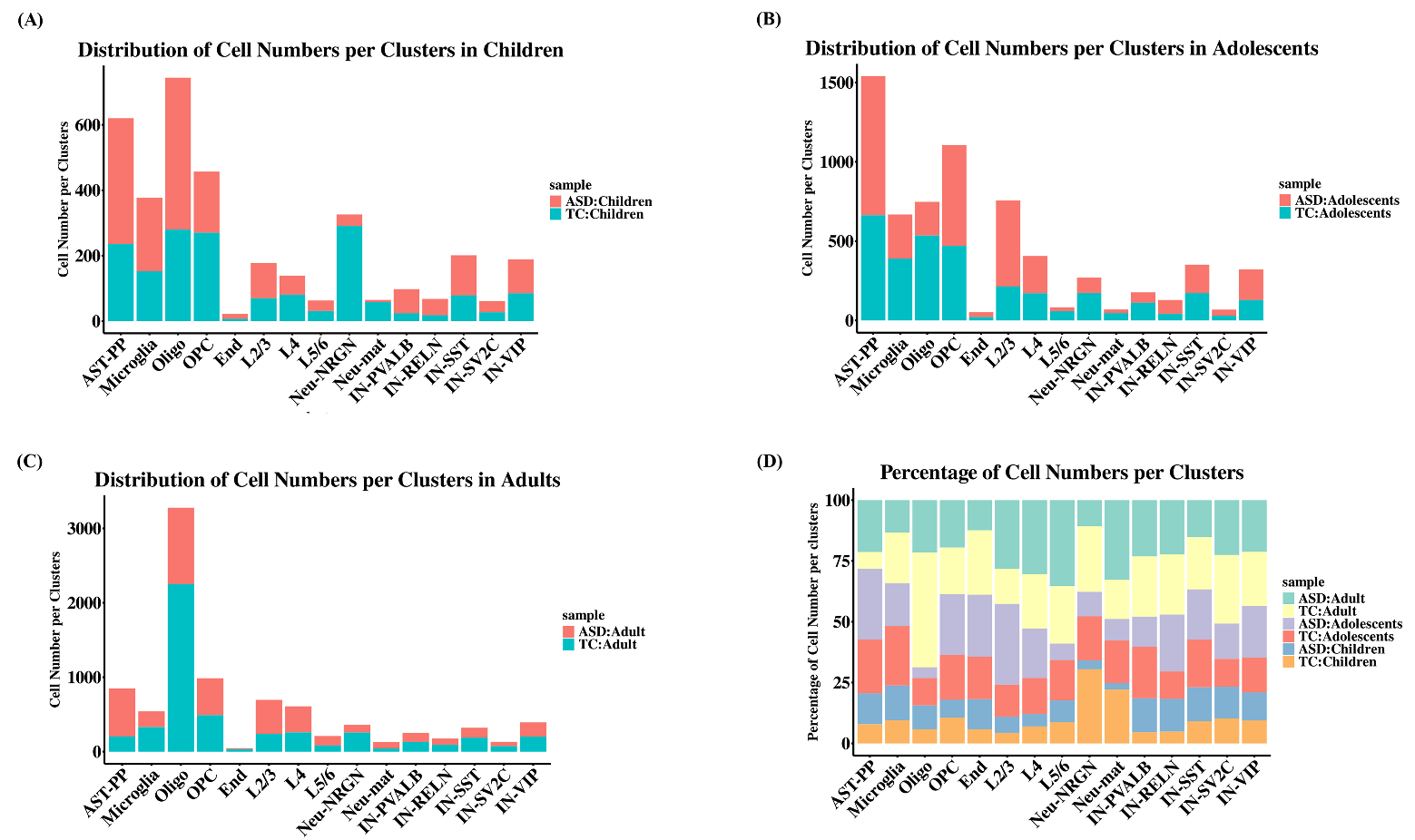


**Figure S9.** GO enrichment analysis. (A) GO enrichment analysis of differentially expressed genes (DEGs) in children, adolescents and adults. (B) GO enrichment of up-regulated and down-regulated DEGs in different age groups (excluding overlapping gene sets).


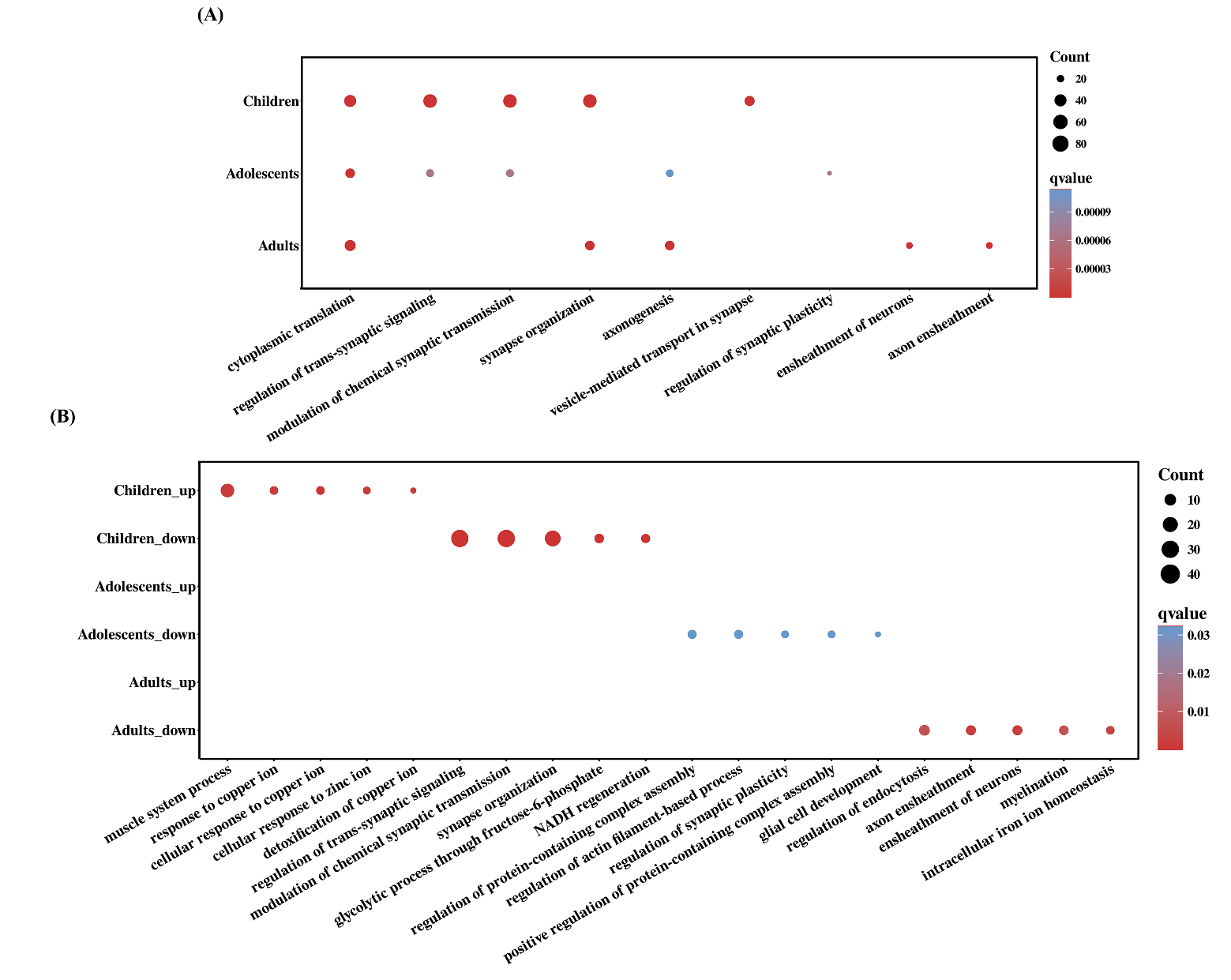


**Figure S10.** Pseudotime analysis. (A) shows the pseudotime trajectory. Darker to lighter colors indicate earlier to later differentiation (B) Pseudotime trajectories of cell differentiation. Different colors indicate cells in ASD and TC. (C) Pseudotime trajectories of cell differentiation. Different colors indicate different ages. (D) Different colors indicate cells in 6 different subgroups.


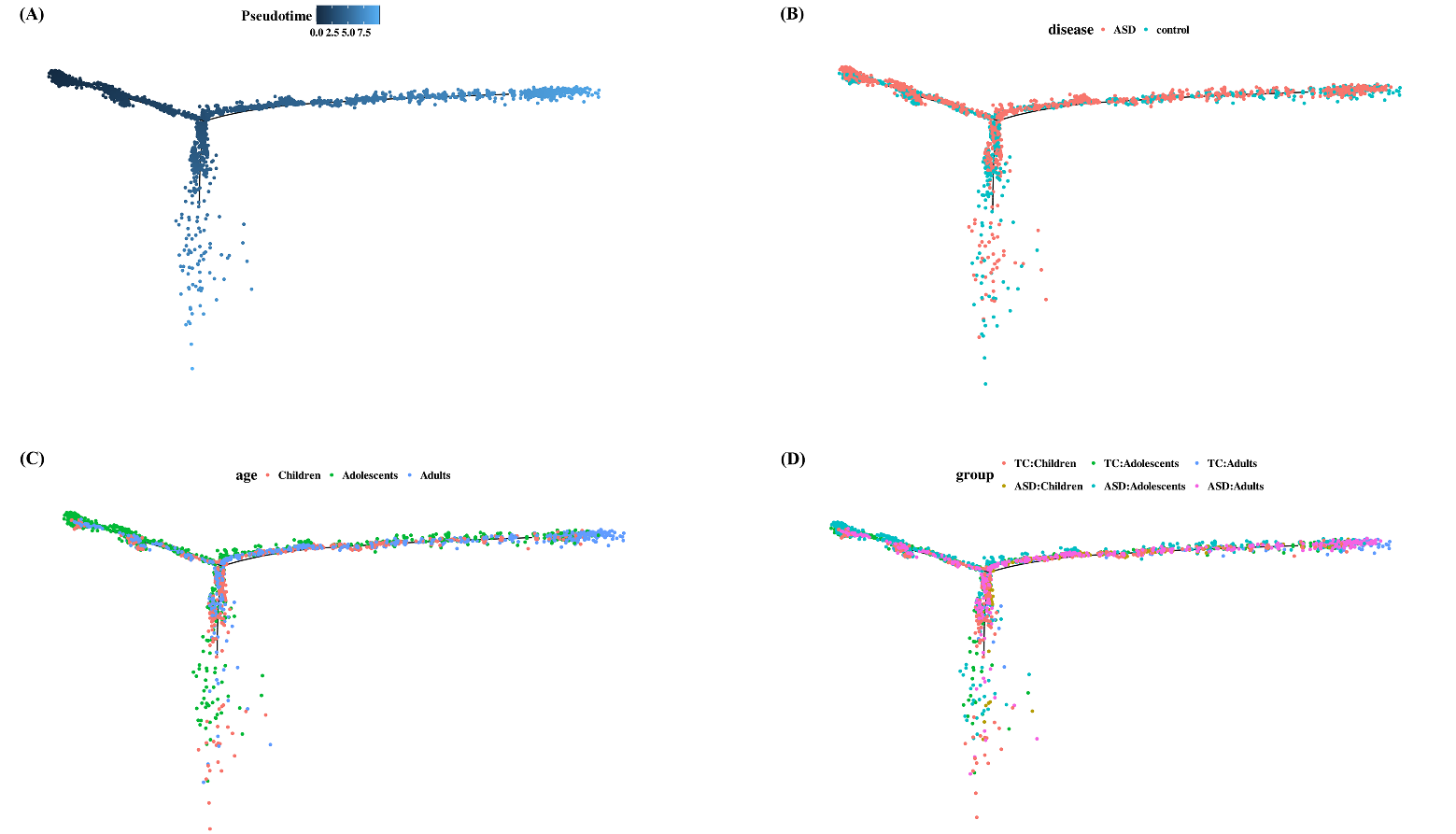


**Figure S11.** Verification analysis. (A-C) Spatial difference pattern maps (unpaired two-sided *t*-test ASD-TC) for children, adolescents, and adults at window lengths of 24 and 36, respectively, and at higher resolution scales (Scheafer anatomical parcellation with cortical 800 ROIs).


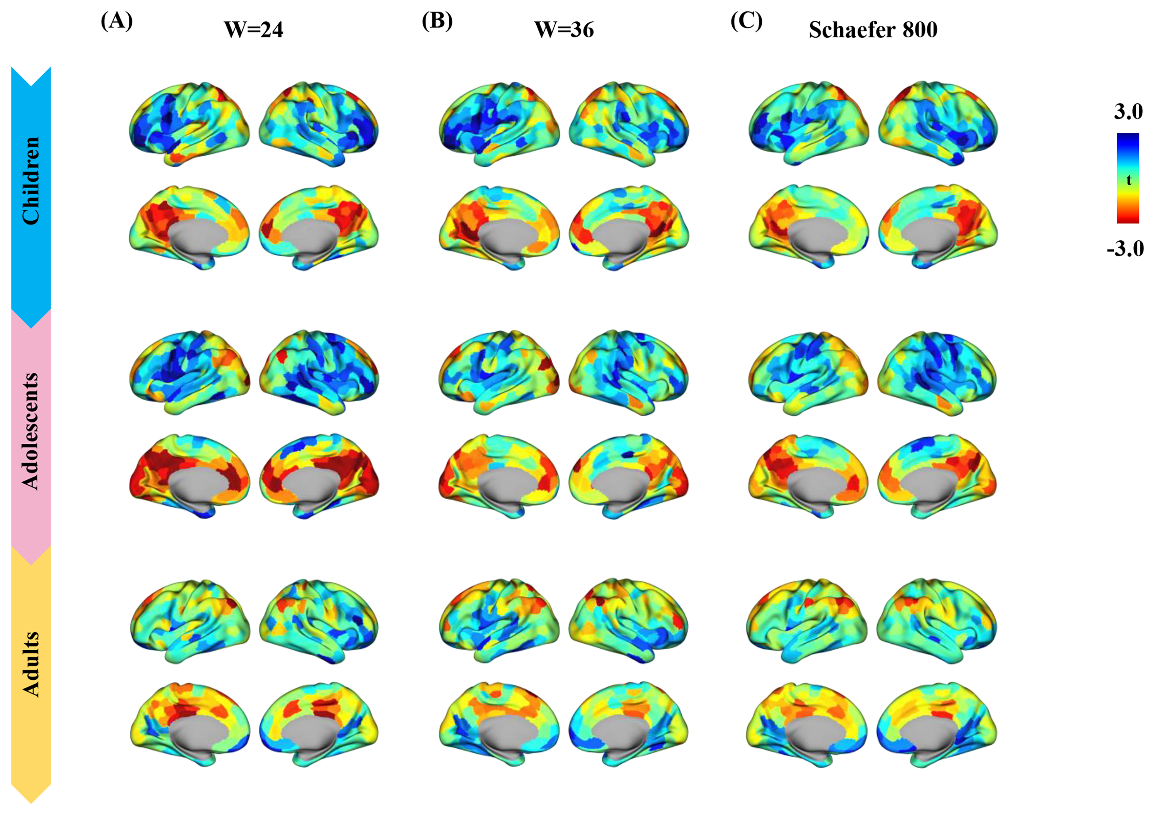


**References**

Aghourian, M., Legault-Denis, C., Soucy, J.P., Rosa-Neto, P., Gauthier, S., Kostikov, A., Gravel, P., Bédard, M.A., 2017. Quantification of brain cholinergic denervation in Alzheimer’s disease using PET imaging with [18F]-FEOBV. Molecular psychiatry 22, 1531–1538.

Alakurtti, K., Johansson, J.J., Joutsa, J., Laine, M., Bäckman, L., Nyberg, L., Rinne, J.O., 2015. Long-Term Test–Retest Reliability of Striatal and Extrastriatal Dopamine D _2/3_ Receptor Binding: Study with [ ^11^ C]Raclopride and High-Resolution PET. J Cereb Blood Flow Metab 35, 1199–1205. https://doi.org/10.1038/jcbfm.2015.53

Bedard, M.-A., Aghourian, M., Legault-Denis, C., Postuma, R.B., Soucy, J.-P., Gagnon, J.-F., Pelletier, A., Montplaisir, J., 2019. Brain cholinergic alterations in idiopathic rem sleep behaviour disorder: a pet imaging study with 18f-feobv. Sleep medicine 58, 35–41.

Beliveau, V., Ganz, M., Feng, L., Ozenne, B., Højgaard, L., Fisher, P.M., Svarer, C., Greve, D.N., Knudsen, G.M., 2017. A high-resolution in vivo atlas of the human brain’s serotonin system. Journal of Neuroscience 37, 120–128.

Ding, Y.-S., Singhal, T., Planeta-Wilson, B., Gallezot, J.-D., Nabulsi, N., Labaree, D., Ropchan, J., Henry, S., Williams, W., Carson, R.E., Neumeister, A., Malison, R.T., 2010. PET imaging of the effects of age and cocaine on the norepinephrine transporter in the human brain using (S,S)-[ ^11^ C]O-methylreboxetine and HRRT. Synapse 64, 30–38. https://doi.org/10.1002/syn.20696

DuBois, J.M., Rousset, O.G., Rowley, J., Porras-Betancourt, M., Reader, A.J., Labbe, A., Massarweh, G., Soucy, J.-P., Rosa-Neto, P., Kobayashi, E., 2016. Characterization of age/sex and the regional distribution of mGluR5 availability in the healthy human brain measured by high-resolution [11C]ABP688 PET. Eur J Nucl Med Mol Imaging 43, 152–162. https://doi.org/10.1007/s00259-015-3167-6

Dukart, J., Holiga, Š., Chatham, C., Hawkins, P., Forsyth, A., McMillan, R., Myers, J., Lingford-Hughes, A.R., Nutt, D.J., Merlo-Pich, E., Risterucci, C., Boak, L., Umbricht, D., Schobel, S., Liu, T., Mehta, M.A., Zelaya, F.O., Williams, S.C., Brown, G., Paulus, M., Honey, G.D., Muthukumaraswamy, S., Hipp, J., Bertolino, A., Sambataro, F., 2018. Cerebral blood flow predicts differential neurotransmitter activity. Sci Rep 8, 4074. https://doi.org/10.1038/s41598-018-22444-0

Fazio, P., Schain, M., Varnäs, K., Halldin, C., Farde, L., Varrone, A., 2016. Mapping the distribution of serotonin transporter in the human brainstem with high-resolution PET: validation using postmortem autoradiography data. Neuroimage 133, 313–320.

Finnema, S.J., Nabulsi, N.B., Mercier, J., Lin, S., Chen, M.-K., Matuskey, D., Gallezot, J.-D., Henry, S., Hannestad, J., Huang, Y., Carson, R.E., 2018. Kinetic evaluation and test–retest reproducibility of [ ^11^ C]UCB-J, a novel radioligand for positron emission tomography imaging of synaptic vesicle glycoprotein 2A in humans. J Cereb Blood Flow Metab 38, 2041–2052. https://doi.org/10.1177/0271678X17724947

Gallezot, J.-D., Nabulsi, N., Neumeister, A., Planeta-Wilson, B., Williams, W.A., Singhal, T., Kim, S., Maguire, R.P., McCarthy, T., Frost, J.J., Huang, Y., Ding, Y.-S., Carson, R.E., 2010. Kinetic Modeling of the Serotonin 5-HT _1B_ Receptor Radioligand [ ^11^ C]P943 in Humans. J Cereb Blood Flow Metab 30, 196–210. https://doi.org/10.1038/jcbfm.2009.195

Gallezot, J.-D., Planeta, B., Nabulsi, N., Palumbo, D., Li, X., Liu, J., Rowinski, C., Chidsey, K., Labaree, D., Ropchan, J., Lin, S.-F., Sawant-Basak, A., McCarthy, T.J., Schmidt, A.W., Huang, Y., Carson, R.E., 2017. Determination of receptor occupancy in the presence of mass dose: [ ^11^ C]GSK189254 PET imaging of histamine H _3_ receptor occupancy by PF-03654746. J Cereb Blood Flow Metab 37, 1095–1107. https://doi.org/10.1177/0271678X16650697

Hansen, J.Y., Shafiei, G., Markello, R.D., Smart, K., Cox, S.M., Nørgaard, M., Beliveau, V., Wu, Y., Gallezot, J.-D., Aumont, É., 2022. Mapping neurotransmitter systems to the structural and functional organization of the human neocortex. Nature neuroscience 25, 1569–1581.

Hesse, S., Becker, G.-A., Rullmann, M., Bresch, A., Luthardt, J., Hankir, M.K., Zientek, F., Reißig, G., Patt, M., Arelin, K., Lobsien, D., Müller, U., Baldofski, S., Meyer, P.M., Blüher, M., Fasshauer, M., Fenske, W.K., Stumvoll, M., Hilbert, A., Ding, Y.-S., Sabri, O., 2017. Central noradrenaline transporter availability in highly obese, non-depressed individuals. Eur J Nucl Med Mol Imaging 44, 1056–1064. https://doi.org/10.1007/s00259-016-3590-3

Hillmer, A.T., Esterlis, I., Gallezot, J.-D., Bois, F., Zheng, M.-Q., Nabulsi, N., Lin, S.-F., Papke, R.L., Huang, Y., Sabri, O., 2016. Imaging of cerebral α4β2* nicotinic acetylcholine receptors with (-)-[18F] Flubatine PET: Implementation of bolus plus constant infusion and sensitivity to acetylcholine in human brain. Neuroimage 141, 71–80.

Jaworska, N., Cox, S.M., Tippler, M., Castellanos-Ryan, N., Benkelfat, C., Parent, S., Dagher, A., Vitaro, F., Boivin, M., Pihl, R.O., 2020. Extra-striatal D2/3 receptor availability in youth at risk for addiction. Neuropsychopharmacology 45, 1498–1505.

Kaller, S., Rullmann, M., Patt, M., Becker, G.-A., Luthardt, J., Girbardt, J., Meyer, P.M., Werner, P., Barthel, H., Bresch, A., Fritz, T.H., Hesse, S., Sabri, O., 2017. Test–retest measurements of dopamine D1-type receptors using simultaneous PET/MRI imaging. Eur J Nucl Med Mol Imaging 44, 1025–1032. https://doi.org/10.1007/s00259-017-3645-0

Kantonen, T., Karjalainen, T., Isojärvi, J., Nuutila, P., Tuisku, J., Rinne, J., Hietala, J., Kaasinen, V., Kalliokoski, K., Scheinin, H., 2020. Interindividual variability and lateralization of μ-opioid receptors in the human brain. Neuroimage 217, 116922.

Laurikainen, H., Tuominen, L., Tikka, M., Merisaari, H., Armio, R.-L., Sormunen, E., Borgan, F., Veronese, M., Howes, O., Haaparanta-Solin, M., 2019. Sex difference in brain CB1 receptor availability in man. Neuroimage 184, 834–842.

Naganawa, M., Nabulsi, N., Henry, S., Matuskey, D., Lin, S.-F., Slieker, L., Schwarz, A.J., Kant, N., Jesudason, C., Ruley, K., 2021. First-in-human assessment of 11c-lsn3172176, an m1 muscarinic acetylcholine receptor pet radiotracer. Journal of Nuclear Medicine 62, 553–560.

Nørgaard, M., Beliveau, V., Ganz, M., Svarer, C., Pinborg, L.H., Keller, S.H., Jensen, P.S., Greve, D.N., Knudsen, G.M., 2021. A high-resolution in vivo atlas of the human brain’s benzodiazepine binding site of GABAA receptors. NeuroImage 232, 117878.

Normandin, M.D., Zheng, M.-Q., Lin, K.-S., Mason, N.S., Lin, S.-F., Ropchan, J., Labaree, D., Henry, S., Williams, W.A., Carson, R.E., Neumeister, A., Huang, Y., 2015. Imaging the Cannabinoid CB1 Receptor in Humans with [ ^11^ C] OMAR: Assessment of Kinetic Analysis Methods, Test–Retest Reproducibility, and Gender Differences. J Cereb Blood Flow Metab 35, 1313–1322. https://doi.org/10.1038/jcbfm.2015.46

Radhakrishnan, R., Nabulsi, N., Gaiser, E., Gallezot, J.-D., Henry, S., Planeta, B., Lin, S., Ropchan, J., Williams, W., Morris, E., 2018. Age-related change in 5-ht6 receptor availability in healthy male volunteers measured with 11c-gsk215083 pet. Journal of Nuclear Medicine 59, 1445–1450.

Sandiego, C.M., Gallezot, J.-D., Lim, K., Ropchan, J., Lin, S., Gao, H., Morris, E.D., Cosgrove, K.P., 2015. Reference Region Modeling Approaches for Amphetamine Challenge Studies with [ ^11^ C]FLB 457 and PET. J Cereb Blood Flow Metab 35, 623–629. https://doi.org/10.1038/jcbfm.2014.237

Sasaki, T., Ito, H., Kimura, Y., Arakawa, R., Takano, H., Seki, C., Kodaka, F., Fujie, S., Takahata, K., Nogami, T., 2012. Quantification of dopamine transporter in human brain using PET with 18F-FE-PE2I. Journal of Nuclear Medicine 53, 1065–1073.

Savli, M., Bauer, A., Mitterhauser, M., Ding, Y.-S., Hahn, A., Kroll, T., Neumeister, A., Haeusler, D., Ungersboeck, J., Henry, S., 2012. Normative database of the serotonergic system in healthy subjects using multi-tracer PET. Neuroimage 63, 447–459.

Smart, K., Cox, S.M.L., Scala, S.G., Tippler, M., Jaworska, N., Boivin, M., Séguin, J.R., Benkelfat, C., Leyton, M., 2019. Sex differences in [11C]ABP688 binding: a positron emission tomography study of mGlu5 receptors. Eur J Nucl Med Mol Imaging 46, 1179–1183. https://doi.org/10.1007/s00259-018-4252-4

Smith, C.T., Crawford, J.L., Dang, L.C., Seaman, K.L., San Juan, M.D., Vijay, A., Katz, D.T., Matuskey, D., Cowan, R.L., Morris, E.D., Zald, D.H., Samanez-Larkin, G.R., 2019. Partial-volume correction increases estimated dopamine D2-like receptor binding potential and reduces adult age differences. J Cereb Blood Flow Metab 39, 822–833. https://doi.org/10.1177/0271678X17737693

Turtonen, O., Saarinen, A., Nummenmaa, L., Tuominen, L., Tikka, M., Armio, R.-L., Hautamäki, A., Laurikainen, H., Raitakari, O., Keltikangas-Järvinen, L., Hietala, J., 2021. Adult Attachment System Links With Brain Mu Opioid Receptor Availability In Vivo. Biol Psychiatry Cogn Neurosci Neuroimaging 6, 360–369. https://doi.org/10.1016/j.bpsc.2020.10.013
